# Novel *cis*-selective and non-epimerisable C3 hydroxy azapodophyllotoxins targeting microtubules in cancer cells

**DOI:** 10.1016/j.ejmech.2015.12.037

**Published:** 2016-03-03

**Authors:** Sahar Kandil, Jennifer M. Wymant, Benson M. Kariuki, Arwyn T. Jones, Christopher McGuigan, Andrew D. Westwell

**Affiliations:** a Cardiff School of Pharmacy & Pharmaceutical Sciences, Cardiff University, King Edward VII Avenue, Cardiff, CF10 3NB, Wales, United Kingdom; b School of Chemistry, Cardiff University, Park Place, Cardiff, CF10 3AT, Wales, United Kingdom

**Keywords:** Podophyllotoxin (PT), 4-azapodophyllotoxin (APT), Tubulin, Epimerisation

## Abstract

Podophyllotoxin (PT) and its clinically used analogues are known to be powerful antitumour agents. These compounds contain a *trans* fused strained γ-lactone system, a feature that correlates to the process of epimerisation, whereby the *trans* γ-lactone system of ring *D* opens and converts to the more thermodynamically stable *cis* epimer. Since these *cis* epimers are known to be either less active or lacking antitumour activity, epimerisation is an undesirable feature from a chemotherapeutic point of view. To circumvent this problem, considerable efforts have been reported, amongst which is the synthesis of azapodophyllotoxins where the stereocentres at **C2** and **C3** are removed in order to preclude epimerisation. Herein we report the identification of a novel **C3** hydroxy, *cis-*selective γ-lactone configuration of ring *C* in the azapodophyllotoxin scaffold, through an efficient stereoselective multicomponent reaction (MCR) involving fluorinated and non-fluorinated aldehydes. This configuration releases the highly strained *trans* γ-lactone system in podophyllotoxin analogues into the more thermodynamically stable *cis* γ-lactone motif and yet retains significantly potent activity. These compounds were evaluated against the human cancer lines MCF-7 and 22Rv1 *in vitro*. Fourteen out of the seventeen tested compounds exhibited sub-micromolar activity with IC_50_ values in the range of 0.11–0.91 μM, which is comparable and in some cases better than the activity profile of etoposide in this assay. Interestingly, we obtained strong evidence from spectroscopic and X-ray data analyses that the previously reported structure of similar analogues is not accurate. Molecular modelling performed using the podophyllotoxin binding site on *β* tubulin revealed a novel binding mode of these analogues. Furthermore, sub-cellular study of our compounds using immunolabelling and confocal microscopy analyses showed strong microtubule disruptive activity, particularly in dividing cells.

## Highlights

• Multicomponent synthesis of novel non-epimerisable hydroxylated *cis-*lactone azapodophyllotoxins.
• X-ray structural analysis confirms stereoselectivity.
• Sub-micromolar activity in human breast and prostate cancer cell lines, comparable with clinical drug controls.
• Molecular modeling studies offer a rational explanation of the SARs observed.
• Strong microtubule-disrupting activity in dividing cells, characterized by immunolabelling and confocal microscopy.

## Graphical abstract

**Figure:**
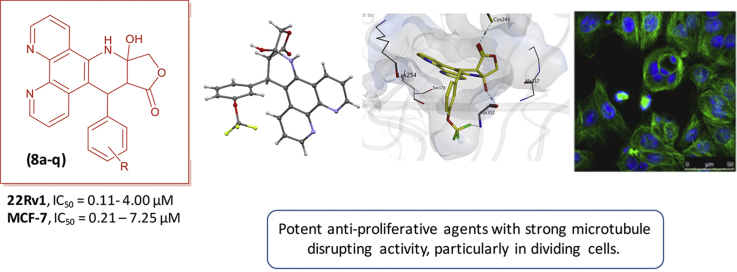

## 1 Introduction

**Fig. 1:**
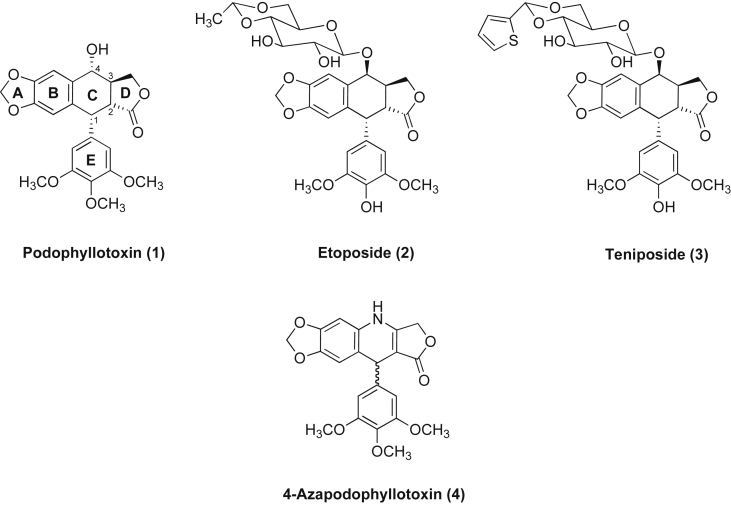
Structure of podophyllotoxin (**1**) and its mimetic scaffolds; etoposide (**2**) teniposide (**3**) and 4-azapodophyllotoxin (**4**).

Natural products are known to be evolutionary privileged structures. Historically, they have been an invaluable source of new pharmaceuticals. Almost 60% of anticancer drugs are derived from or related to natural products [1], [2]. Among these podophyllotoxin (PT) (**1**, Fig. 1) is a naturally occurring cyclolignan compound obtained from *podophyllum peltatum* and related species [3]. PT and its derivatives exhibit anticancer, antiviral and insecticidal activities due to their strong microtubule destabilising activity [4], [5], [6], [7]. Using PT as lead in anticancer drug discovery resulted in the development of semi-synthetic analogues such as etoposide (**2**) and teniposide (**3**) (Fig. 1), which are currently used either alone or in combination with other therapies for the treatment of a variety of malignancies including lung and testicular cancers, lymphoma, non-lymphocytic leukaemia, glioblastoma multiforme and childhood acute lymphocytic leukaemia [8]. Despite this there are some drawbacks associated with these agents such as the development of resistance, poor water solubility, metabolic inactivation and side effects like myelosuppression, neutropenia and nausea [8]. For these reasons, the search for new more effective podophyllotoxin analogues remains a highly valuable objective.

The PT structure was first elucidated in the 1930s and features four contiguous chiral centres on ring *C* (**C1** through **C4**) (Fig. 1). However, due to its structural complexity, the total synthesis of podophyllotoxin analogues is greatly hindered and most of the SAR studies of these analogues are limited to its semi-synthetic derivatives rather than novel chemical entities. The stereochemical intricacy and the associated tedious synthetic approaches, together with the liability of *in vivo* epimerisation and formation of the less potent *cis* lactone metabolite, have fuelled further investigations into the necessity of simplifying the PT structure and improving its potency–toxicity profiles. Such efforts resulted in the discovery of 4-azapodophyllotoxins (APT, **4**) (Fig. 1) [9], [10].

Lactone epimerisation is a particularly problematic feature of podophyllotoxin analogues. Epimerisation is a metabolic inactivation process, whereby the *trans* γ-lactone ring is converted to the *cis* isomer. Notably, picropodophyllin, the *cis* isomer of podophyllotoxin, is 100-fold less active [11]. In order to avoid the **C2** epimerization and/or the *trans* γ-lactone ring opening, a number of approaches have been proposed. These included the replacement of the *trans* γ-lactone with furan, thiolan or cyclopentane rings [9]. Another strategy described the preparation of derivatives substituted at the **C2** position [12], [13], [14]. In addition, the synthesis of analogues having a six-membered lactone ring was suggested because increasing the lactone ring size was assumed to give access to more stable isomers [15]. However to date none of these strategies has been successful in producing comparable activity to the *trans* γ-lactone compounds. The 4-azapodophyllotoxin (APT) scaffold was another alternative approach and during the last two decades there has been considerable effort towards its synthesis and antitumour evaluation. Classically, a three-component reaction is used to prepare 4-azapodophyllotoxin analogues (**4**) for its high synthetic efficiency and more importantly for reducing the number of stereocentres by removing the chirality at **C2** and **C3** and replacing them by a double bond [16].

The use of multicomponent reaction (MCR) approaches to prepare APT has allowed medicinal chemists to generate a diverse library of podophyllotoxin mimetics. The substantial advantages of these compounds include: retention of a comparable destabilisation of tubulin polymerisation as that of podophyllotoxin; considerable synthetic feasibility; provision of the opportunity to explore the effect of different structural modifications on antimitotic activity; and most importantly, a plausible solution for the problem of epimerisation that plagued podophyllotoxin and its stereochemically similar analogues, simply by removing the chirality at the stereocentres at **C2** and **C3** [9], [10]. Several APT derivatives were reported to be potent tumour inhibitors and vascular disrupting agents [17], [18].

In a recently published study, the potential antineoplastic activity of 1,10-phenanthroline was utilised to replace rings *A* and *B* of azapodophyllotoxin in order to combine the pharmacological properties of both these chromophores [19]. As a continuation of the efforts on structural modification of the azapodophyllotoxin scaffold (**4**), we were interested to further explore the impact of introducing different fluorinated moieties into ring *E*, on the activity of these compounds. Introduction of fluorinated substituents into drug candidates can provide unique protein-ligand interactions owing to the special combination of electronegativity, size and lipophilicity of fluorine atoms. These factors can have a substantial impact on molecular conformation, which in turn affect the binding affinity to the target protein and can also greatly affect physico-chemical and pharmaceutical properties [20].

## 2 Results and discussion

### 2.1 Ring E modifications of the 1,10 phenanthroline 4-azapodophyllotoxin analogues

Fluorination is often used in drug design to form novel interactions unavailable to the parent species due to the unique properties (size, electronegativity and lipophilicity) of the fluorine atom [20]. In an attempt to expand the range of possible ring *E* modifications in the [1], [10]-phenanthroline 4-azapodophyllotoxin scaffold [19], we explored the use of various fluorinated and non-fluorinated aromatic aldehydes (**7a-q**) in the multicomponent reaction (MCR) process.

**Scheme 1:**
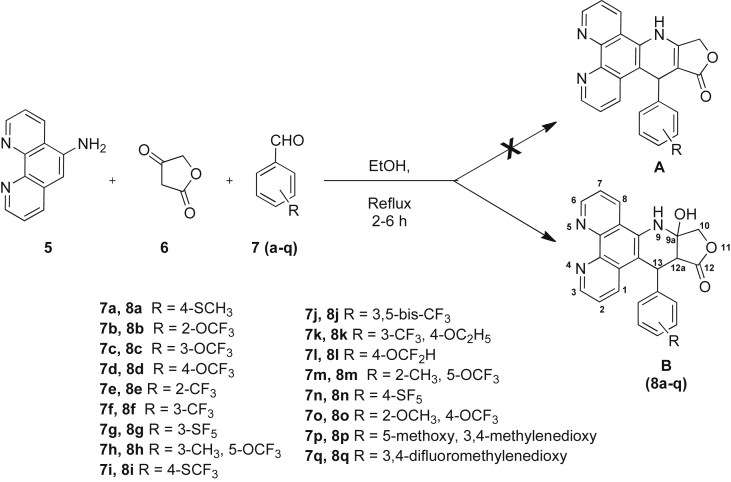
Synthesis of the unexpected **C3** hydroxy-4-azapodophyllotoxin compounds (**8a-q**).

We followed the reported azapodophyllotoxin procedure, *i.e*. one-pot, three-component reaction of 1,10 phenanthroline amine (**5**), tetronic acid (**6**) and different fluorinated and non-fluorinated aldehydes (**7a-q**) as shown in Scheme 1 [19]. To our surprise the spectroscopic data we generated did not agree with the expected previously reported formula A, Scheme 1. Further analysis of the spectroscopic data together with an X-ray crystal structure study (Section 2.2) led us to identify the unexpected and novel **C3** hydroxy (podophyllotoxin numbering system), *cis*-selective γ-lactone configuration of ring *C* (formula B), instead of the previously reported formula A (Scheme 1).

In our further ring *E* modification efforts, bulkier and more rigid substituents were introduced into ring *E* of compounds **8p** and **8q** in order to investigate their effect on the cytotoxic activity profile. The target compounds (**8a-q**) were prepared by reacting 1,10 phenanthroline amine (**5**), tetronic acid (**6**) and various fluorinated and non-fluorinated aldehydes (**7a-q**) in refluxing ethanol. In all cases the reaction proceeded smoothly and the isolated products were recrystallised from methanol to afford the pure compounds (**8a-q**).

**Scheme 2:**
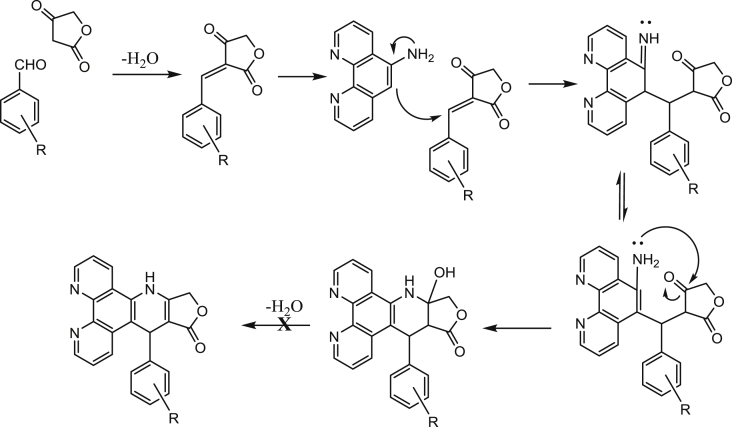
A possible mechanism of the formation of the unexpected 4-azapodophyllotoxin products (**8a-q**) Formula **B** rather than Formula **A.**

Spectroscopic analysis of our products revealed that the expected structure from the closely related analogues of the previous study *i.e.* formula A (Scheme 1) [19] was not entirely accurate but rather another unexpected structure represented by the general formula B (Scheme 1, Scheme 2) was obtained. The proton NMR spectra of all our products showed an extra hydrogen signal at around *δ* 3.50 (assigned to **CH12a** H) and another D_2_O exchangeable proton signal at around *δ* 6.00 (assigned to **CH9a** OH), combined with the presence of a tertiary carbon signal in the ^13^C NMR at around *δ* 50 (assigned to **CH12a**). The absence of a proton–proton coupling between **CH12a** and **CH13** was also instructive; both these CH signals appeared as singlets as would be expected from the Karplus equation for a dihedral torsional angle close to 90°. Moreover, mass spectrometry, elemental analysis and X-ray crystallography (*see* Section 2.2) confirmed the proposed structure of our compounds. These observations indicate that the reaction did not proceed to the final dehydrated form of formula A; instead it progressed to the hydrated product represented by formula B (Scheme 1, Scheme 2). Although the detailed mechanism of the reaction remains to be fully clarified, the formation of the unexpected hydrated products could be explained as shown in Scheme 2, which involves a sequence of condensation between tetronic acid **(6)** and the aromatic aldehydes **(7a-q)** to form the Knoevenagel adduct, followed by Michael addition of the 1,10 phenanthroline amine then cyclisation to afford the final products **(8a-q)**. In the literature there is one similar hydrated product of azapodophyllotoxin analogues reported in a microwave-assisted four-component reaction performed in ammonia/water, but no assignment of the chirality of ring *C* was mentioned [21].

### 2.2 Crystal structure analysis reveals the absolute configuration of the only two stereoisomers formed

**Fig. 2:**
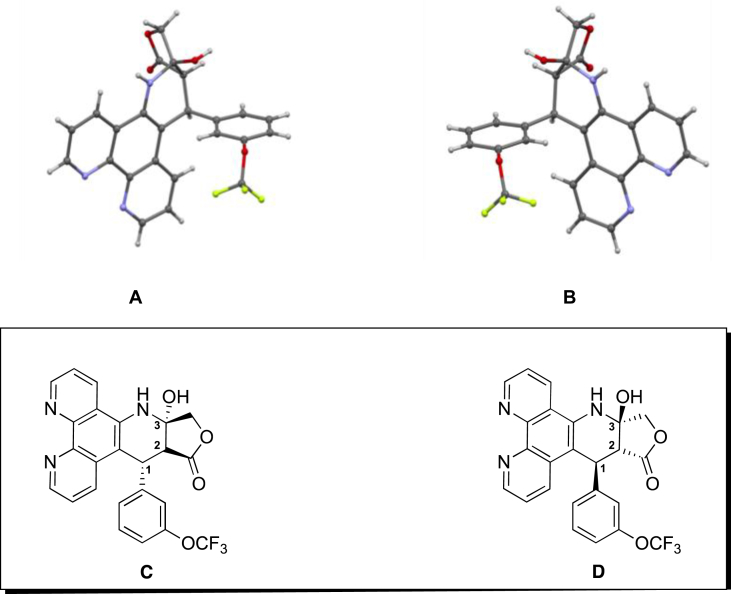
Crystal structure and absolute configuration of the two enantiomers of compound **8c**, (A) **C1-***S*, **C2**-*R* and **C3**-*R* antipode, (B) **C1-***R*, **C2**-*S* and **C3**-*S* antipode, (C) and (D) 2D representation of (A) and (B) respectively.

In order to gain more insight into the chirality of the three stereocentres (**C1**, **C2** and **C3** in Fig. 2) of our analogues, compound **8c** was selected for closer examination. Analysis of the X-ray crystal structure of **8c** established the relative configuration of our products (Fig. 2). Only two enantiomers were observed; each of which has a *trans* **C1 C2** and *cis* **C2 C3** lactone configuration. The stereoselectivity in forming the *cis* γ-lactone system can be attributed to the higher thermodynamic stability compared to the strained *trans* counterparts. This feature represents a particular metabolic advantage, as epimerisation would be unlikely to occur *in vivo*.

### 2.3 Cell growth inhibitory activity

**Fig. 3:**
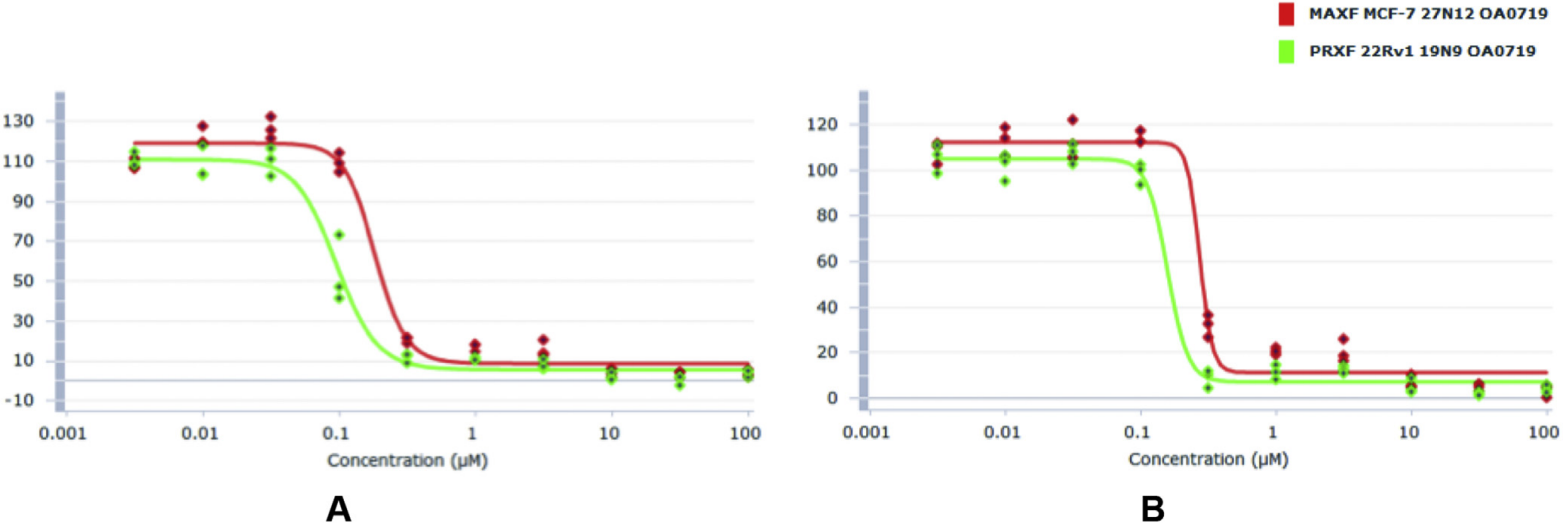
Cell viability dose response relationship of **8e** (A) and **8b** (B) in the Oncotest monolayer assay showing sigmoidal concentration-effect curves with low bottom plateaus indicating total cell kill at higher test concentration. The y-axis is representative of cell viability, as measured via the propidium iodide endpoint assay.

The anticancer activity of compounds (**8a-q**) was evaluated using the Oncotest monolayer assay in the human prostate cancer cell line 22Rv1 and the human breast cancer cell line MCF-7, as an outsourced service. Podophyllotoxin, etoposide and teniposide were used as positive controls. Antitumour activity was assessed after four days of treatment with the compounds using a propidium iodide based monolayer assay [22]. Potency is expressed as absolute IC_50_ values, calculated by non-linear regression analysis following testing in triplicate. The seventeen inhibitors were tested at 10 concentrations in half-log increments up to 100 μM in triplicate. The results summarised in Table 1 indicated that our compounds have significantly potent antiproliferative activity with IC_50_ values in the range of 0.11–7.25 μM, while the positive controls, etoposide and teniposide exhibited IC_50_ values in the range of 0.36–1.26 μM and 0.08–0.13 μM, respectively. Fourteen out of the seventeen tested compounds showed pronounced activity with IC_50_ values < 1 μM in both cell lines. These highly active inhibitors showed sigmoidal concentration-effect curves with low bottom plateaus indicating total cell kill at higher test concentration (Fig. 3). Overall, 22Rv1 appeared to be slightly more sensitive than MCF-7. The most active compound was shown to be **8e** with IC_50_ values of 0.11 μM (22Rv1) and 0.21 μM (MCF-7). Mean IC_50_ values < 0.5 μM were also detected for **8b**-**d**, **8g**, **8h**, **8j**, **8k** and **8o**, and mean IC_50_ values between 0.5 μM and 1.0 μM for **8a, 8f**, **8l**, **8n** and **8q**. Three further inhibitors (**8i**, **8m** and **8p**) exhibited mean IC_50_ values < 10 μM.

**Table 1:** *In vitro* antiproliferative activity (mean **IC**_**50**_ in **μM)** of compounds (**8a-q**) across two human cancer cell lines (MCF-7 and 22Rv1), following testing in triplicate.

Compound	Ar	MCF-7 IC_50_ (μM)	22Rv1 IC_50_ (μM)
**8a**		0.648	0.855
**8b**		0.288	0.165
**8c**		0.466	0.257
**8d**		0.424	0.239
**8e**		0.209	0.107
**8f**		0.825	0.510
**8g**		0.589	0.311
**8h**		0.475	0.242
**8i**		2.295	0.894
**8j**		0.643	0.380
**8k**		0.355	0.295
**8l**		0.901	0.719
**8m**		3.307	2.842
**8n**		0.857	0.590
**8o**		0.396	0.322
**8p**		7.250	4.002
**8q**		0.910	0.520
**Etoposide** **Teniposide** **Podophyllotoxin**		1.264 0.125 0.230	0.364 0.082 –

While the structural requirements of ring *E* in podophyllotoxin (PT) have not been extensively investigated because of the synthetic challenge in modifying the methoxy groups (**1**, Fig. 1), the data obtained in our series of compounds suggests that the methoxy groups of ring *E* are not essential for the activity and the best potency was observed with compounds having *ortho* substitution of ring *E*; 2-trifluoromethyl **8e**, 2-trifluoromethoxy **8b**, and 2-methoxy-4-trifluoromethoxy **8o**. Compounds with trifluoromethoxy substitution of ring *E* (**8b-d, 8h, 8o**) were among the most active compounds, and this preference remains whether the OCF_3_ is at the *ortho* (**8b**), *meta* (**8c**) or *para* (**8d**) position. Some compounds like **8b-8e, 8g, 8h, 8k, 8o** are more active than etoposide in both cell lines. Interestingly, ring *E* appeared to have tolerance towards a variety of fluorinated substituents, for example 3-pentafluorosulfanyl (**8g**), 3, 5-*bis*trifluoromethyl (**8j**), 4-pentafluorosulfanyl (**8n**), and difluoro-methylenedioxy analogues (**8q**) have submicromolar activity. However, the myristicin-derived substitution of ring *E* in compound (**8p**) exhibited some reduction in the antiproliferative activity (IC_50_ 7.3 μM and 4.0 μM in breast MCF7 and prostate 22Rv1 cancer cell lines, respectively). Fig. 3 shows typical dose–response curves for two of the most active compounds (**8e** and **8b**) across the ten concentration values used in the assay to calculate IC_50_ values.

### 2.4 Docking studies reveals a structural basis for the anti-tubulin activity

**Fig. 4:**
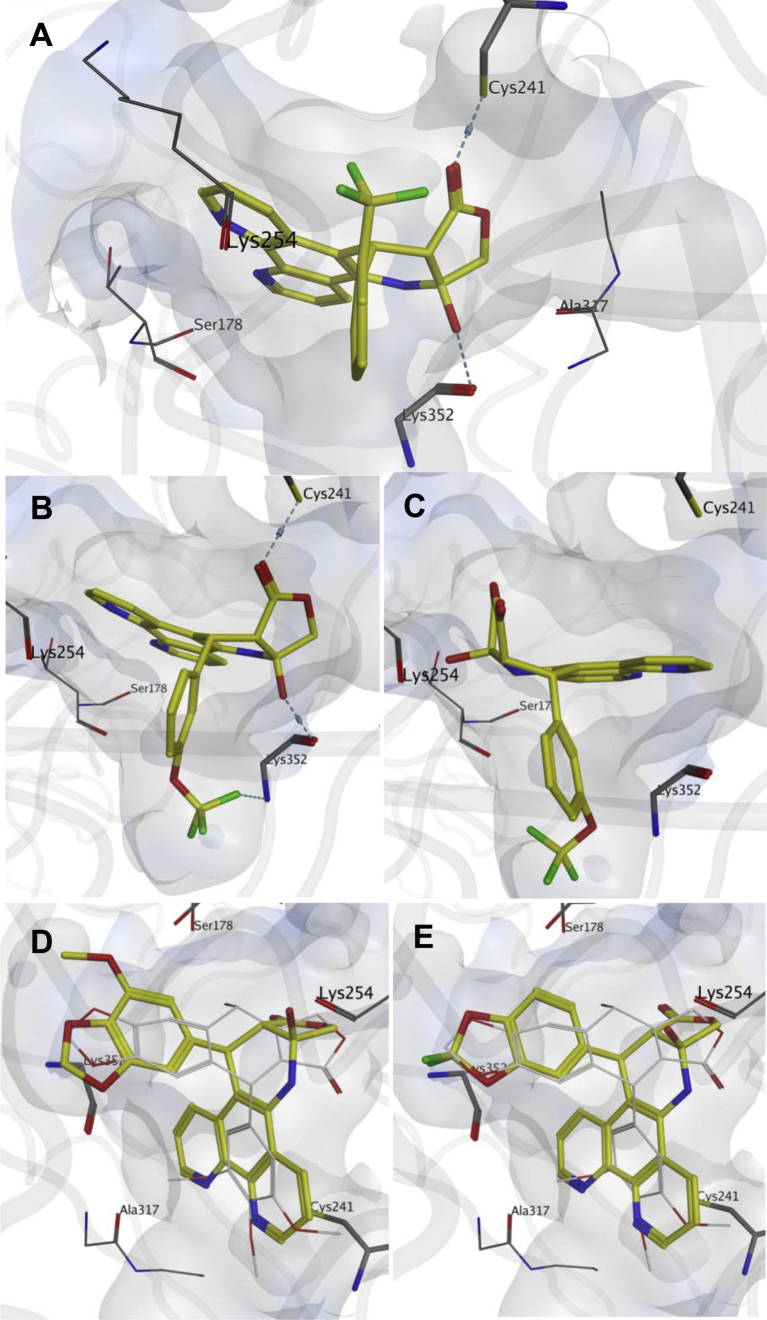
The predicted binding modes of compound **8e**, **C1**-*R*-enantiomer (**A**); and compound **8c**; **C1**-*R*-enantiomer (**B**), both showing the cross linking hydrogen bonds formed between Cys 241 and Lys 352 amino acids (blue dashed lines) compared to the flipped orientation of **8c**, **C1**–*S*-enantiomer (**C**). The predicted binding modes of compounds **8p**; **C1**-*R*-enantiomer **(D)** and **8q**; **C1**-*R*-enantiomer **(E)**, both overlaid with the co-crystallised podophyllotoxin molecule (in white). (For interpretation of the references to colour in this figure legend, the reader is referred to the web version of this article.)

Computational docking simulations were performed to explore the binding modes of the **C3** hydroxy *cis* γ-lactone APT analogues (**8a-q**). All the synthesised compounds were docked into the colchicine binding site of the human tubulin-podophyllotoxin crystal structure (PDB ID: 1SA1) [23], [24], [25]. The *in silico* modelling studies led to two major observations; first, the stereochemistry at carbon **C1** (podophyllotoxin numbering system) is critical to the ability of these compounds to fit satisfactorily inside the tubulin binding pocket. The **C1**-*R*-enantiomers of these compounds resulted in better binding mode than those of the **C1**-*S*-enantiomers. Interestingly, this is the same absolute configuration of **C1** of podophyllotoxin. Second, our model predicts that the best fitting compounds are forming a particular stereochemical architecture made of the **C3** hydroxy group on one side of the molecule and the *cis* lactone ring on the opposite side. This configuration enables these inhibitors to form a hydrogen bond bridge between the carbonyl C=O group of the *cis* lactone ring and the side chain thiol SH group of βCys241 on one side of the molecule, and between the **C3** hydroxy group OH and the backbone carbonyl C=O group of βLys352 on the opposite side of the molecule. Fig. 4A depicts the best docking fit of compound **8e**, **C1**-*R*-enantiomer. This novel mode of interaction may explain why these compounds are able to retain remarkable antitubulin activity in spite of having the notoriously unfavourable *cis* lactone system [26]. It is also worth mentioning that according to our docking study this type of cross linking interaction is only exhibited by **C1**-*R*-enantiomers, the **C1**-*S* counterparts are not able to occupy the proper orientation required for this binding mode. Instead, they display a “flipped” conformation in the binding site, as shown in Fig. 4B *vs.* 4C, which depicts compound **8c**, (**C1**-*R*-enantiomer Fig. 4B) in contrast to the flipped orientation (**C1**-*S*-enantiomer Fig. 4C). Interestingly, our system predicts a hydrogen bond formation between the *OCF*_*3*_ moiety in compound **8c** and the backbone NH group of βLys352 (Fig. 4B). Notably, most ring *E* substitution patterns docked well in the binding site and showed little difference in the docking scores. This could be attributed to the predominance of the hydrophobic interactions in the site occupied by this ring, which allow for promiscuous substituents on the *E* ring. This observation is in accordance with the slight fluctuation in the potencies of most of these compounds in the cell viability tests (Table 1). In the case of compounds **8p** and **8q**, the relatively bulky structure of ring *E* would clash with the protein and prevent them from occupying the above mentioned binding mode which features the simultaneous interaction with βCys241 and βLys352, instead they nicely overlay with podophyllotoxin where the tricyclic 1,10 phenanthroline ring maps well with ring *E* of podophyllotoxin while the methylenedioxy ring in both compounds overlap with rings *A* and *B* of the natural product Fig. 4D and E. Also, the relative reduction in the activity of compound **8p** could be attributed to the size of the additional methoxy group in ring *E,* which is likely to cause clashes within the protein binding site (Fig. 4D).

### 2.5 Analogues **8e** and **8b** induce disruption of the microtubule cytoskeleton in MCF-7 breast cancer cells

**Fig. 5:**
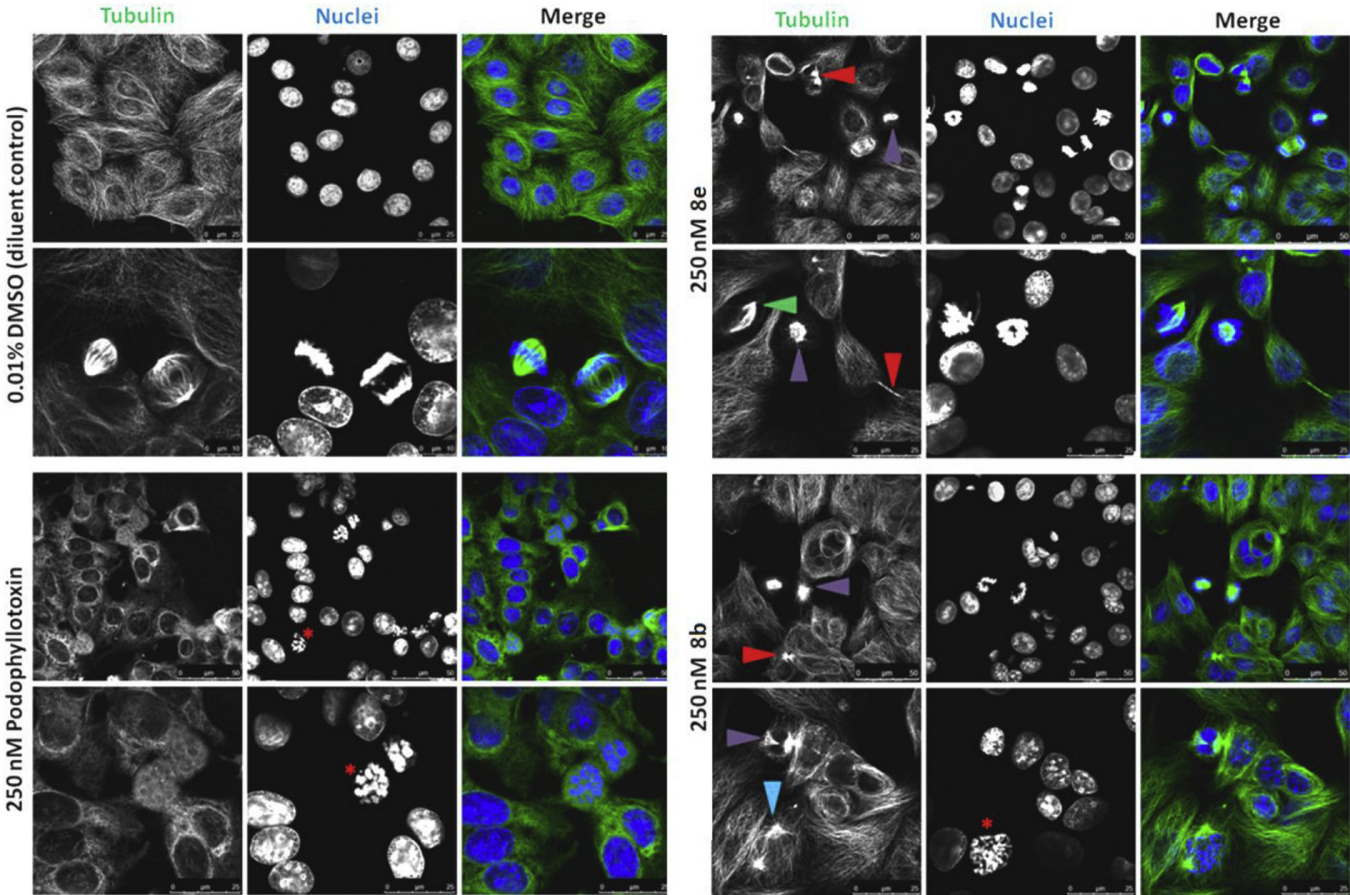
Microtubule deregulation in MCF-7 cells induced by podophyllotoxin and analogues **8e** and **8b**. MCF-7 cells were treated for 6 h with 250 nM of the compounds (or the equivalent concentration of DMSO diluent control) as indicated. Cells were fixed with 100% methanol at −20 °C for 10 min and subjected to immunofluorescence analysis for α-tubulin (green channel). Nuclei were counterstained with Hoechst (blue channel). Cells were imaged by confocal microscopy and representative images from three independent experiments are shown. Cell defects are indicated as follows: pyknotic nucleus (red asterisk), intracellular bridge forming a binucleate cell (red arrowhead), aberrant spindle morphology (purple arrowhead), asymmetric spindle formation (green arrowhead), loss of spindle polarity (blue arrowhead). (For interpretation of the references to colour in this figure legend, the reader is referred to the web version of this article.)

In order to investigate the microtubule-destabilising ability of these analogues, experiments were conducted to determine their effects on the subcellular organisation of microtubules *in vitro* [27]. For this we studied the most active compounds **8e** and **8b** using immunofluorescence assays and confocal microscopy. These analyses of α-tubulin in treated versus control MCF-7 cells (Fig. 5**)** revealed that our 4-azapodophyllotoxin analogues possessed microtubule deregulating activity with evidence of enhanced specificity for dividing cells compared with podophyllotoxin. Diluent control treated cells in interphase exhibited typical nest-like microtubule networks comprised of long, regularly arranged filaments (Fig. 5): 0.01% DMSO (top row). The nuclei of control interphase cells were relatively monomorphic. Dividing control cells demonstrated classic hallmarks of different phases of mitosis e.g. congressed chromosomes, and the formation of symmetrical, bipolar spindles (Fig. 5): 0.01% DMSO (bottom row). All podophyllotoxin treated cells showed a dramatic disruption of the tubulin cytoskeleton: there was a significant loss of filamentous structures and evidence of nuclear atypia and pyknosis. Analogue treated cells in interphase featured shorter microtubule filaments and slightly more disordered microtubule networks than control cells. However, compared with podophyllotoxin, **8e** and **8b** induced more subtle changes in the tubulin filaments and their organisation. Deregulation of the tubulin cytoskeleton by our analogues was most striking in dividing cells, which displayed a variety of microtubule and nuclear abnormalities. Compound **8e** and **8b**-induced defects included aberrations of spindle morphology and polarity, nuclear atypia (pleomorphism, pyknosis) and uncongressed/misaligned chromosomes. Impairment of cytokinesis was demonstrated by an accumulation of binucleate cells joined by intracellular bridges (red arrowheads in Fig. 5).

### 2.6 Analogues **8e** and **8b** do not impair microtubule polymerisation in MCF-7 breast cancer cells

**Fig. 6:**
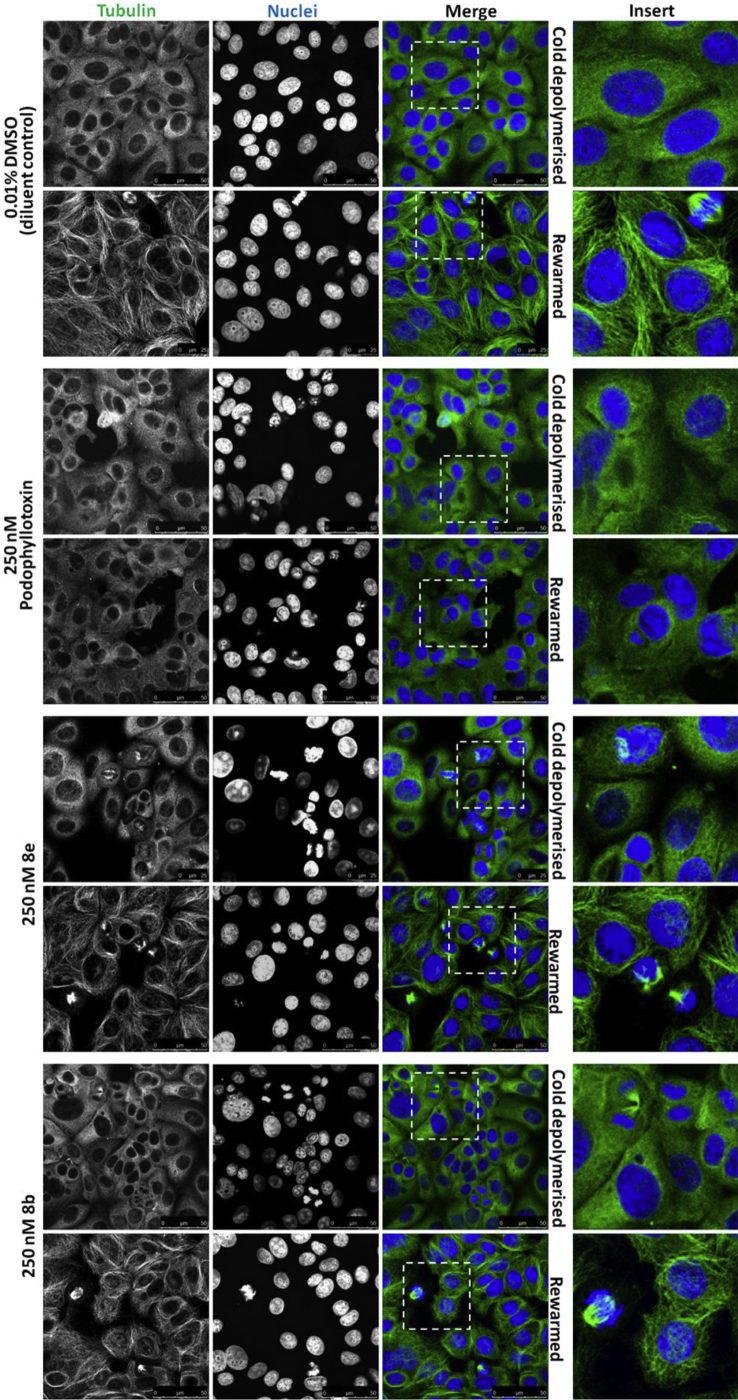
Microtubule repolymerisation in cold-depolymerised MCF-7 cells following 6 h pre-treatment with podophyllotoxin and analogues **8e** or **8b**. MCF-7 cells were treated for 6 h with 250 nM of the compounds (or the equivalent concentration of DMSO diluent control) as indicated. Cells were incubated on ice for 30 min, some cells were then fixed with 100% methanol at −20 °C for 10 min (top rows) while some were rewarmed at 37 °C for 30 min prior to fixation (bottom rows). Fixed cells were subjected to immunofluorescence for α-tubulin (green channel). Nuclei were counterstained with Hoechst (blue channel). Cells were imaged by confocal microscopy and representative images from three independent experiments are shown. (For interpretation of the references to colour in this figure legend, the reader is referred to the web version of this article.)

**Fig. 7:**
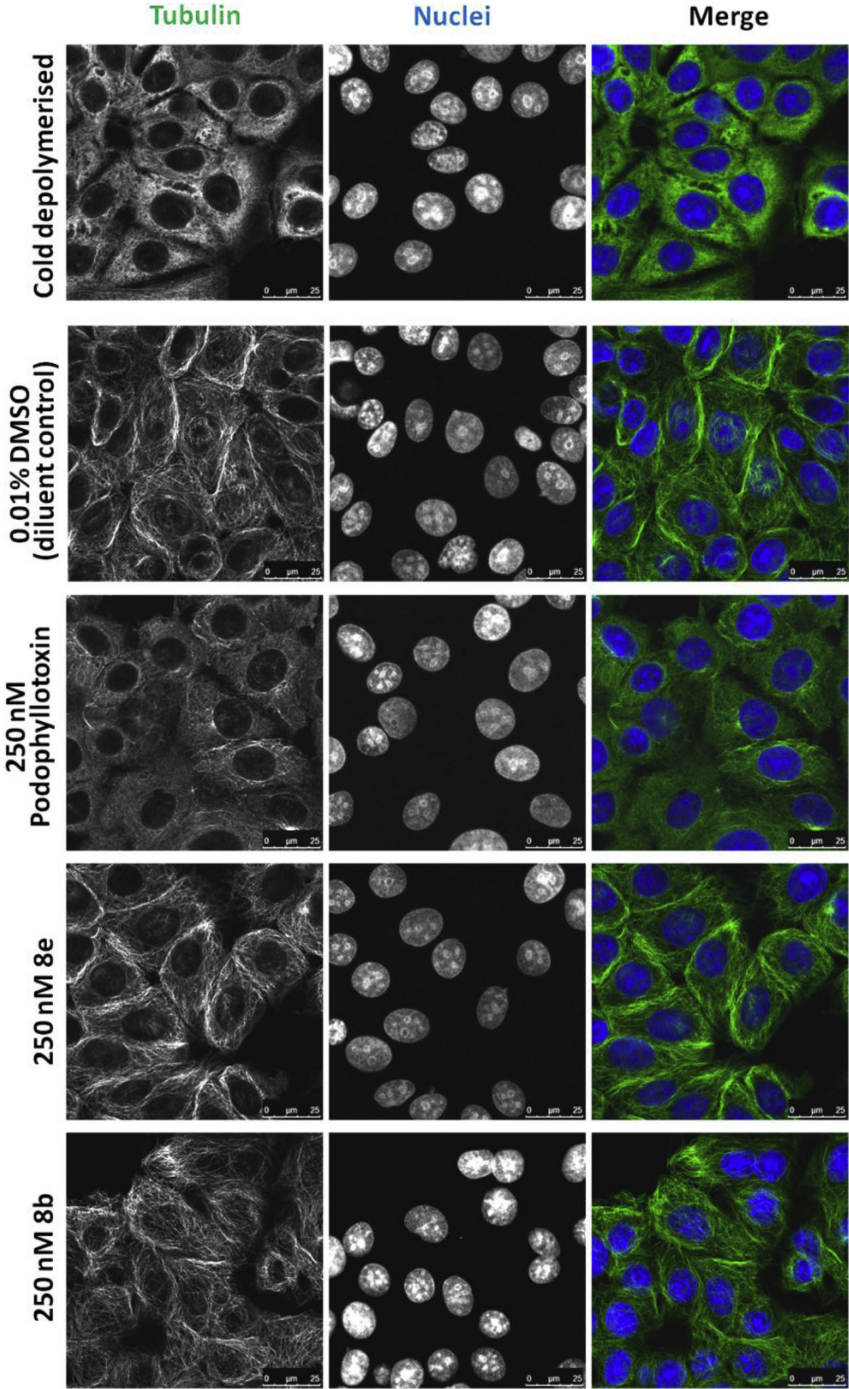
Microtubule repolymerisation in the presence of podophyllotoxin, analogues **8e** or **8b** following cold-depolymerisation. MCF-7 cells were incubated on ice for 30 min, a sample of “cold depolymerised” cells were then fixed with 100% methanol at −20 °C for 10 min (top row). The remaining cells were fixed after being incubated for 30 min at 37 °C with 250 nM of the compounds (or the equivalent concentration of DMSO) as indicated. Fixed cells were subjected to immunofluorescence for α-tubulin (green channel). Nuclei were counterstained with Hoechst (blue channel). Cells were imaged by confocal microscopy and representative images from three independent experiments are shown. (For interpretation of the references to colour in this figure legend, the reader is referred to the web version of this article.)

Cold depolymerisation of the tubulin cytoskeleton *in vitro*, including in MCF-7 cells, can be achieved by relatively short incubations at 4 °C [27], [28], [29]. To test the ability of the compounds to impair microtubule polymerisation, following depolymerisation, two variations of a tubulin re-polymerisation assay were performed. In the first, MCF-7 cells were pre-treated for 6 h with 250 nM of the compounds before being incubated on ice for 30 min then rewarmed to 37 °C for a further 30 min. In the second variation, cells were first incubated on ice then rewarmed to 37 °C in the presence of the compounds (250 nM, 30 min total treatment time). Samples of cells were fixed post-cold depolymerisation and post-warm repolymerisation. In both variations, immunofluorescence for α-tubulin was performed and cells were imaged by confocal microscopy (Fig. 6 and **7**). In control and treated cells, incubation on ice for 30 min led to total loss of filamentous tubulin and the protein was diffusely scattered throughout the cytoplasm. The compounds therefore did not inhibit depolymerisation of the microtubules. Upon increasing the temperature to 37 °C for 30 min there was clear repolymerisation of the microtubule network. Both assay variations demonstrated that the podophyllotoxin positive controls significantly impaired the repolymerisation of tubulin following cold depolymerisation (Fig. 6, Fig. 7**)**. In the cells pre-treated for 6 h with **8e** or **8b** (Fig. 6), the microtubule cytoskeleton was still able to reform but mitotic defects and shorter filaments, as seen in Fig. 5, were evident in interphase cells. After a 30 min rewarming incubation in the presence of these analogues the microtubule cytoskeleton was able to reform with negligible defects (Fig. 7).

The anti-cancer activity of tubulin binding drugs such as paclitaxel and vincristine is a consequence of their ability to deregulate spindle microtubule dynamics; inducing mitotic arrest and apoptosis [30]. The mitotic defects generated in MCF-7 breast cancer cells by **8e** and **8b** treatment (Fig. 5), and the more modest effects on tubulin morphology and polymerisation in interphase cells (Fig. 5, Fig. 6, Fig. 7), suggest a degree of selectivity for dividing cells. Such selectivity is a key feature of many successful anticancer compounds and taken together with the viability data shown in Table 1, the results suggest that these analogues have chemotherapeutic potential through the suppression of spindle microtubule dynamics.

## 3 Conclusions

A series of 4-azapodophyllotoxin derivatives were synthesised using a multicomponent reaction (MCR) of 1,10 phenanthroline amine **(5)**, tetronic acid **(6)** and various fluorinated and non-fluorinated aromatic aldehydes **(7a-q)**. To our surprise we obtained the unexpected **C3** hydroxy *cis* γ-lactone derivatives (**8a-q**) and proved unequivocally that the previously reported structure is not accurate. Moreover, the reaction proceeded in favour of the formation of only two thermodynamically stable *cis* γ lactone enantiomers as confirmed by the X-ray crystal structure studies. This finding is of particular interest in terms of the biochemical mechanism by which podophyllotoxin compounds are metabolised where the release of the highly strained *trans* γ-lactone system in podophyllotoxin analogues into the more stable **C3** hydroxy *cis* lactone motif and yet retaining a significantly potent activity should allow for improved pharmacokinetic properties. These compounds were evaluated against two human cancer lines MCF-7 and 22Rv1. Fourteen out of the seventeen tested compounds exhibited sub-micromolar activity with IC_50_ values in the range of 0.11–0.91 μM. Confocal microscopy studies confirmed that analogues **8b** and **8e** had significant effects on microtubular morphology that was different to PT, potentially with greater selectivity for dividing cells. Molecular modelling simulations predicted a theoretical basis for our findings.

Overall, this study provides, for the first time, the *cis* selective **C3** hydroxy γ-lactone configuration into podophyllotoxin-mimetic libraries and lays a foundation for the development of alternative tubulin targeting therapies capable of combating cancer.

## 4 Experimental section

### 4.1 Chemistry

All chemicals were purchased from Sigma–Aldrich or Alfa Aesar and were used without further purification. Thin Layer Chromatography (TLC): precoated aluminium backed plates (60 F_254_, 0.2 mm thickness, Merck) were visualized under both short and long wave UV light (254 and 366 nm). Flash column chromatography was carried out using silica gel supplied by Fisher (60A, 35–70 μm). Analytical High Performance Liquid Chromatography (HPLC) analysis was performed using either a ThermoScientific or a Varian Prostar system. ^1^H NMR (500 MHz), ^13^C NMR (125 MHz) and ^19^F NMR (470 MHz) spectra were recorded on a Bruker Avance 500 MHz spectrometer at 25 °C. Chemical shifts (*δ*) are expressed in parts per million (ppm) and coupling constants (J) are given in hertz. The following abbreviations are used in the assignment of NMR signals: s (singlet), bs (broad singlet), d (doublet), t (triplet), q (quartet), qn (quintet), m (multiplet), dd (doublet of doublet), dt (doublet of triplet), td (triple doublet), dq (double quartet), m (multiplet), dm (double multiplet). Mass spectrometry was run on a Bruker Micromass system in electrospray ionisation mode. Accurate mass spectrometry was performed at the EPSRC UK National Mass Spectrometry facility at Swansea University. Elemental analysis (% C, H, N) was run at Medac Ltd. (Chobham, Surrey, U.K.) as an external service.

#### 4.1.1 General synthesis of 4-aza-3-hydroxy-1,2-trans-2,3-cis-lactone podophyllotoxin derivatives

These derivatives were synthesised by following a previously reported method [13]. An equimolar mixture of commercially available tetronic acid, 1,10 phenanthroline amine and the corresponding aromatic aldehyde was dissolved in ethanol. The reaction mixture was heated under reflux for 2–6 h. After cooling, the solvent was removed *in vacuo* and the product was recrystallised from methanol to afford the desired compounds in pure form.

##### 4.1.1.1 (9aS, 12aR, 13R) and (9aR, 12aS, 13S) 9a-hydroxy-13-(4-(methylthio)phenyl)-9a,10,12a,13-tetrahydrofuro[3,4-b]pyrido[3,2-f][1,10]phenanthrolin-12(9H)-one (8a)

Yield: 76 ^1^H NMR (500 MHz, DMSO-d_6_) *δ* 9.11 (dd, J = 1.5, 4.5 Hz, 1H, Ar*H*), 8.91 (dd, J = 1.5, 8.5 Hz, 1H, Ar*H*), 8.72 (d, J = 1.5, 4 Hz, 1H, Ar*H*), 7.93 (dd, J = 1.5, 9 Hz, 1H, Ar*H*), 7.85 (dd, J = 4.5, 8.5 Hz, 1H, Ar*H*), 7.51 (s, 1H, N*H*-9), 7.48 (dd, J = 4, 8.5 Hz, 1H, Ar*H*), 7.22 (d, J = 8.5 Hz, 2H, Ar*H*), 7.11 (d, J = 8.5 Hz, 2H, Ar*H*), 6.05 (s, 1H, O*H*), 4.98 (s, 1H, C*H*-13), 4.57 (d, J = 8.5 Hz, 1H, C*H-*10), 4.21 (d, J = 8.5 Hz, 1H, C*H-*10), 3.55 (s, 1H, C*H-*12a), 2.41 (s, 3H, C*H*_3_). ^13^C NMR (125 MHz, DMSO-d_6_) *δ* 174.59 (*C*=O), 149.59 (Ar*CH)*, 145.75 (Ar*C*), 145.18 (Ar*CH)*, 141.14 (Ar*C*), 139.10 (Ar*C*), 135.79 (Ar*C*), 135.40 (Ar*C*), 130.01 (Ar*CH)*, 129.87 (Ar*CH)*, 129.05 (Ar*C*H-*3′,5’*), 128.43 (Ar*C*), 125.77 (Ar*C*H), 123.27 (Ar*CH)*, 122.59 (Ar*CH)*, 120.56 (Ar*C*), 104.12 (Ar*C*), 81.89 (*C*OH*-*9a), 74.49 (*C*H_2_*-*10), 50.09 (*C*H-12a), 35.94 (*C*H-13), 14.56 (S*C*H_3_). HRMS calcd for C_24_H_19_N_3_O_3_S (M + H)+, 430.1220; found, 430.1217.

##### 4.1.1.2 (9aS, 12aS, 13S) and (9aR, 12aR, 13R) 9a-hydroxy-13-(2-(trifluoromethoxy)phenyl)-9a,10,12a,13-tetrahydrofuro[3,4-b]pyrido[3,2-f][1,10]phenanthrolin-12(9H)-one (8b)

Yield: 72% ^1^H NMR (500 MHz, DMSO-d_6_) *δ* 9.18 (dd, J = 1.5, 4 Hz, 1H, Ar*H*), 8.98 (dd, J = 1, 8.5 Hz, 1H, Ar*H*), 8.78 (d, J = 1.5, 4.5 Hz, 1H, Ar*H*), 7.91 (dd, J = 4, 8.5 Hz, 1H, Ar*H*), 7.67 (dd, J = 1.5, 8.5 Hz, 1H, Ar*H*), 7.63 (s, 1H, N*H*-9), 7.57–7.51 (m, 2H, Ar*H*), 7.40 (ddd, J = 2, 7.5, 9 Hz, 1H, Ar*H*), 7.13 (td, J = 1, 8 Hz, 1H, Ar*H*), 7.02 (dd, J = 1, 8 Hz, 1H, Ar*H*), 6.19 (s, 1H, O*H*-9a), 5.29 (s, 1H, C*H*-13), 4.65 (d, J = 8.5 Hz, 1H, C*H-*10), 4.33 (d, J = 8.5 Hz, 1H, C*H-*10), 3.50 (s, 1H, C*H-*12a); ^19^F NMR (DMSO-d_6_) *δ* −55.36; ^13^C NMR (125 MHz, DMSO-d_6_) *δ* 173.97 (*C*=O), 149.86 (Ar*CH*), 146.10 (Ar*C*), 145.90 (Ar*C*), 145.35 (Ar*CH*), 141.33 (Ar*C*), 136.30 (Ar*C*), 133.81 (Ar*C*), 131.11 (Ar*CH)*, 130.07 (Ar*CH*), 128.78 (Ar*C*H), 128.42 (Ar*C*H), 128.08 (Ar*C*), 127.03 (Ar*C*H), 123.42 (Ar*CH*), 122.70 (Ar*CH*), 120.93 (q, ^*1*^*J*_*C-F*_ = 256 Hz, *CF*_*3*_), 120.55 (Ar*C*), 120.18 (Ar*C*H), 102.43 (Ar*C*), 81.65 (*C*OH*-*9a), 74.54 (*C*H_2_*-*10), 48.35 (*C*H-12a), 30.37 (*C*H-13). HRMS calcd for C_24_H_16_F_3_N_3_O_4_ (M + H)+, 468.1166; found, 468.1158.

##### 4.1.1.3 (9aS, 12aR, 13R) and (9aR, 12aS, 13S) 9a-hydroxy-13-(3-(trifluoromethoxy)phenyl)-9a,10,12a,13-tetrahydrofuro[3,4-b]pyrido[3,2-f][1,10]phenanthrolin-12(9H)-one (8c)

Yield: 74% ^1^H NMR (500 MHz, DMSO-d_6_) *δ* 9.12 (dd, J = 1.5, 4.5 Hz, 1H, Ar*H*), 8.93 (dd, J = 1.5, 8.5 Hz, 1H, Ar*H*), 8.72 (dd, J = 1.5, 4 Hz, 1H, Ar*H*), 7.94 (dd, J = 1.5, 8.5 Hz, 1H, Ar*H*), 7.86 (dd, J = 4.5, 8.5 Hz, 1H, Ar*H*), 7.56 (s, 1H, N*H*-9), 7.48 (dd, J = 4.5, 8.5 Hz, 1H, Ar*H*), 7.40–7.33 (m, 2H, Ar*H*), 7.25 (s, 1H, Ar*H*), 7.19–7.14 (m, 1H, Ar*H*), 6.16 (s, 1H, O*H*-9a), 5.10 (s, 1H, C*H*-13), 4.58 (d, J = 8.5 Hz, 1H, C*H-*10), 4.23 (d, J = 8.5 Hz, 1H, C*H-*10), 3.63 (s, 1H, C*H-*12a); ^19^F NMR (DMSO-d_6_) *δ* −56.62; ^13^C NMR (125 MHz, DMSO-d_6_) *δ* 174.31 (*C*=O), 149.75 (Ar*CH*), 148.23 (Ar*C*), 145.85 (Ar*C*), 145.32 (Ar*CH*), 145.21 (Ar*C*), 141.17 (Ar*C*), 135.59 (Ar*C*), 130.08 (Ar*CH*), 130.03 (Ar*CH*), 129.70 (Ar*C*H), 128.28 (Ar*C*), 127.67 (Ar*C*H), 123.31 (Ar*C*H), 122.64 (Ar*C*H), 121.14 (Ar*CH*), 120.98 (q, ^*1*^*J*_*C-F*_ = 234 Hz, *CF*_*3*_), 120.54 (Ar*C*), 118.79 (Ar*C*H), 103.51 (Ar*C*), 81.84 (*C*OH*-*9a), 74.45 (*C*H_2_*-*10), 49.81 (*C*H-12a), 36.04 (*C*H-13). HRMS calcd for C_24_H_16_F_3_N_3_O_4_ (M + H)+, 468.1166; found, 468.1160.

##### 4.1.1.4 (9aS, 12aR, 13R) and (9aR, 12aS, 13S) 9a-hydroxy-13-(4-(trifluoromethoxy)phenyl)-9a,10,12a,13-tetrahydrofuro[3,4-b]pyrido[3,2-f][1,10]phenanthrolin-12(9H)-one (8d)

Yield: 70% ^1^H NMR (500 MHz, DMSO-d_6_) *δ* 9.12 (dd, J = 1.5, 4 Hz, 1H, Ar*H*), 8.92 (dd, J = 1.5, 8.5 Hz, 1H, Ar*H*), 8.73 (dd, J = 1.5, 4 Hz, 1H, Ar*H*), 7.93 (dd, J = 1.5, 8.5 Hz, 1H, Ar*H*), 7.85 (dd, J = 4, 8.5 Hz, 1H, Ar*H*), 7.52 (s, 1H, N*H*-9), 7.48 (dd, J = 4, 8 Hz, 1H, Ar*H*), 7.41 (d, J = 8.5, 2H, Ar*H*), 7.21 (d, J = 8.5, 2H, Ar*H*), 6.08 (s, 1H, O*H*-9a), 5.09 (s, 1H, C*H*-13), 4.59 (d, J = 8.5 Hz, 1H, C*H-*10), 4.23 (d, J = 8.5 Hz, 1H, C*H-*10), 3.62 (s, 1H, C*H-*12a); ^19^F NMR (DMSO-d_6_) *δ* −56.75; ^13^C NMR (125 MHz, DMSO-d_6_) *δ* 174.36 (*C*=O), 149.69 (Ar*CH*), 146.81 (Ar*C*), 145.87 (Ar*C*), 145.28 (Ar*CH*), 141.65 (Ar*C*), 141.25 (Ar*C*), 135.51 (Ar*C*), 130.36 (Ar*C*H), 130.02 (Ar*C*H), 129.68 (Ar*C*H), 128.32 (Ar*C*), 123.32 (Ar*C*H), 122.59 (Ar*C*H), 120.67 (Ar*C*H), 120.56 (Ar*C*), 120.52 (q, ^*1*^*J*_*C-F*_ = 253.9 Hz, CF_3_), 103.81 (Ar*C*), 81.92 (*C*OH*-*9a), 74.55 (*C*H_2_*-*10), 49.94 (*C*H-12a), 35.76 (*C*H-13). HRMS calcd for C_24_H_16_F_3_N_3_O_4_ (M + H)+, 468.1166; found, 468.1158.

##### 4.1.1.5 (9aS, 12aR, 13R) and (9aR, 12aS, 13S) 9a-hydroxy-13-(2-(trifluoromethyl) phenyl)-9a,10,12a,13-tetrahydrofuro[3,4-b]pyrido[3,2-f][1,10]phenanthrolin-12(9H)-one (8e)

Yield: 68% ^1^H NMR (500 MHz, DMSO-d_6_) *δ* 9.17 (dd, J = 1.5, 4.5 Hz, 1H, Ar*H*), 9.00 (dd, J = 1.5, 8.5 Hz, 1H, Ar*H*), 8.75 (dd, J = 1.5, 4.5 Hz, 1H, Ar*H*), 7.91 (dd, J = 4.5, 8.5 Hz, 1H, Ar*H*), 7.89 (dd, *J* = 1, 8 Hz, 1H, Ar*H*), 7.78 (dd, J = 1.5, 5 Hz, 1H, Ar*H*), 7.67 (s, 1H, N*H*-9), 7.52 (dd, J = 4.5, 8.5 Hz, 1H, Ar*H*), 7.46 (t, J = 7.5 Hz, 1H, Ar*H*), 7.39 (t, J = 7.5 Hz, 1H, Ar*H*), 7.28 (d, J = 7.5 Hz, 1H, Ar*H*), 6.19 (s, 1H, O*H*-9a), 5.31 (s, 1H, C*H*-13), 4.65 (d, J = 8.5 Hz, 1H, C*H-*10), 4.30 (d, J = 8.5 Hz, 1H, C*H-*10), 3.53 (s, 1H, C*H-*12a); ^19^F NMR (DMSO-d6) *δ* −57.66; ^13^C NMR (125 MHz, DMSO-d_6_) *δ* 173.79 (*C*=O), 149.91 (Ar*CH*), 145.91 (Ar*C*), 145.32 (Ar*C*H), 141.40 (Ar*C*), 140.51 (Ar*C*), 136.45 (Ar*C*), 132.30 (Ar*C*H), 131.62 (Ar*C*H), 130.15 (Ar*CH*), 128.43 (Ar*C*H), 128.11 (Ar*C*), 127.51 (Ar*C*H), 126.32 (q, ^*3*^*J*_*C-F*_ = 5.9 Hz, Ar*C*H), 126.54 (q, ^*2*^*J*_*C-F*_ = 29 Hz, Ar*C*), 125.45 (q, ^*1*^*J*_*C-F*_ = 272.5 Hz, *CF*_*3*_), 123.37 (Ar*C*H), 122.75 (Ar*C*H), 120.55 (Ar*C*), 103.49 (Ar*C*), 81.61 (*C*OH*-*9a), 74.41 (*C*H_2_*-*10), 49.12 (*C*H-12a), 33.25 (*C*H-13). HRMS calcd for C_24_H_16_F_3_N_3_O_3_ (M + H)+, 452.1217; found, 452.1211.

##### 4.1.1.6 (9aS, 12aR, 13R) and (9aR, 12aS, 13S) 9a-hydroxy-13-(3-(trifluoromethyl) phenyl)-9a,10,12a,13-tetrahydrofuro[3,4-b]pyrido[3,2-f][1,10]phenanthrolin-12(9H)-one (8f)

Yield: 71% ^1^H NMR (500 MHz, DMSO-d_6_) *δ* 9.13 (dd, J = 1, 4 Hz, 1H, Ar*H*), 8.93 (dd, J = 1, 8.5 Hz, 1H, Ar*H*), 8.73 (dd, J = 1.5, 4 Hz, 1H, Ar*H*), 7.94 (dd, J = 1.5, 9 Hz, 1H, Ar*H*), 7.86 (dd, *J* = 4.5, 8.5 Hz, 1H, Ar*H*), 7.73 (s, 1H, N*H*-9), 7.57–7.50 (m, 3H, Ar*H*), 7.48 (dd, J = 4, 8.5 Hz, 1H, Ar*H*), 7.44 (t, J = 8 Hz, 1H, Ar*H*), 6.13 (s, 1H, O*H*-9a), 5.16 (s, 1H, C*H*-13), 4.59 (d, J = 8.5 Hz, 1H, C*H-*10), 4.23 (d, J = 9 Hz, 1H, C*H-*10), 3.65 (s, 1H, C*H-*12a); ^19^F NMR (DMSO-d_6_) *δ* −60.82; ^13^C NMR (125 MHz, DMSO-d_6_) *δ* 174.22 (*C*=O), 149.75 (Ar*CH*), 145.88 (Ar*C*), 145.33 (Ar*C*H), 143.66 (Ar*C*), 141.21 (Ar*C*), 135.69 (Ar*C*), 132.69 (Ar*C*H), 130.08 (Ar*C*H), 129.69 (Ar*CH*), 129.15 (Ar*C*H), 129.42 (q, ^*2*^*J*_*C-F*_ = 31.6 Hz, Ar*C*), 128.27 (Ar*C*), 125.60 (q, ^*3*^*J*_*C-F*_ = 3.9 Hz, Ar*C*H), 124.19 (q, ^*1*^*J*_*C-F*_ = 267.6 Hz, *C*F_3_), 123.34 (Ar*C*H), 123.29 (q, ^*3*^*J*_*C-F*_ = 3.9 Hz, Ar*C*H), 122.63 (Ar*C*H), 120.55 (Ar*C*), 103.43 (Ar*C*), 81.89 (*C*OH*-*9a), 74.52 (*C*H_2_*-*10), 49.90 (*C*H-12a), 36.09 (*C*H-13). HRMS calcd for C_24_H_16_F_3_N_3_O_3_ (M + H)+, 452.1217; found, 452.1211.

##### 4.1.1.7 (9aS, 12aR, 13R) and (9aR, 12aS, 13S) 9a-hydroxy-13-(3-(pentafluorosulfanyl) phenyl)-9a,10,12a,13-tetrahydrofuro[3,4-b]pyrido[3,2-f][1,10]phenanthrolin-12(9H)-one (8g)

Yield: 78% ^1^H NMR (500 MHz, DMSO-d_6_) *δ* 9.13 (dd, J = 1.5, 4.5 Hz, 1H, Ar*H*), 8.93 (dd, J = 1.5, 8.5 Hz, 1H, Ar*H*), 8.73 (dd, J = 1.5, 4.5 Hz, 1H, Ar*H*), 7.97 (dd, J = 1.5, 8.5 Hz, 1H, Ar*H*), 7.93 (t, *J* = 1.5 Hz, 1H, Ar*H*), 7.68 (dd, J = 4, 8.5 Hz, 1H, Ar*H*), 7.70 (ddd, J = 1, 2, 8.5 Hz, 1H, Ar*H*), 7.55 (s, 1H, N*H*-9), 7.54–7.47 (m, 2H, Ar*H*), 7.43 (t, J = 8 Hz, 1H, Ar*H*), 6.18 (s, 1H, O*H*-9a), 5.19 (s, 1H, C*H*-13), 4.59 (d, J = 8.5 Hz, 1H, C*H-*10), 4.24 (d, J = 8.5 Hz, 1H, C*H-*10), 3.66 (s, 1H, C*H-*12a); ^19^F NMR (DMSO-d_6_) *δ* 87.75 (qn, J = 148.7 Hz, 1F), 64.12 (d, J = 150.6 Hz, 4F); ^13^C NMR (125 MHz, DMSO-d_6_) *δ* 174.08 (*C*=O), 149.80 (Ar*CH*), 145.91 (Ar*C*), 145.37 (Ar*C*H), 144.07 (Ar*C*), 141.22 (Ar*C*), 135.73 (Ar*C*), 132.62 (Ar*C*H), 130.10 (Ar*C*H), 129.62 (Ar*C*H), 129.14 (Ar*C*H), 128.18 (Ar*C*), 126.93 (Ar*C*), 125.80 (m, Ar*C*H), 123.95 (m, Ar*C*H), 123.37 (Ar*C*H), 122.65 (Ar*C*H), 120.53 (Ar*C*), 103.34 (Ar*C*), 81.87 (*C*OH*-*9a), 74.53 (*C*H_2_*-*10), 49.77 (*C*H-12a), 36.16 (*C*H-13). MS (ESI): 510.1 [M+H]^+^. C_23_H_16_F_5_N_3_O_3_S calcd (C, 54.23; H, 3.17; N, 8.24); found (C, 54.39; H, 3.24; N, 8.47).

##### 4.1.1.8 (9aS, 12aR, 13R) and (9aR, 12aS, 13S) 9a-hydroxy-13-(3-methyl-5-trifluoromethoxyphenyl)-9a,10,12a,13-tetrahydrofuro[3,4-b]pyrido[3,2-f][1,10] phenanthrolin-12(9H)-one (8h)

Yield: 73% ^1^H NMR (500 MHz, DMSO-d_6_) *δ* 9.12 (dd, J = 1, 4 Hz, 1H, Ar*H*), 8.92 (dd, J = 1.5, 9 Hz, 1H, Ar*H*), 8.73 (ddd, J = 0.5, 1.5, 4.5 Hz, 1H, Ar*H*), 7.92 (dd, J = 1.5, 8.5 Hz, 1H, Ar*H*), 7.86 (dd, J = 4, 8.5 Hz, 1H, Ar*H*), 7.52–7.47 (m, 2H, Ar*H*), 7.25 (s, 1H, N*H*-9), 6.98 (s, 1H, Ar*H*), 7.95 (s, 1H, Ar*H*), 6.10 (s, 1H, O*H*-9a), 5.03 (s, 1H, C*H*-13), 4.58 (d, J = 8 Hz, 1H, C*H-*10), 4.23 (d, J = 8 Hz, 1H, C*H-*10), 3.60 (s, 1H, C*H-*12a), 2.27 (s, 3H, C*H*_*3*_); ^19^F NMR (DMSO-d_6_) *δ* −56.51; ^13^C NMR (125 MHz, DMSO-d_6_) *δ* 174.29 (*C*=O), 149.71 (Ar*C*H), 148.23 (q, ^3^*J*_*C-F*_ = 1.9 Hz, Ar*C*), 145.87 (Ar*C*), 145.30 (Ar*C*H), 144.98 (Ar*C*), 141.18 (Ar*C*), 139.99 (Ar*C*), 135.58 (Ar*C*), 130.09 (Ar*CH*), 129.71 (Ar*C*H), 128.35 (Ar*C*), 128.16 (Ar*C*H), 124.27 (q, ^1^*J*_*C-F*_ = 272.5 Hz, *CF*_*3*_), 123.29 (Ar*C*H), 122.60 (Ar*C*H), 120.56 (Ar*C*), 119.27 (Ar*C*H), 118.15 (Ar*C*H), 103.50 (Ar*C*), 81.83 (*C*OH*-*9a), 74.46 (*C*H_2_*-*10), 49.95 (*C*H-12a), 36.16 (*C*H-13), 20.82 (Ar*C*H_3_). MS (ESI): 482.1 [M+H]^+^, 504.1 [M+Na]^+^. C_25_H_18_F_3_N_3_O_4_ calcd (C, 62.37; H, 3.77; N, 8.72); found (C, 62.32; H, 3.75; N, 8.70).

##### 4.1.1.9 (9aS, 12aR, 13R) and (9aR, 12aS, 13S) 9a-hydroxy-13-(4-((trifluoromethyl)thio)phenyl)-9a,10,12a,13-tetrahydrofuro[3,4-b]pyrido [3,2-f][1,10]phenanthrolin-12(9H)-one (8i)

Yield: 79% ^1^H NMR (500 MHz, DMSO-d_6_) *δ* 9.12 (dd, J = 1.5, 4.5 Hz, 1H, Ar*H*), 8.92 (dd, J = 1.5, 8.5 Hz, 1H, Ar*H*), 8.72 (d, J = 1.5, 4.5 Hz, 1H, Ar*H*), 7.92 (dd, J = 1.5, 8.5 Hz, 1H, Ar*H*), 7.85 (dd, J = 4.5, 8.5 Hz, 1H, Ar*H*), 7.58 (d, J = 8.5 Hz, 2H, Ar*H*), 7.54 (s, 1H, N*H*-9), 7.47 (dd, J = 4.5, 8.5 Hz, 1H, Ar*H*), 7.45 (d, J = 8.5 Hz, 2H, Ar*H*), 6.11 (s, 1H, O*H*-9a), 5.11 (s, 1H, C*H*-13), 4.59 (d, J = 8.5 Hz, 1H, C*H-*10), 4.25 (d, J = 8.5 Hz, 1H, C*H-*10), 3.65 (s, 1H, C*H-*12a). ^19^F NMR (DMSO-d_6_) *δ* −42.03, ^13^C NMR (125 MHz, DMSO-d_6_) *δ* 174.28 (*C*=O), 149.72 (Ar*CH)*, 146.16 (Ar*C*), 145.88 (Ar*C*), 145.30 (Ar*CH)*, 141.23 (Ar*C*), 136.01 (Ar*C*H), 135.63 (Ar*C*), 130.20 (Ar*CH)*,130.05 (Ar*CH)*, 129.60 (q, ^*1*^*J*_*C-F*_ = 306.3 Hz, S*CF*_*3*_), 129.62 (Ar*C*H),128.30 (Ar*C*), 123.32 (Ar*C*H), 122.61 (Ar*CH*), 120.67 (q, ^*3*^*J*_*C-F*_ = 1.8 Hz, Ar*C*), 120.56 (Ar*C*), 103.46 (Ar*C*), 81.90 (*C*OH*-*9a), 74.54 (*C*H_2_*-*10), 49.81 (*C*H-12a), 36.11 (*C*H-13). MS (ESI): 484.1 [M+H]^+^, 506.1 [M+Na]^+^. C_24_H_16_F_3_N_3_O_3_S calcd (C, 59.62; H, 3.34; N, 8.69); found (C, 59.29; H, 3.35; N, 8.55).

##### 4.1.1.10 (9aS, 12aR, 13R) and (9aR, 12aS, 13S) 13-(3,5-bis(trifluoromethyl)phenyl)-9a-hydroxy-9a,10,12a,13-tetrahydrofuro[3,4-b]pyrido[3,2-f][1,10] phenanthrolin-12(9H)-one (8j)

Yield: 74% ^1^H NMR (500 MHz, DMSO-d_6_) *δ* 9.14 (dd, J = 1, 4 Hz, 1H, Ar*H*), 8.95 (dd, J = 1.5, 9 Hz, 1H, Ar*H*), 8.74 (dd, J = 1.5, 4 Hz, 1H, Ar*H*), 8.00 (dd, J = 1.5, 8.5 Hz, 1H, Ar*H*), 7.98 (s, 2H, Ar*H*), 7.94 (s, 1H, Ar*H*), 7.88 (dd, J = 4, 8.5 Hz, 1H, Ar*H*), 7.63 (s, 1H, N*H*-9), 7.50 (dd, J = 4, 8.5 Hz, 1H, Ar*H*), 6.31 (s, 1H, O*H*-9a), 5.33 (s, 1H, C*H*-13), 4.60 (d, J = 8.5 Hz, 1H, C*H-*10), 4.23 (d, J = 8.5 Hz, 1H, C*H-*10), 3.75 (s, 1H, C*H-*12a); ^19^F NMR (DMSO-d_6_) *δ* −61.15; ^13^C NMR (125 MHz, DMSO-d_6_) *δ* 173.85 (*C*=O), 149.98 (Ar*CH*), 145.93 (Ar*C*), 145.51 (Ar*C*H), 141.16 (Ar*C*), 135.95 (Ar*C*), 130.19 (Ar*C*H), 130.02 (Ar*C*), 129.76 (Ar*C*), 129.65 (Ar*C*H), 129.50 (m, Ar*C*H), 128.06 (Ar*C*), 123.51 (Ar*C*H), 122.74 (Ar*C*H), 123.77 (q, ^*1*^*J*_*C-F*_ = 271.4 Hz, *C*F_3_), 120.64 (m, Ar*C*H), 120.49 (Ar*C*), 102.61 (Ar*C*), 81.81 (*C*OH*-*9a), 74.45 (*C*H_2_*-*10), 49.52 (*C*H-12a), 35.66 (*C*H-13). MS (ESI): 520.1 [M+H]^+^, 542.1 [M+Na]^+^. C_25_H_15_F_6_N_3_O_3_ calcd (C, 57.81; H, 2.91; N, 8.09); found (C, 57.60; H, 2.96; N, 8.21).

##### 4.1.1.11 (9aS, 12aR, 13R) and (9aR, 12aS, 13S) 13-(4-ethoxy-3-(trifluoromethyl) phenyl)-9a-hydroxy-9a,10,12a,13-tetrahydrofuro[3,4-b]pyrido[3,2-f][1,10] phenanthrolin-12(9H)-one (8k)

Yield: 73% ^1^H NMR (500 MHz, DMSO-d_6_) *δ* 9.12 (dd, J = 1.5, 4 Hz, 1H, Ar*H*), 8.92 (dd, J = 1.5, 8.5 Hz, 1H, Ar*H*), 8.73 (dd, J = 1.5, 4 Hz, 1H, Ar*H*), 7.96 (dd, J = 1.5, 8.5 Hz, 1H, Ar*H*), 7.85 (dd, J = 4.5, 8.5 Hz, 1H, Ar*H*), 7.60 (d, J = 2.5 Hz, 1H, Ar*H*), 7.54–7.46 (m, 2H, N*H*-9, Ar*H*), 7.39 (dd, J = 2, 9 Hz, 1H, Ar*H*), 7.05 (d, J = 8.5, 1H, Ar*H*), 6.10 (s, 1H, O*H*-9a), 5.06 (s, 1H, C*H*-13), 4.58 (d, J = 8 Hz, 1H, C*H-*10), 4.22 (d, J = 8 Hz, 1H, C*H-*10), 4.11–4.04 (m, 2H, CH_3_C*H*_*2*_), 3.60 (s, 1H, C*H-*12a), 1.29 (t, J = 7 Hz, 3H, C*H*_3_CH_2_); ^19^F NMR (DMSO-d_6_) *δ* −60.64; ^13^C NMR (125 MHz, DMSO-d_6_) *δ* 174.32 (*C*=O), 154.90 (Ar*C*), 149.67 (Ar*CH*), 145.85 (Ar*C*), 145.29 (Ar*C*H), 141.20 (Ar*C*), 135.48 (Ar*C*), 133.98 (Ar*C*H), 133.96 (Ar*C*), 130.04 (Ar*C*H), 129.80 (Ar*C*H), 128.33 (Ar*C*), 126.70 (q, ^*3*^*J*_*C-F*_ = 5.1 Hz, Ar*C*H), 123.83 (q, ^*1*^*J*_*C-F*_ = 270.6 Hz, *CF*_*3*_), 123.33 (Ar*C*H), 122.59 (Ar*C*H), 120.55 (Ar*C*), 116.77 (q, ^*2*^*J*_*C-F*_ = 29 Hz, Ar*C*), 113.31 (Ar*C*H), 103.87 (Ar*C*), 81.91 (*C*OH*-*9a), 74.55 (*C*H_2_*-*10), 64.39 (*C*H_2_CH_3_), 50.00 (*C*H-12a), 35.32 (*C*H-13), 14.39 (CH_2_*C*H_3_). MS (ESI): 496.1 [M+H]^+^, 518.1 [M+Na]^+^. C_26_H_20_F_3_N_3_O_4_ calcd (C, 63.03; H, 4.07; N, 8.48); found (C, 62.68; H, 3.98; N, 8.55).

##### 4.1.1.12 (9aS, 12aR, 13R) and (9aR, 12aS, 13S) 13-(4-(difluoromethoxy)phenyl)-9a-hydroxy-9a,10,12a,13-tetrahydrofuro[3,4-b]pyrido[3,2-f][1,10]phenanthrolin-12(9H)-one (8l)

Yield: 65% ^1^H NMR (500 MHz, DMSO-d_6_) *δ* 9.11 (dd, J = 1.5, 4.5 Hz, 1H, Ar*H*), 8.91 (dd, J = 1.5, 9 Hz, 1H, Ar*H*), 8.72 (dd, J = 1.5, 4.5 Hz, 1H, Ar*H*), 7.93 (dd, J = 1.5, 9 Hz, 1H, Ar*H*), 7.85 (dd, J = 4.5, 8.5 Hz, 1H, Ar*H*), 7.50 (s, 1H, N*H*-9), 7.48 (dd, J = 4.5, 8.5 Hz, 1H, Ar*H*), 7.34 (d, J = 8.5, 2H, Ar*H*), 7.16 (t, ^*2*^*J*_*H-F*_ = 74.5, 1H, ArCF_2_*H*), 7.03 (d, J = 9, 2H, Ar*H*), 6.06 (s, 1H, O*H*-9a), 5.04 (s, 1H, C*H*-13), 4.58 (d, J = 8.5 Hz, 1H, C*H-*10), 4.23 (d, J = 8.5 Hz, 1H, C*H-*10), 3.57 (s, 1H, C*H-*12a); ^19^F NMR (DMSO-d_6_) *δ* −81.85; ^13^C NMR (125 MHz, DMSO-d_6_) *δ* 174.47 (*C*=O), 149.62 (Ar*CH*), 149.35 (Ar*C*), 145.84 (Ar*C*), 145.23 (Ar*CH*), 141.24 (Ar*C*), 139.26 (Ar*C*), 135.42 (Ar*C*), 130.05 (Ar*CH*), 130.01 (Ar*CH*), 129.74 (Ar*CH*), 128.37 (Ar*C*), 123.26 (Ar*C*H), 122.57 (Ar*C*H), 120.58 (Ar*C*), 118.45 (Ar*C*H), 116.26 (t, ^*1*^*J*_*C-F*_ = 255.9 Hz, *C*HF_2_), 104.08 (Ar*C*), 81.93 (*C*OH*-*9a), 74.55 (*C*H_2_*-*10), 50.06 (*C*H-12a), 35.77 (*C*H-13). HRMS calcd for C_24_H_17_F_2_N_3_O_4_ (M + H)+, 450.1260; found, 452.1260.

##### 4.1.1.13 (9aS, 12aR, 13R) and (9aR, 12aS, 13S) 9a-hydroxy-13-(2-methyl-5-(trifluoromethoxy)phenyl)-9a,10,12a,13-tetrahydrofuro[3,4-b]pyrido[3,2-f][1,10]phenanthrolin-12(9H)-one (8m)

Yield: 61% ^1^H NMR (500 MHz, DMSO-d_6_) *δ* 9.14 (dd, J = 1, 4 Hz, 1H, Ar*H*), 8.94 (dd, J = 1.5, 8.5 Hz, 1H, Ar*H*), 8.72 (dd, J = 1.5, 4 Hz, 1H, Ar*H*), 7.88 (dd, J = 4.5, 8.5 Hz, 1H, Ar*H*), 7.56–7.52 (m, 2H, N*H*-9, Ar*H*), 7.48–7.42 (m, 2H, Ar*H*), 7.08 (d, J = 8.5 Hz, 1H, Ar*H*), 6.66 (d, J = 2 Hz, 1H, Ar*H*), 6.14 (s, 1H, O*H*-9a), 5.03 (s, 1H, C*H*-13), 4.59 (d, J = 8 Hz, 1H, C*H-*10), 4.22 (d, J = 8.5 Hz, 1H, C*H-*10), 3.51 (s, 1H, C*H-*12a), 2.67 (s, 3H, C*H*_*3*_); ^19^F NMR (DMSO-d_6_) *δ* −56.96; ^13^C NMR (125 MHz, DMSO-d_6_) *δ* 174.45 (*C*=O), 149.78 (Ar*C*H), 147.26 (Ar*C*), 145.83 (Ar*C*), 145.31 (Ar*C*H), 142.93 (Ar*C*), 141.30 (Ar*C*), 136.32 (Ar*C*), 134.44 (Ar*C*), 132.08 (Ar*CH*), 130.13 (Ar*C*H), 128.06 (*C*F_*3*_), 129.54 (Ar*C*H), 123.37 (Ar*C*H), 122.68 (Ar*C*H), 120.61 (Ar*C*), 120.58 (Ar*C*), 121.30 (Ar*C*H), 118.75 (Ar*C*H), 103.65 (Ar*C*), 81.61 (*C*OH*-*9a), 74.29 (*C*H_2_*-*10), 47.75 (*C*H-12a), 33.23 (*C*H-13), 18.30 (Ar*C*H_3_). MS (ESI): 482.1 [M+H]^+^. C_26_H_20_F_3_N_3_O_4_ calcd (C, 62.37; H, 3.77; N, 8.72); found (C, 61.98; H, 3.56; N, 8.91).

##### 4.1.1.14 (9aS, 12aR, 13R) and (9aR, 12aS, 13S) 9a-hydroxy-13-(4-(pentafluoro sulfanyl)phenyl)-9a,10,12a,13-tetrahydrofuro[3,4-b]pyrido[3,2-f][1,10] phenanthrolin-12(9H)-one (8n)

Yield: 76% 1H NMR (500 MHz, DMSO-d_6_) *δ* 9.13 (dd, J = 1.5, 4.5 Hz, 1H, Ar*H*), 8.93 (dd, J = 1.5, 8.5 Hz, 1H, Ar*H*), 8.73 (dd, J = 1.5, 4.5 Hz, 1H, Ar*H*), 7.92 (dd, J = 1.5, 8.5 Hz, 1H, Ar*H*), 7.87 (dd, J = 4, 8 Hz, 1H, Ar*H*), 7.77 (d, J = 9, 2H, Ar*H*), 7.60 (s, 1H, N*H*-9), 7.53–7.47 (m, 3H, Ar*H*), 6.16 (s, 1H, O*H*-9a), 5.16 (s, 1H, C*H*-13), 4.59 (d, J = 8.5 Hz, 1H, C*H-*10), 4.22 (d, J = 8.5 Hz, 1H, C*H-*10), 3.66 (s, 1H, C*H-*12a); ^19^F NMR (DMSO-d_6_) *δ* 87.97 (qn, J = 150.9 Hz, 1F), 64.35 (d, J = 150.4 Hz, 4F); ^13^C NMR (125 MHz, DMSO-d_6_) *δ* 174.14 (*C*=O), 149.79 (Ar*C*H), 147.02 (Ar*C*), 145.84 (Ar*C*), 145.32 (Ar*C*H), 141.15 (Ar*C*), 135.74 (Ar*C*), 134.94 (m, ArC), 130.09 (Ar*CH*), 129.67 (Ar*C*H), 129.49 (Ar*C*H), 128.26 (Ar*C*), 125.68 (Ar*C*H), 123.46 (Ar*C*H), 122.67 (Ar*C*H), 120.56 (Ar*C*), 103.11 (Ar*C*), 81.85 (*C*OH*-*9a), 74.55 (*C*H_2_*-*10), 49.79 (*C*H-12a), 35.84 (*C*H-13). MS (ESI): 510.1 [M+H]^+^. C_23_H_16_F_5_N_3_O_3_S calcd (C, 54.23; H, 3.17; N, 8.24); found (C, 53.96; H, 2.98; N, 8.42).

##### 4.1.1.15 (9aS, 12aS, 13S) and (9aR, 12aR, 13R) 9a-hydroxy-13-(2-methoxy-4-(trifluoromethoxy)phenyl)-9a,10,12a,13-tetrahydrofuro[3,4-b]pyrido[3,2-f][1,10]phenanthrolin-12(9H)-one (8°)

Yield: 71% ^1^HNMR (500 MHz, DMSO-d_6_) *δ* 9.17 (dd, J = 1.5, 4 Hz, 1H, Ar*H*), 8.96 (dd, J = 1.5, 8.5 Hz, 1H, Ar*H*), 8.77 (dd, J = 1.5, 4 Hz, 1H, Ar*H*), 7.90 (dd, J = 4, 8.5 Hz, 1H, Ar*H*), 7.68 (dd, J = 1.5, 8.5 Hz, 1H, Ar*H*), 7.56–7.52 (m, 2H, Ar*H,* N*H*-9), 7.16 (d, J = 2 Hz, 1H, Ar*H*), 6.81 (d, J = 8.5 Hz, 1H, Ar*H*), 6.67 (d, J = 8.5 Hz, 1H, Ar*H*), 6.05 (s, 1H, O*H*-9a), 5.31 (s, 1H, C*H*-13), 4.62 (d, J = 8 Hz, 1H, C*H-*10), 4.27 (d, J = 8.5 Hz, 1H, C*H-*10), 4.10 (s, 3H, OC*H*_*3*_), 3.51 (s, 1H, C*H-*12a); ^19^F NMR (DMSO-d_6_) *δ* 56.58; ^13^C NMR (125 MHz, DMSO-d_6_) *δ* 174.39 (*C*=O), 157.07 (Ar*C*), 149.64 (Ar*C*H), 149.12 (q, ^*2*^*J*_*C-F*_ = 30.5 Hz, Ar*C*), 145.85 (Ar*C*), 145.27 (Ar*C*H), 141.27 (Ar*C*), 136.02 (Ar*C*), 130.55 (Ar*CH*), 129.94 (Ar*C*H), 129.09 (Ar*C*H), 128.37 (*CF*_*3*_), 128.20 (Ar*C*), 123.47 (Ar*C*H), 122.59 (Ar*C*H), 120.51 (Ar*C*), 111.69 (Ar*C*H), 104.45 (Ar*C*H), 103.37 (Ar*C*), 81.88 (*C*OH*-*9a), 74.53 (*C*H_2_*-*10), 56.41 (O*C*H_3_), 47.59 (*C*H-12a), 30.64 (*C*H-13). HRMS calcd for: C_25_H_18_F_3_N_3_O_5_ (M + H)+, 498.1257; found, 498.1271.

##### 4.1.1.16 (9aS, 12aR, 13R) and (9aR, 12aS, 13S) 9a-hydroxy-13-((5-methoxy-3,4-methylenedioxy)phenyl)-9a,10,12a,13-tetrahydrofuro[3,4-b]pyrido[3,2-f][1,10]phenanthrolin-12(9H)-one (8p)

Yield: 74% ^1^H NMR (500 MHz, DMSO-d_6_) *δ* 9.11 (dd, J = 1.5, 4 Hz, 1H, Ar*H*), 8.92 (dd, J = 1.5, 8.5 Hz, 1H, Ar*H*), 8.74 (dd, J = 1.5, 4 Hz, 1H, Ar*H*), 8.02 (dd, J = 1, 8.5 Hz, 1H, Ar*H*), 7.85 (dd, J = 4, 8.5 Hz, 1H, Ar*H*), 7.53 (dd, J = 4, 8.5 Hz, 1H, Ar*H*), 7.48 (s, 1H, Ar*H,* N*H*-9), 6.74 (d, J = 1.5 Hz, 1H, Ar*H*), 6.35 (d, J = 1.5 Hz, 1H, Ar*H*), 6.06 (s, 1H, O*H*-9a), 5.90 (s, 1H, OC*H*_*2*_O), 5.86 (s, 1H, OC*H*_*2*_O), 4.94 (s, 1H, C*H*-13), 4.57 (d, J = 8.5 Hz, 1H, C*H-*10), 4.22 (d, J = 8.5 Hz, 1H, C*H-*10), 3.78 (s, 3H, OC*H*_*3*_), 3.56 (s, 1H, C*H-*12a); ^13^C NMR (125 MHz, DMSO-d_6_) *δ* 174.52 (*C*=O), 149.57 (Ar*C*H), 148.06 (Ar*C*), 145.54 (Ar*C*), 145.03 (Ar*C*H), 142.98 (Ar*C*), 137.19 (Ar*C*), 135.39 (Ar*C*), 135.32 (Ar*C*), 133.24 (Ar*C*), 130.22 (Ar*C*H), 130.13 (Ar*C*H), 128.63 (Ar*C*), 123.34 (Ar*C*H), 122.64 (Ar*C*H), 120.61 (Ar*C*), 120.57 (Ar*C*), 108.27 (Ar*C*H), 104.34 (O*C*H_2_O), 102.48 (Ar*C*H), 81.77 (*C*OH*-*9a), 74.45 (*C*H_2_*-*10), 56.31 (O*C*H_3_), 50.28 (*C*H-12a), 36.49 (*C*H-13). HRMS calcd for: C_25_H_19_N_3_O_6_ (M + H)+, 458.1347; found, 458.1338.

##### 4.1.1.17 (9aS, 12aR, 13R) and (9aR, 12aS, 13S) 13-((3,4-difluoromethylenedioxy)phenyl)-9a-hydroxy-9a,10,12a,13-tetrahydrofuro[3,4-b]pyrido[3,2-f][1,10]phenanthrolin-12(9H)-one (8q)

Yield: 77% ^1^H NMR (500 MHz, DMSO-d_6_) *δ* 9.12 (dd, J = 1.5, 4.5 Hz, 1H, Ar*H*), 8.92 (dd, J = 1.5, 8.5 Hz, 1H, Ar*H*), 8.73 (dd, J = 1.5, 4 Hz, 1H, Ar*H*), 7.97 (dd, J = 1.5, 8.5 Hz, 1H, Ar*H*), 7.85 (dd, J = 4, 8 Hz, 1H, Ar*H*), 7.53 (s, 1H, N*H*-9), 7.50 (dd, J = 4.5, 8.5 Hz, 1H, Ar*H*), 7.32 (d, J = 1.5 Hz, 1H, Ar*H*), 7.24 (d, J = 8.5 Hz, 1H, Ar*H*), 7.10 (dd, J = 2, 8.5 Hz, 1H, Ar*H*), 6.07 (s, 1H, O*H*-9a), 5.09 (s, 1H, C*H*-13), 4.58 (d, J = 8.5 Hz, 1H, C*H-*10), 4.22 (d, J = 8 Hz, 1H, C*H-*10), 3.61 (s, 1H, C*H-*12a); ^19^F NMR (DMSO-d_6_) *δ* 48.89.^13^C NMR (125 MHz, DMSO-d_6_) *δ* 174.24 (*C*=O), 149.72 (Ar*C*H), 145.90 (Ar*C*), 145.33 (Ar*C*H), 142.56 (Ar*C*), 141.24 (Ar*C*), 141.19 (Ar*C*), 139.27 (Ar*C*), 135.56 (Ar*C*), 131.11 (t, ^*1*^*J*_*C-F*_ = 254.3 Hz, *C*F_2_), 130.05 (Ar*C*H), 129.72 (Ar*C*H), 128.30 (Ar*C*), 124.37 (Ar*C*H), 123.39 (Ar*C*H), 122.60 (Ar*C*H), 120.56 (Ar*C*), 110.32 (Ar*C*H), 109.58 (Ar*C*H), 103.67 (Ar*C*), 81.92 (*C*OH*-*9a), 74.56 (*C*H_2_*-*10), 50.08 (*C*H-12a), 36.09 (*C*H-13); HRMS calcd for: C_24_H_15_F_2_N_3_O_5_ (M + H)+, 464.1042; found, 464.1053.

### 4.2 X-ray crystal structure determination of compound 8c

Single-crystal XRD data were collected on an Agilent SupaNova Dual Atlas diffractometer with a mirror monochromator using either Cu (λ = 1.5418 Å) radiation and equipped with an Oxford Cryosystems cooling apparatus. Crystal structures were solved and refined using SHELX. (Sheldrick, G. M. Acta Crystallogr., Sect. A 2008, 64, 112.) Non-hydrogen atoms were refined with anisotropic displacement parameters. Hydrogen atoms were inserted in idealized positions, and a riding model was used with Uiso set at 1.2 or 1.5 times the value of Ueq for the atom to which they are bonded. C_24_H_16_F_3_N_3_O_4_, FW = 467.40, T = 150(2) K, λ = 1.54184 Å, Monoclinic, P2_1_/c, a = 20.633(2) Å, b = 6.0168(6) Å, c = 18.8375(18) Å, β = 115.552(12)°, V = 2109.9(4) Å3, Z = 4, ρ(calculated) = 1.471 Mg/m3, μ = 1.023 mm-1, Crystal size = 0.238 x 0.031 × 0.020 mm^3^, Reflections collected = 8334, Independent reflections = 4181, R(int) = 0.0723, Parameters = 308, G-o-f = 1.015, Final R1 = 0.0588, wR2 = 0.1111 on (I > 2σ(I)), R1 = 0.1240, wR2 = 0.1404 on all data. In the crystal structure, the molecules are linked by two types of hydrogen bonds [N1–H1 … O2 with geometry N … O = 2.888(4) Å, N–H … O angle = 154.8° and O3–H3 … N3 with O … N = 2.804(3) Å, O–H … N = 158.6°)] to form layers parallel to the *bc* plane. CCDC 1426447 contains the supplementary crystallographic data for this paper. The data can be obtained free of charge from The Cambridge Crystallographic Data Centre via www.ccdc.cam.ac.uk/getstructures.

### 4.3 Docking study

We used the X-ray co-crystal structure of tubulin with podophyllotoxin in the Protein Data Bank PDB ID: 1SA1 [23]. The protein structure for the docking study was prepared with Protein Preparation Workflow in Schrödinger software. Constrained energy minimization was performed with the OPLS_2005 force field. Compounds (**8a-q**) were sketched in MOE (version 2014.09, Chemical Computing Group Inc, Montreal, Quebec, www.chemcomp.com. Canada) [24]. Molecular docking was performed with Glide SP (version 9.5) in Maestro (Glide, version 9.5, Schrödinger, LLC, New York, NY. http://www. schrodinger.com) [25]. The docking box was centred on the ligand of the crystal structure. The molecule database used for docking was processed with the LigPreP tool in Maestro. The best scoring poses of GlideScore were evaluated.

### 4.4 Cell proliferation assay

A modified propidium iodide (PI) based monolayer assay [22] was used to assess the anti-cancer activity of the compounds. Briefly, cells were harvested from exponential phase cultures, counted and plated in 96-well flat-bottom microtiter plates at a cell density of 8.000–12.000 cells/well. After a 24 h recovery period to allow the cells to resume exponential growth, 10 μl of culture medium (six control wells/plate) or culture medium with test compound were added. The compounds were applied in half-log increments at 10 concentrations in triplicate. After a total treatment period of 96 h, cells were washed with 200 μl PBS to remove dead cells and debris. Then, 200 μl of a solution containing 7 μg/mL propidium iodide (PI) was added. After an incubation period of 1–2 h at room temperature, fluorescence (FU) was measured using the EnSpire Multimode Plate Reader (excitation λ = 530 nm, emission λ = 620 nm) to quantify the amount of attached viable cells. IC_50_ values were calculated by 4-parameter non-linear curve fit using Oncotest Warehouse Software. For calculation of mean IC_50_ values the geometric mean was used.

### 4.5 Subcellular analysis of microtubule organisation

#### 4.5.1 Chemicals and reagents

Podophyllotoxin and **8e** and **8b** were dissolved in DMSO to produce a stock concentration of 2.5 mM and stored at 4 °C. DMSO and the anti-α-tubulin antibody (#T9026) were from Sigma–Aldrich (Dorset, UK). RPMI 1640, FBS, the nuclear probe Hoechst 33342 and Alexa-488 conjugated anti-mouse antibody (#A-21200) were from Life Technologies (Paisley, UK).

#### 4.5.2 Cell culture

MCF-7 cells were maintained in RPMI 1640 supplemented with 10% FBS (complete media). The cells were obtained from ATCC and routinely tested for mycoplasma infection. For immunofluorescence experiments: 180,000 cells were seeded onto glass coverslips in 12-well plates and cultured to 70–80% confluency at 37 °C/5% CO_2_ in complete media.

#### 4.5.3 Microtubule immunofluorescence assay: cell culture and treatments

Following cell seeding (42 h) the media was replaced with fresh complete media containing 250 nM of podophyllotoxin, compound **8e** and **8b** or 0.01% DMSO. The cells were treated for 6 h with drug or diluent control then subjected to downstream immunofluorescence analysis (see tubulin immunofluorescence protocol).

#### 4.5.4 Microtubule repolymerisation assay: cell culture and treatments

##### 4.5.4.1 First variation, pre-treatment

Following cell seeding (42 h) the media was replaced with drug (or diluent control) containing complete media and cells were treated for 6 h with 250 nM of podophyllotoxin, compound **8e**, **8b** or 0.01% DMSO. The plates were then placed on ice and cells were incubated at 4 °C for 30 min to depolymerise the tubulin cytoskeleton [27], [28], [30]. One set of cover slips for each treatment condition was prepared for tubulin immunofluorescence (see protocol below) and the remaining sets of cover slips were returned to the incubator (37 °C, 5% CO_2_) for 30 min to allow re-polymerisation of microtubules. Tubulin immunofluorescence was performed after the cells had been rewarmed for 30 min (see below).

##### 4.5.4.2 Second variation, no pre-treatment

Following cell seeding (42 h), the plates were placed on ice for 30 min to cold-depolymerise the tubulin cytoskeleton. Control cold-depolymerised coverslips were then immediately prepared for tubulin immunofluorescence and the remaining coverslips were rewarmed and treated with 250 nM of podophyllotoxin, compound **8e**, **8b** or 0.01% DMSO for 30 min (37 °C, 5% CO_2_). Together with the controls these were then subjected to tubulin immunofluorescence.

#### 4.5.5 Tubulin immunofluorescence

The media was aspirated and the cells were washed once in ice-cold PBS before being fixed in 100% methanol at −20 °C for 10 min. The cells were then washed thrice in PBS and stored overnight at 4 °C. The following day cells were incubated in 150 μL of blocking solution (2% FBS [v/v], 2% BSA [w/v] in PBS pH 7.4) for 30 min. Blocking was followed by 1 h incubation with 100 μl of mouse anti-α tubulin antibody diluted 1:2000 in blocking solution. Cells were then washed 3 × 5min in PBS before being incubated with Alexa488 labelled, anti-mouse secondary antibody (1:400) and Hoechst 33342 (1 μg/ml) for 30 min. The cells were washed a final 3 × 5 min in PBS before coverslips were dipped once into PBS, once into dH_2_O and mounted onto glass slides with 12 μL of mounting medium (Dako oil). Three independent experiments were carried out in duplicate.

#### 4.5.6 Confocal microscopy

Confocal fluorescence microscopy analysis was conducted on a Leica SP5 inverted confocal laser scanning microscope. The microscope was equipped with a 63x oil-immersion objective and the 405 and 488 nm lasers were used. Gain and offset settings were optimised for each fluorescent channel within an experiment. Images were recorded using the sequential scanning mode to prevent fluorescence channel crosstalk/bleed-through. Images were scanned at 100 Hz with a line average of three to reduce noise.

## Appendix A Supplementary data

Spectroscopic and analytical data (^1^H, ^13^C, ^19^F NMR; mass spectrometry and microanalysis) for all final compounds are available. CCDC: 1426447; contains the supplementary crystallographic data for this work. The data can be obtained free of charge from The Cambridge Crystallographic Data Centre via www.ccdc.cam.ac.uk/getstructures.

### Mol Files

The following ZIP file contains the MOL files of the most important compounds referred to in this article.

MOL file ZIP file containing the MOL files of the most important compounds in this article.
