# Supplementary material for: Novel cis-selective and non-epimerisable C3 hydroxy azapodophyllotoxins targeting microtubules in cancer cells
Source: Eur J Med Chem. 2016 Mar 3;110:311–25. doi: 10.1016/j.ejmech.2015.12.037 (PMC4762250; doi:10.1016/j.ejmech.2015.12.037)

Supplementary data

Novel *cis*-selective and non-epimerisable C3 hydroxy azapodophyllotoxins as potent tubulin polymerisation inhibitors

Sahar Kandil[1], Jennifer M. Wymant[1], Benson M. Kariuki[2], Arwyn T. Jones[1], Christopher McGuigan[1] and Andrew D. Westwell[*1]

[1]Cardiff School of Pharmacy & Pharmaceutical Sciences, Cardiff University, King Edward VII Avenue, Cardiff, CF10 3NB, Wales, United Kingdom, [2]School of Chemistry, Cardiff University, Park Place, Cardiff, CF10 3AT, Wales, United Kingdom.

Contents;

Spectroscopic and analytical data (^{1}H, ^{13}C, ^{19}F NMR; mass spectrometry and microanalysis) for all final compounds.

[*] Corresponding author. E-mail: WestwellA@cf.ac.uk

CARDIFF UNIVERSITY
PRIFYSGOL CAERDYDD

8a

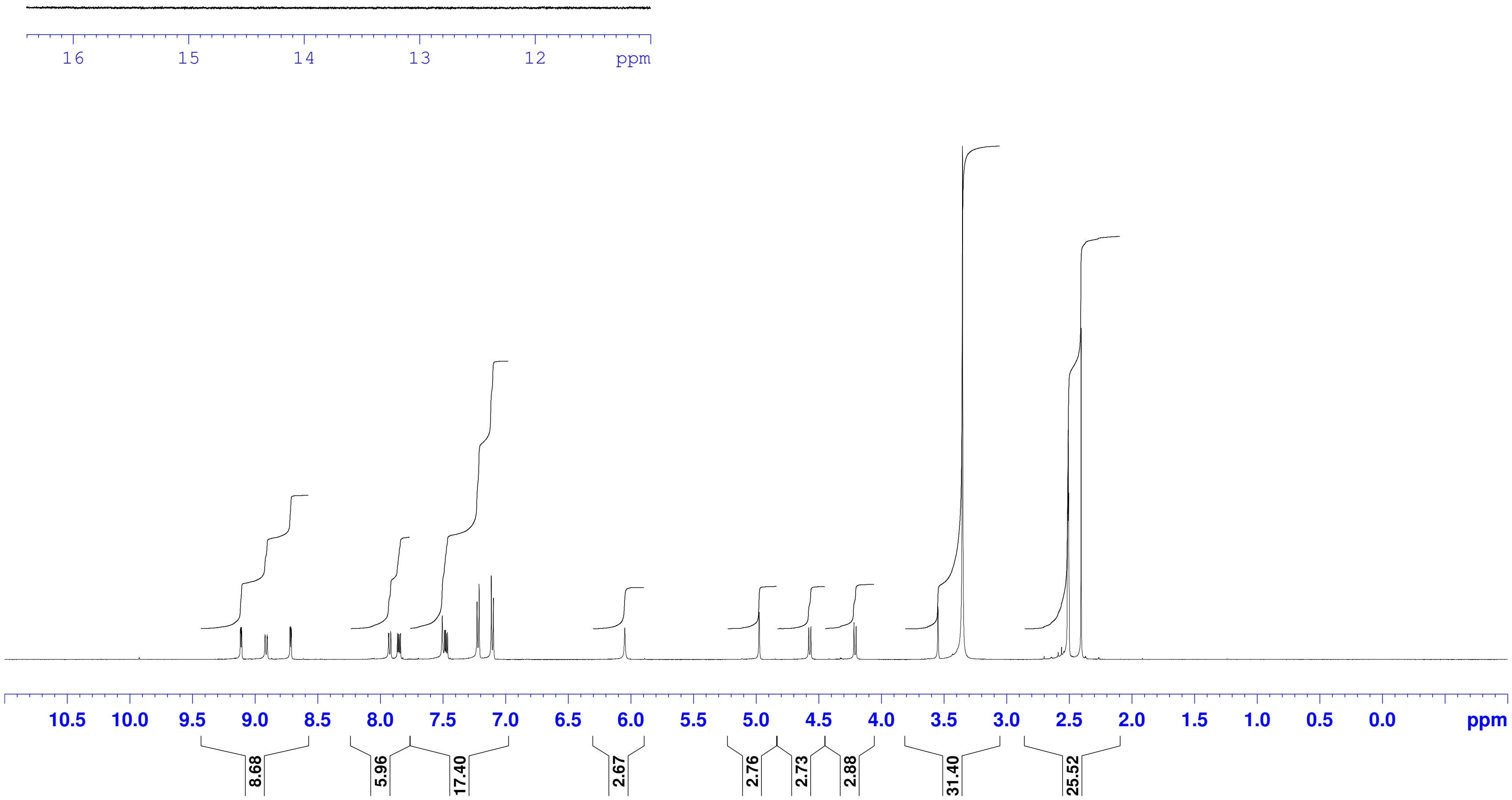

NAME	CM-SK81P
EXPNO	7
PROCNO	1
Date_	20141001
Time	0.35
INSTRUM	Avance500
PROBHD	5 mm QNP 1H/13
PULPROG	zg30
TD	65536
SOLVENT	DMSO
NS	64
DS	2
SWH	10330.578 Hz
FIDRES	0.157632 Hz
AQ	3.1719923 sec
RG	645
DW	48.400 use
DE	6.50 use
TE	290.0 K
D1	1.00000000 sec
TD0	1

======== CHANNEL f1 =======

NUC1	1H
P1	11.50 use
PL1	-1.00 dB
PL1W	11.38419914 W
SFO1	500.1330885 MHz
SI	32768
SF	500.1300000 MHz
WDW	EM
SSB	0
LB	0.30 Hz
GB	0
PC	1.00

8a

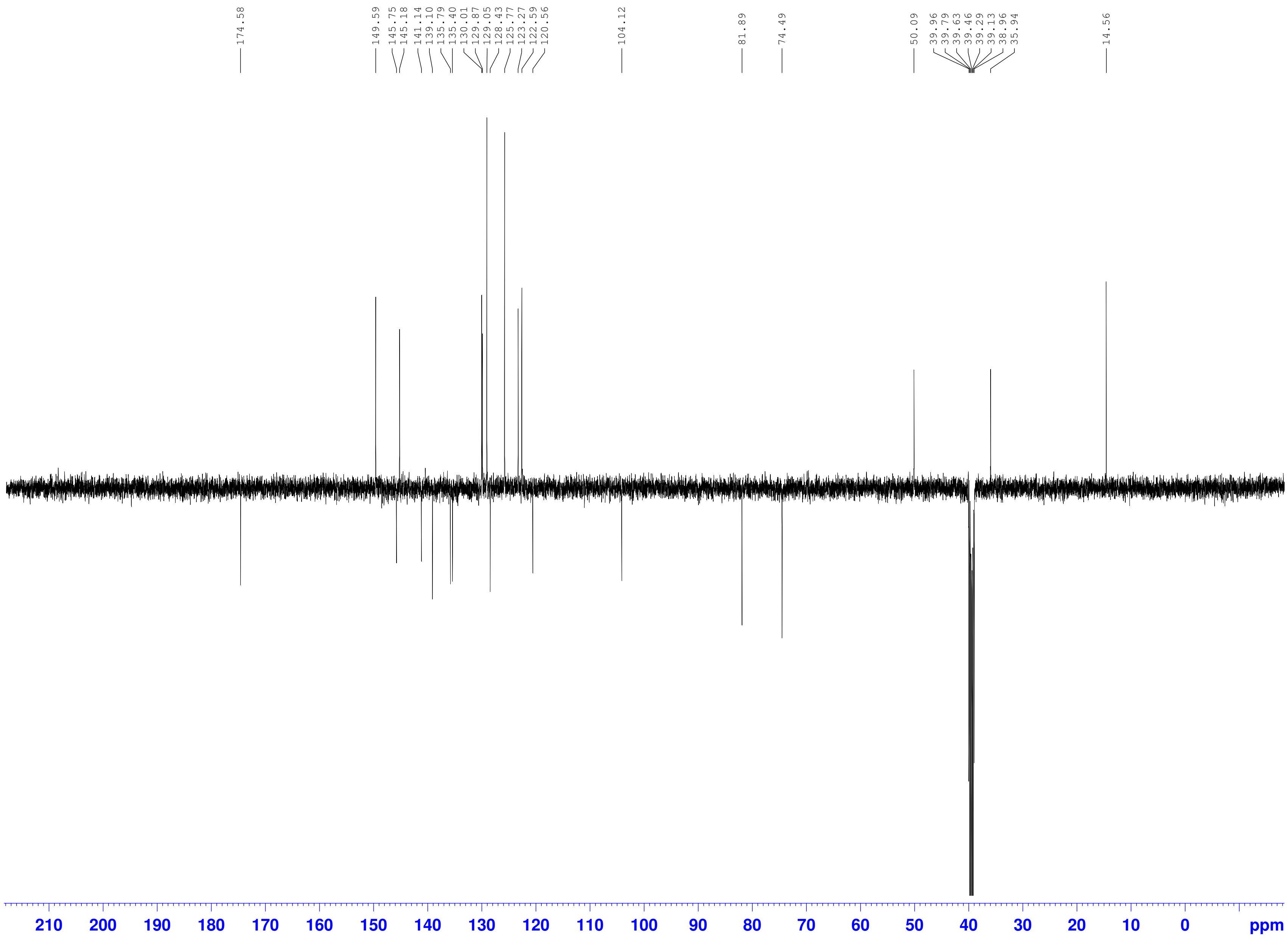

NAME	CM-SK81P	
EXPNO	6	
PROCNO	1	
Date_	20140930	
Time	22.23	
INSTRUM	Avance500	
PROBHD	5 mm QNP 1H/13	
PULPROG	pendant	
TD	65536	
SOLVENT	DMSO	
NS	2560	
DS	4	
SWH	29761.904	Hz
FIDRES	0.454131	Hz
AQ	1.1010548	sec
RG	3250	
DW	16.800	usec
DE	12.00	usec
TE	290.4	K
CNST2	145.0000000	
D1	2.00000000	sec
D4	0.00172414	sec
D12	0.00002000	sec
D15	0.00431034	sec
D20	0.00345000	sec
TD0	20	
======== CHANNEL f1 ========		
NUC1	13C	
P1	7.20	usec
P2	14.40	usec
PL1	-2.00	dB
PL1W	101.27846527	W
SFO1	125.7703643	MHz
======== CHANNEL f2 ========		
CPDPRG2	waltz16	
NUC2	1H	
P3	11.50	usec
P4	23.00	usec
PCPD2	80.00	usec
PL2	-2.00	dB
PL12	14.85	dB
PL2W	14.33185768	W
PL12W	0.29600734	W
SFO2	500.1320005	MHz
SI	32768	
SF	125.7578519	MHz
WDW	EM	
SSB	0	
LB	1.00	Hz
GB	0	
PC	1.40	

SK81P MW=429?
(MeOH)/MeOH + NH4OAc
C24H19N3O3S

EPSRC National Facility Swansea
LTQ Orbitrap XL

Dr AD Westwell
20/07/2015 09:29:00

8a

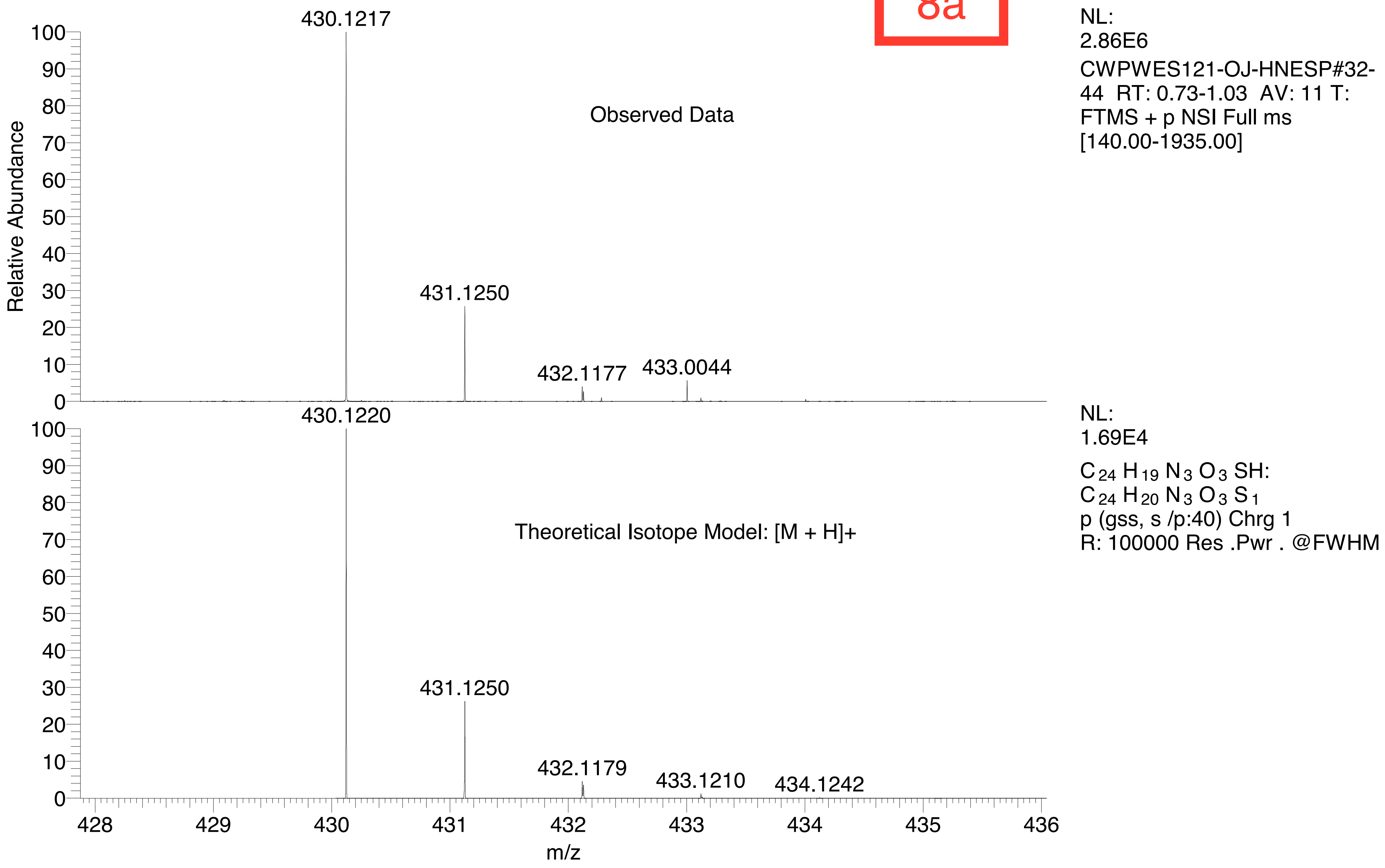

8b

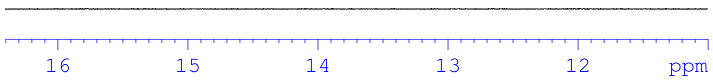

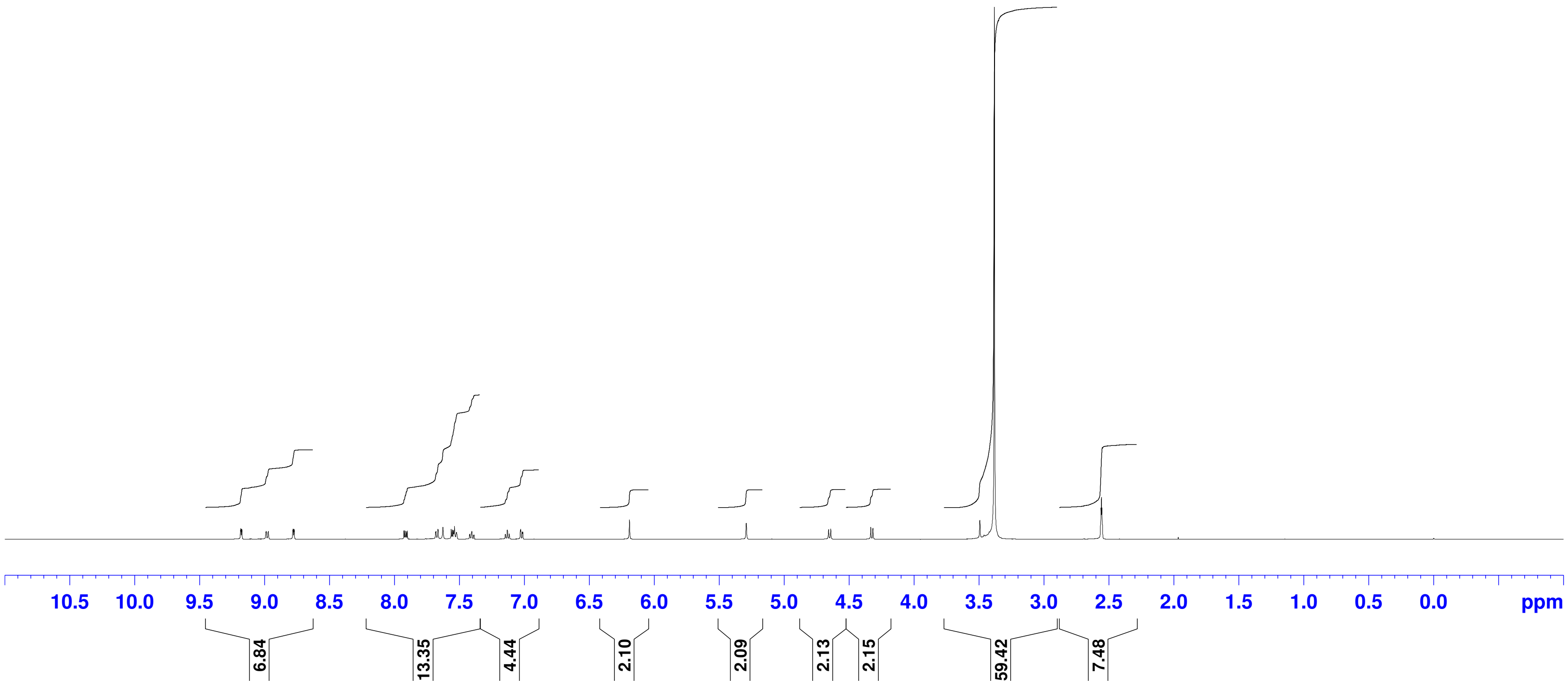

CARDIFF UNIVERSITY
PRIFYSGOL CAERDYDD

NAME	CM-SK82P
EXPNO	6
PROCNO	1
Date_	20141009
Time	15.49
INSTRUM	Avance500
PROBHD	5 mm QNP 1H/13
PULPROG	zg30
TD	65536
SOLVENT	DMSO
NS	16
DS	2
SWH	10330.578 Hz
FIDRES	0.157632 Hz
AQ	3.1719923 sec
RG	362
DW	48.400 use
DE	6.50 use
TE	298.1 K
D1	1.00000000 sec
TD0	1

======== CHANNEL f1 =======

NUC1	1H
P1	11.50 use
PL1	-1.00 dB
PL1W	11.38419914 W
SFO1	500.1330885 MHz
SI	32768
SF	500.1299759 MHz
WDW	EM
SSB	0
LB	0.30 Hz
GB	0
PC	1.00

8b

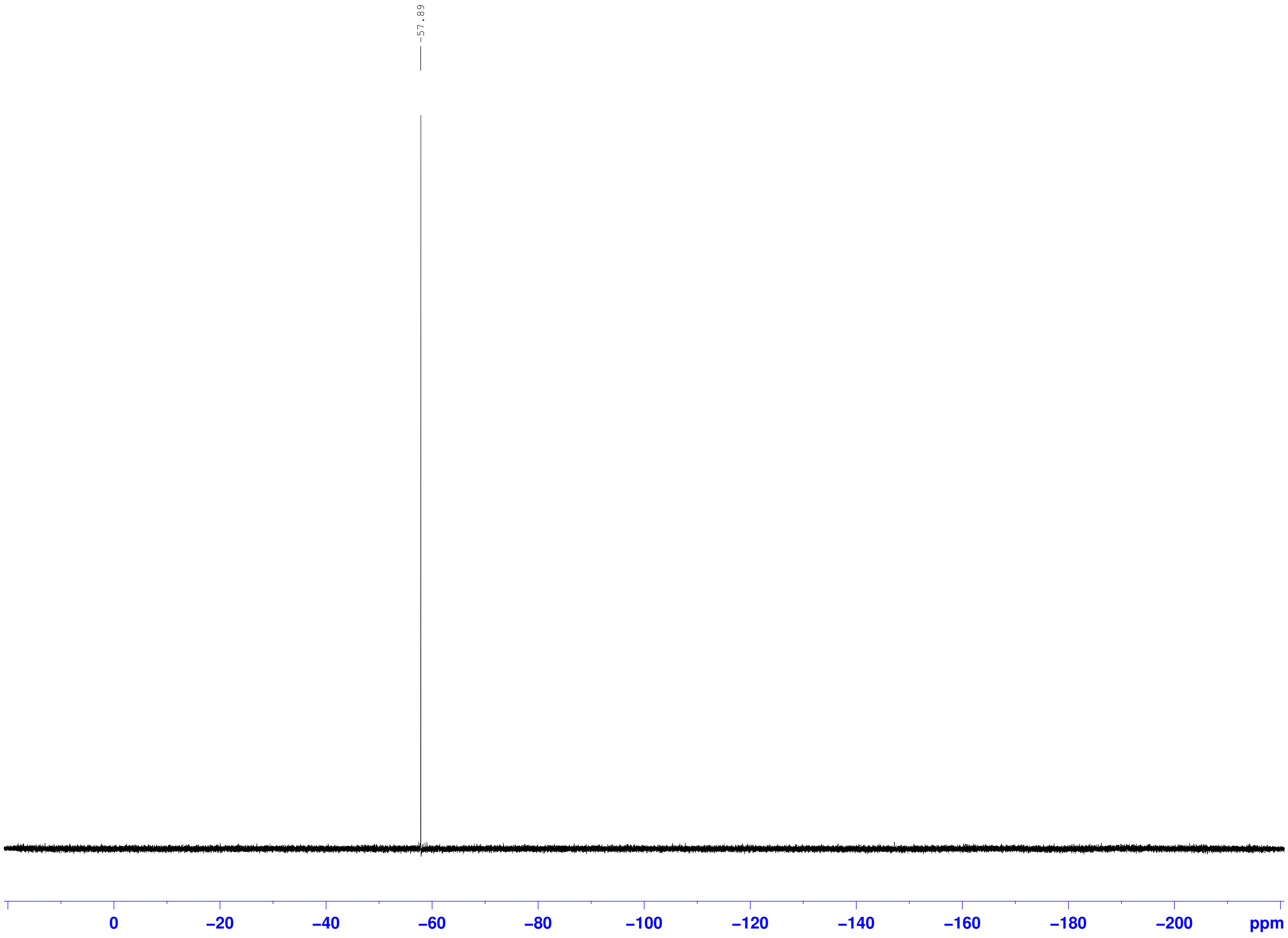

NAME	CM-SK82P
EXPNO	2
PROCNO	1
Date_	20140923
Time	9.17
INSTRUM	Avance500
PROBHD	5 mm QNP 1H/13
PULPROG	zgfhigqn
TD	131072
SOLVENT	MeOD
NS	16
DS	4
SWH	113636.367 Hz
FIDRES	0.866977 Hz
AQ	0.5767668 sec
RG	4100
DW	4.400 use
DE	6.00 use
TE	288.8 K
D1	1.00000000 sec
D11	0.03000000 sec
D12	0.00002000 sec
TD0	1
======== CHANNEL f1 =======	
NUC1	19F
P1	18.60 use
PL1	-1.50 dB
PL1W	11.14113998 W
SFO1	470.5453180 MHz
======== CHANNEL f2 =======	
CPDPRG2	waltz16
NUC2	1H
PCPD2	80.00 use
PL2	-2.00 dB
PL12	14.85 dB
PL2W	14.33185768 W
PL12W	0.29600734 W
SFO2	500.1320005 MHz
SI	65536
SF	470.5923770 MHz
WDW	EM
SSB	0
LB	0.30 Hz
GB	0
PC	1.40

8b

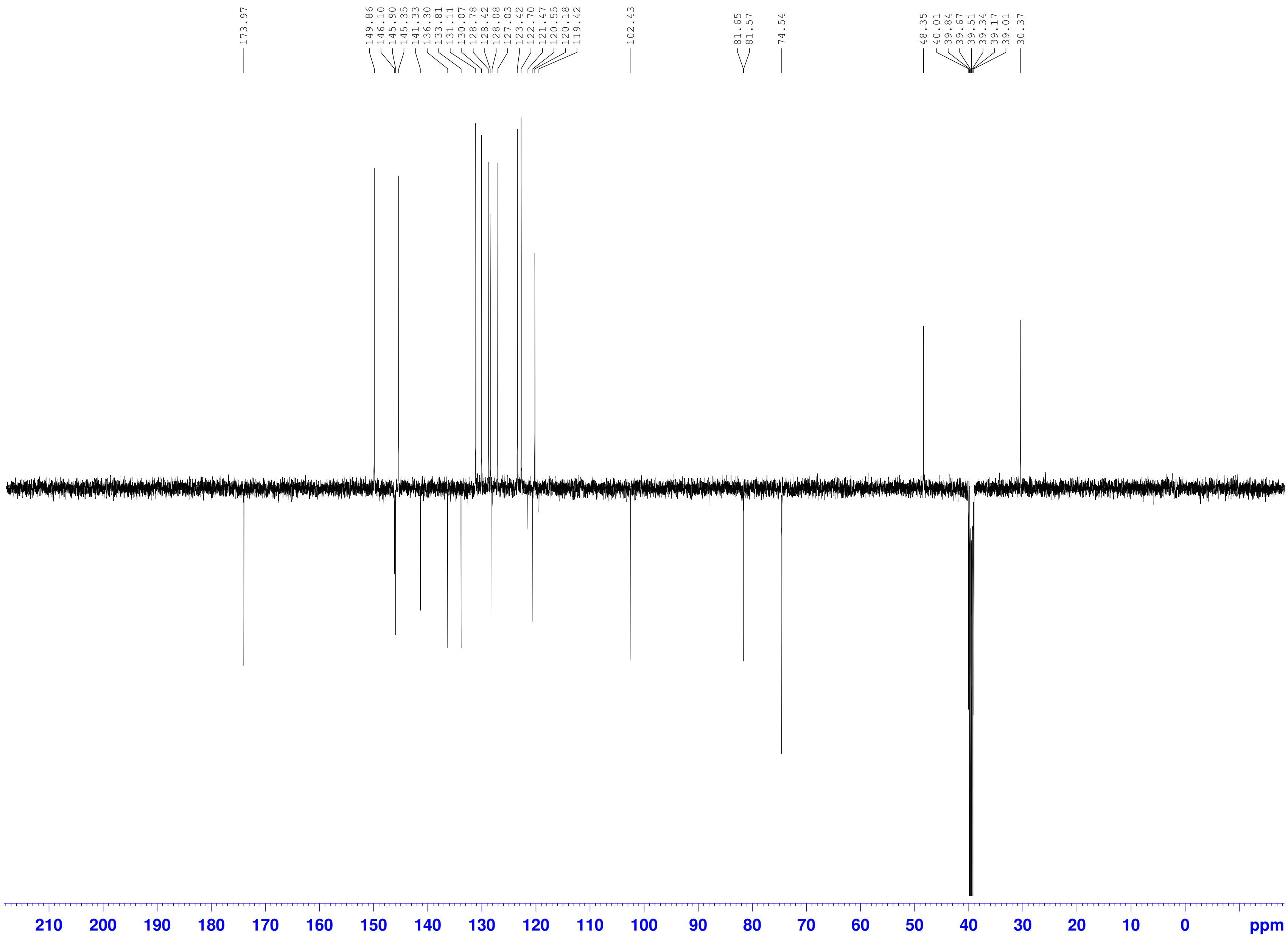

CARDIFF UNIVERSITY
PRIFYSGOL CAERDYDD

NAME	CM-SK82P
EXPNO	8
PROCNO	1
Date_	20141010
Time	19.22
INSTRUM	Avance500
PROBHD	5 mm QNP 1H/13
PULPROG	pendant
TD	65536
SOLVENT	DMSO
NS	3200
DS	4
SWH	29761.904 Hz
FIDRES	0.454131 Hz
AQ	1.1010548 sec
RG	3250
DW	16.800 usec
DE	12.00 usec
TE	298.1 K
CNST2	145.0000000
D1	2.00000000 sec
D4	0.00172414 sec
D12	0.00002000 sec
D15	0.00431034 sec
D20	0.00345000 sec
TD0	25

======== CHANNEL f1 ========

NUC1	13C
P1	7.20 usec
P2	14.40 usec
PL1	-2.00 dB
PL1W	101.27846527 W
SFO1	125.7703643 MHz

======== CHANNEL f2 ========

CPDPRG2	waltz16
NUC2	1H
P3	11.50 usec
P4	23.00 usec
PCPD2	80.00 usec
PL2	-2.00 dB
PL12	14.85 dB
PL2W	14.33185768 W
PL12W	0.29600734 W
SFO2	500.1320005 MHz
SI	32768
SF	125.7578519 MHz
WDW	EM
SSB	0
LB	1.00 Hz
GB	0
PC	1.40

SK82P MW=467?
(MeOH)/MeOH + NH4OAc
C24H16F3N3O4

EPSRC National Facility Swansea
LTQ Orbitrap XL

Dr AD Westwell
20/07/2015 09:26:09

8b

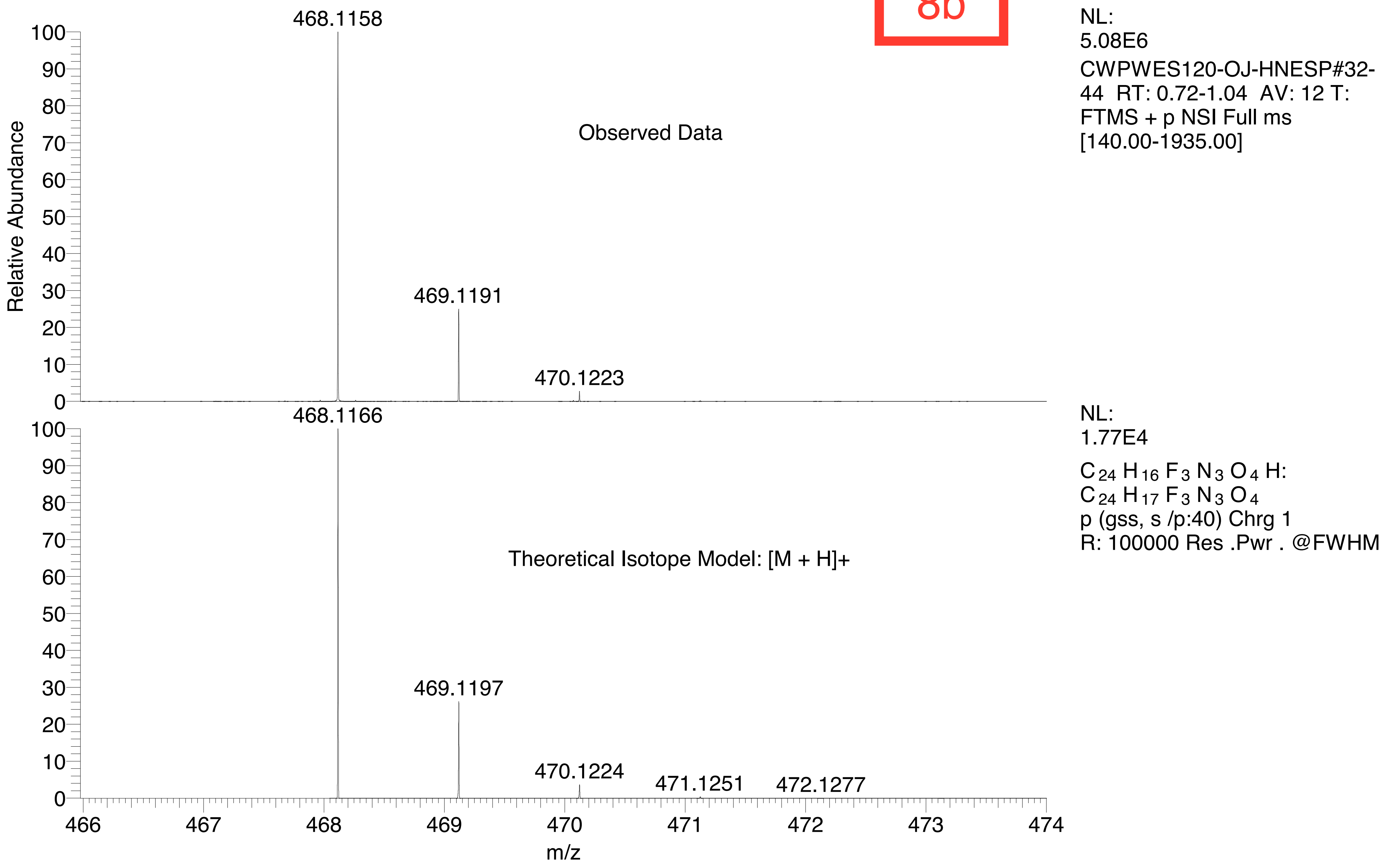

8c

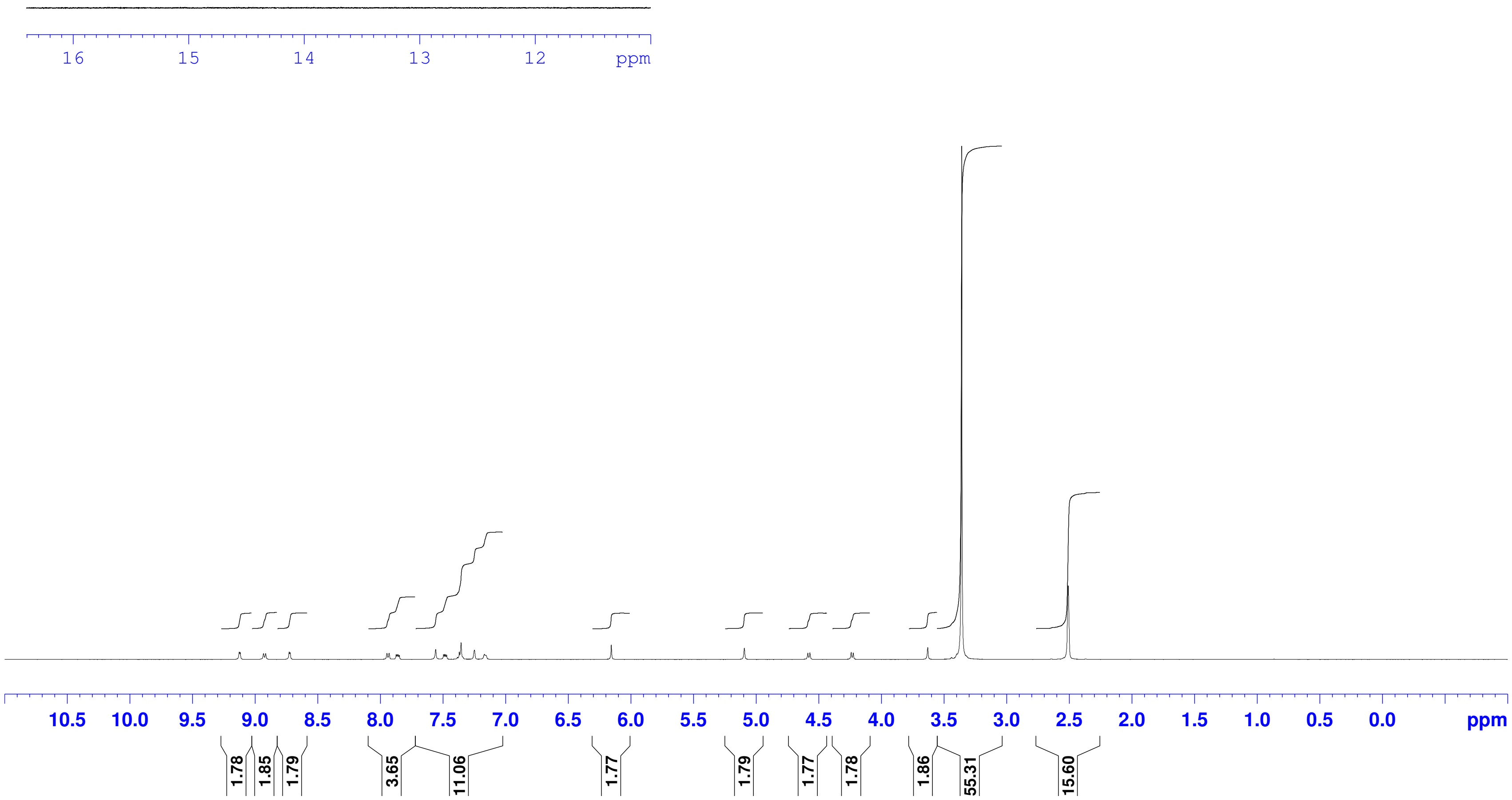

NAME	CM-SK83P
EXPNO	4
PROCNO	1
Date_	20141001
Time	12.16
INSTRUM	Avance500
PROBHD	5 mm QNP 1H/13
PULPROG	zg30
TD	65536
SOLVENT	DMSO
NS	16
DS	2
SWH	10330.578 Hz
FIDRES	0.157632 Hz
AQ	3.1719923 sec
RG	645
DW	48.400 use
DE	6.50 use
TE	289.6 K
D1	1.00000000 sec
TD0	1

======== CHANNEL f1 =======

NUC1	1H
P1	11.50 use
PL1	-1.00 dB
PL1W	11.38419914 W
SFO1	500.1330885 MHz
SI	32768
SF	500.1300000 MHz
WDW	EM
SSB	0
LB	0.30 Hz
GB	0
PC	1.00

8c

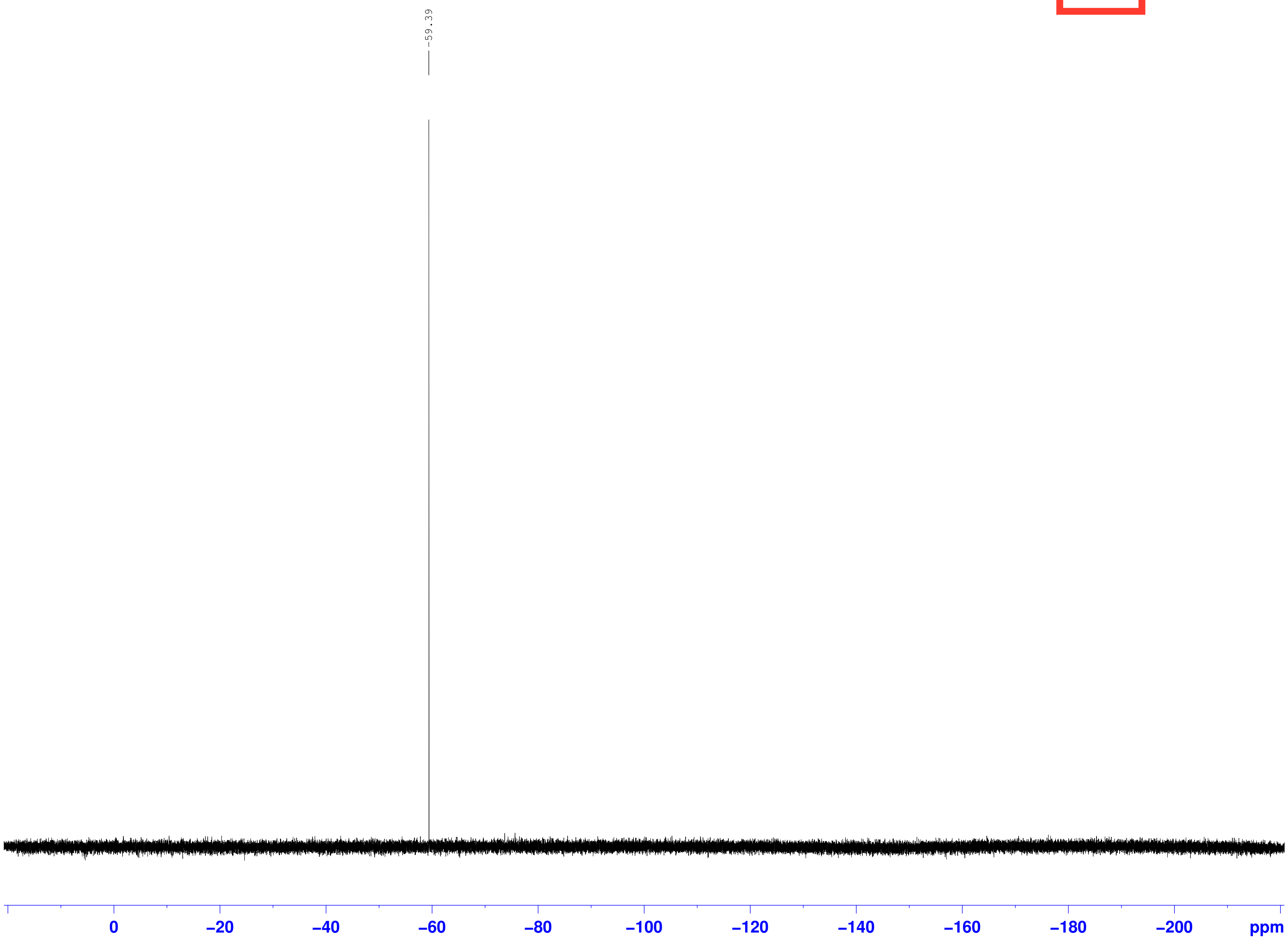

NAME	CM-SK83P
EXPNO	3
PROCNO	1
Date_	20141001
Time	11.24
INSTRUM	Avance500
PROBHD	5 mm QNP 1H/13
PULPROG	zgfhigqn
TD	131072
SOLVENT	MeOD
NS	16
DS	4
SWH	113636.367 Hz
FIDRES	0.866977 Hz
AQ	0.5767668 sec
RG	4100
DW	4.400 use
DE	6.00 use
TE	289.8 K
D1	1.00000000 sec
D11	0.03000000 sec
D12	0.00002000 sec
TD0	1

======== CHANNEL f1 =======

NUC1	19F
P1	18.60 use
PL1	-1.50 dB
PL1W	11.14113998 W
SFO1	470.5453180 MHz

======== CHANNEL f2 =======

CPDPRG2	waltz16
NUC2	1H
PCPD2	80.00 use
PL2	-2.00 dB
PL12	14.85 dB
PL2W	14.33185768 W
PL12W	0.29600734 W
SFO2	500.1320005 MHz
SI	65536
SF	470.5923770 MHz
WDW	EM
SSB	0
LB	0.30 Hz
GB	0
PC	1.40

8c

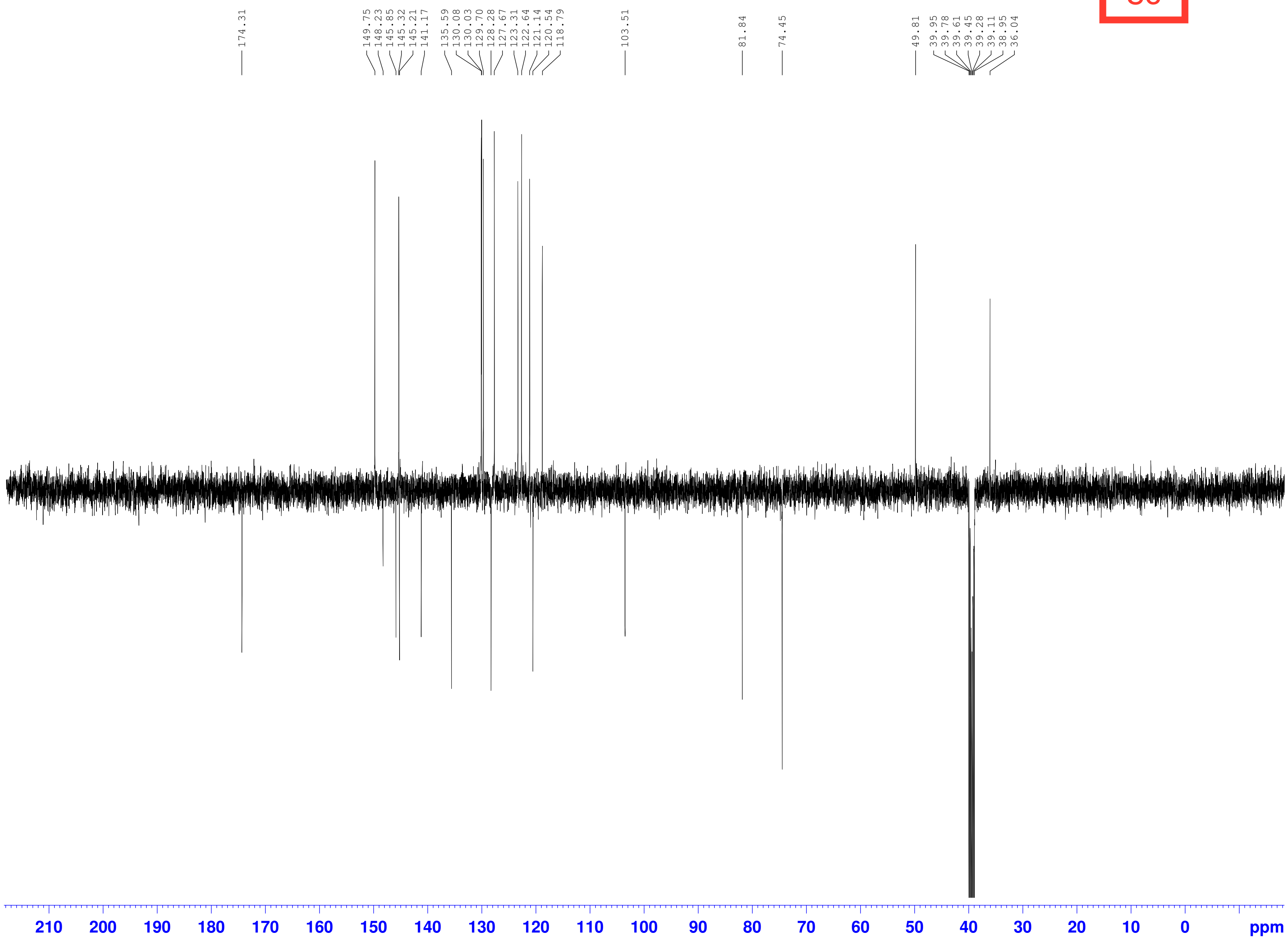

NAME	CM-SK83P
EXPNO	8
PROCNO	1
Date_	20141001
Time	21.59
INSTRUM	Avance500
PROBHD	5 mm QNP 1H/13
PULPROG	pendant
TD	65536
SOLVENT	DMSO
NS	2560
DS	4
SWH	29761.904 Hz
FIDRES	0.454131 Hz
AQ	1.1010548 sec
RG	3250
DW	16.800 usec
DE	12.00 usec
TE	290.0 K
CNST2	145.0000000
D1	2.00000000 sec
D4	0.00172414 sec
D12	0.00002000 sec
D15	0.00431034 sec
D20	0.00345000 sec
TD0	20
======== CHANNEL f1 ========	
NUC1	13C
P1	7.20 usec
P2	14.40 usec
PL1	-2.00 dB
PL1W	101.27846527 W
SFO1	125.7703643 MHz
======== CHANNEL f2 ========	
CPDPRG2	waltz16
NUC2	1H
P3	11.50 usec
P4	23.00 usec
PCPD2	80.00 usec
PL2	-2.00 dB
PL12	14.85 dB
PL2W	14.33185768 W
PL12W	0.29600734 W
SFO2	500.1320005 MHz
SI	32768
SF	125.7578519 MHz
WDW	EM
SSB	0
LB	1.00 Hz
GB	0
PC	1.40

SK83P MW=467?
(MeOH)/MeOH + NH4OAc
C24H16F3N3O4

EPSRC National Facility Swansea
LTQ Orbitrap XL

Dr AD Westwell
20/07/2015 09:23:17

8c

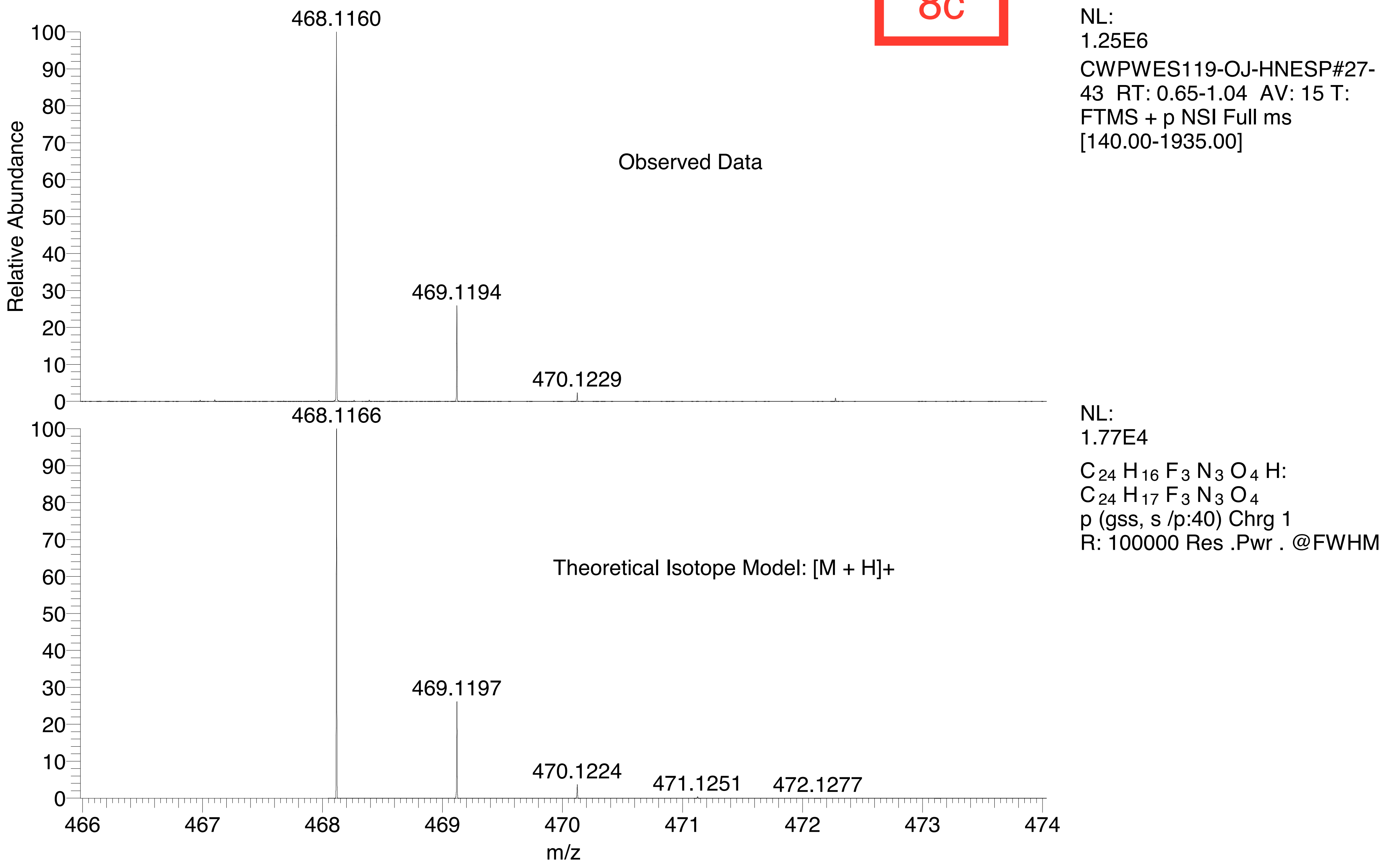

8d

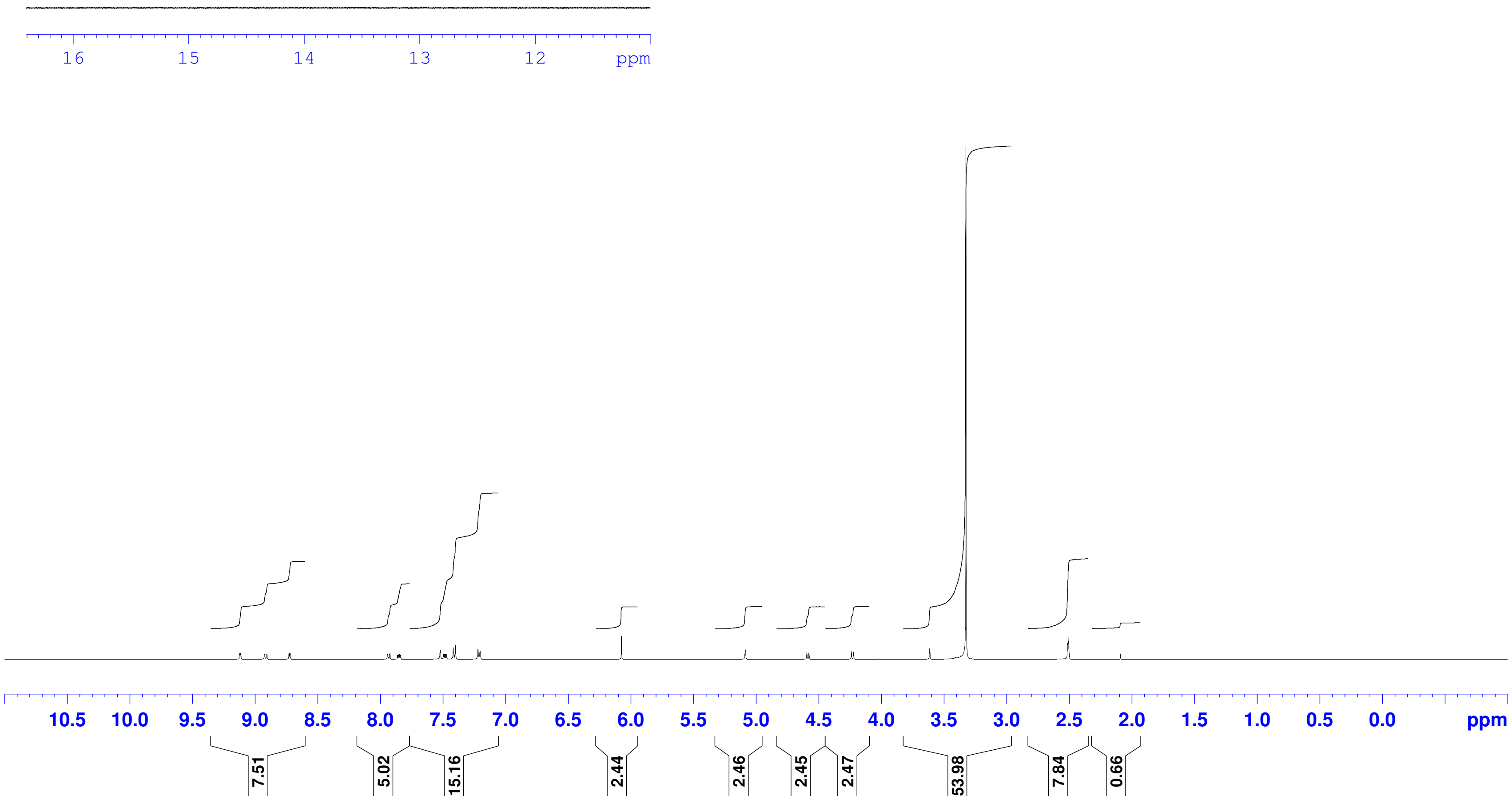

NAME	CM-SK84P
EXPNO	11
PROCNO	1
Date_	20141008
Time	0.04
INSTRUM	Avance500
PROBHD	5 mm QNP 1H/13
PULPROG	zg30
TD	65536
SOLVENT	DMSO
NS	16
DS	2
SWH	10330.578 Hz
FIDRES	0.157632 Hz
AQ	3.1719923 sec
RG	645
DW	48.400 use
DE	6.50 use
TE	298.1 K
D1	1.00000000 sec
TD0	1

======== CHANNEL f1 =======

NUC1	1H
P1	11.50 use
PL1	-1.00 dB
PL1W	11.38419914 W
SFO1	500.1330885 MHz
SI	32768
SF	500.1300000 MHz
WDW	EM
SSB	0
LB	0.30 Hz
GB	0
PC	1.00

8d

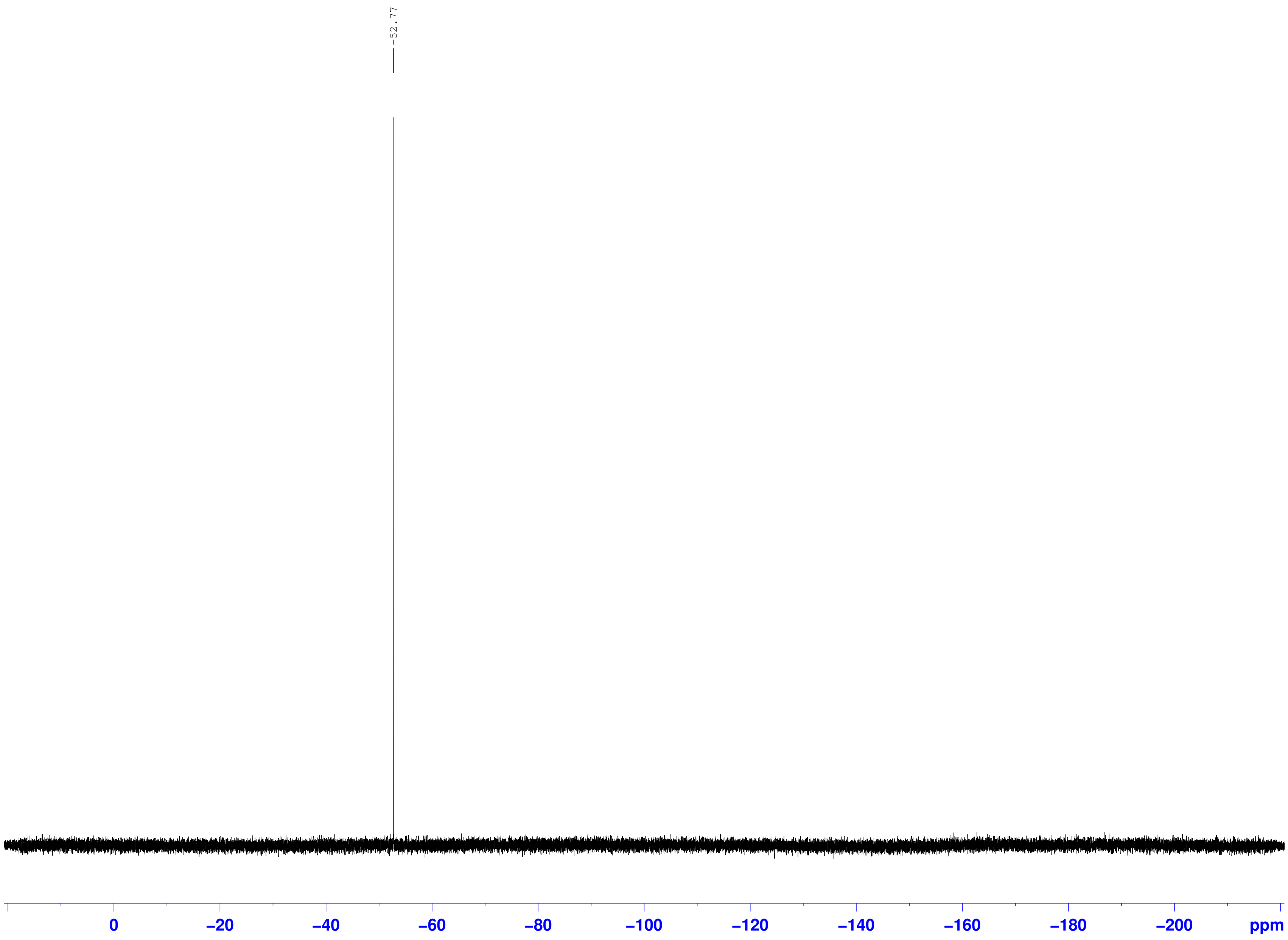

NAME	CM-SK84P
EXPNO	4
PROCNO	1
Date_	20141003
Time	13.19
INSTRUM	Avance500
PROBHD	5 mm QNP 1H/13
PULPROG	zgfhigqn
TD	131072
SOLVENT	CDCl3
NS	16
DS	4
SWH	113636.367 Hz
FIDRES	0.866977 Hz
AQ	0.5767668 sec
RG	4100
DW	4.400 use
DE	6.00 use
TE	289.3 K
D1	1.00000000 sec
D11	0.03000000 sec
D12	0.00002000 sec
TD0	1
======== CHANNEL f1 =======	
NUC1	19F
P1	18.60 use
PL1	-1.50 dB
PL1W	11.14113998 W
SFO1	470.5453180 MHz
======== CHANNEL f2 =======	
CPDPRG2	waltz16
NUC2	1H
PCPD2	80.00 use
PL2	-2.00 dB
PL12	14.85 dB
PL2W	14.33185768 W
PL12W	0.29600734 W
SFO2	500.1320005 MHz
SI	65536
SF	470.5923770 MHz
WDW	EM
SSB	0
LB	0.30 Hz
GB	0
PC	1.40

8d

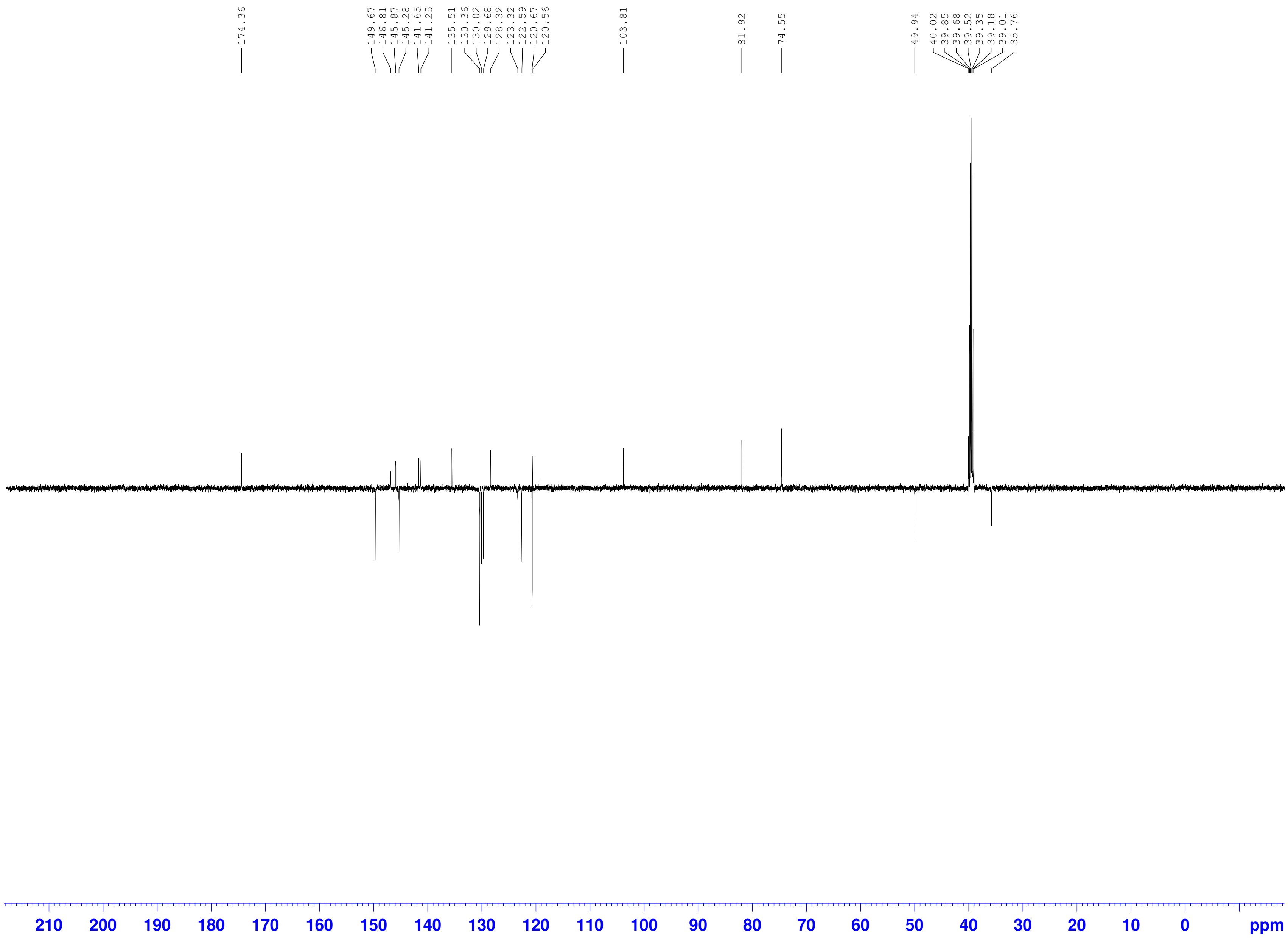

NAME	CM-SK84P
EXPNO	10
PROCNO	1
Date_	20141007
Time	22.49
INSTRUM	Avance500
PROBHD	5 mm QNP 1H/13
PULPROG	pendant
TD	65536
SOLVENT	DMSO
NS	1536
DS	4
SWH	29761.904 Hz
FIDRES	0.454131 Hz
AQ	1.1010548 sec
RG	3250
DW	16.800 usec
DE	12.00 usec
TE	298.1 K
CNST2	145.0000000
D1	2.00000000 sec
D4	0.00172414 sec
D12	0.00002000 sec
D15	0.00431034 sec
D20	0.00345000 sec
TD0	12
======== CHANNEL f1 ========	
NUC1	13C
P1	7.20 usec
P2	14.40 usec
PL1	-2.00 dB
PL1W	101.27846527 W
SFO1	125.7703643 MHz
======== CHANNEL f2 ========	
CPDPRG2	waltz16
NUC2	1H
P3	11.50 usec
P4	23.00 usec
PCPD2	80.00 usec
PL2	-2.00 dB
PL12	14.85 dB
PL2W	14.33185768 W
PL12W	0.29600734 W
SFO2	500.1320005 MHz
SI	32768
SF	125.7578519 MHz
WDW	EM
SSB	0
LB	1.00 Hz
GB	0
PC	1.40

SK84P MW=467?
(MeOH)/MeOH + NH4OAc
C24H16F3N3O4

EPSRC National Facility Swansea
LTQ Orbitrap XL

Dr AD Westwell
20/07/2015 09:20:26

8d

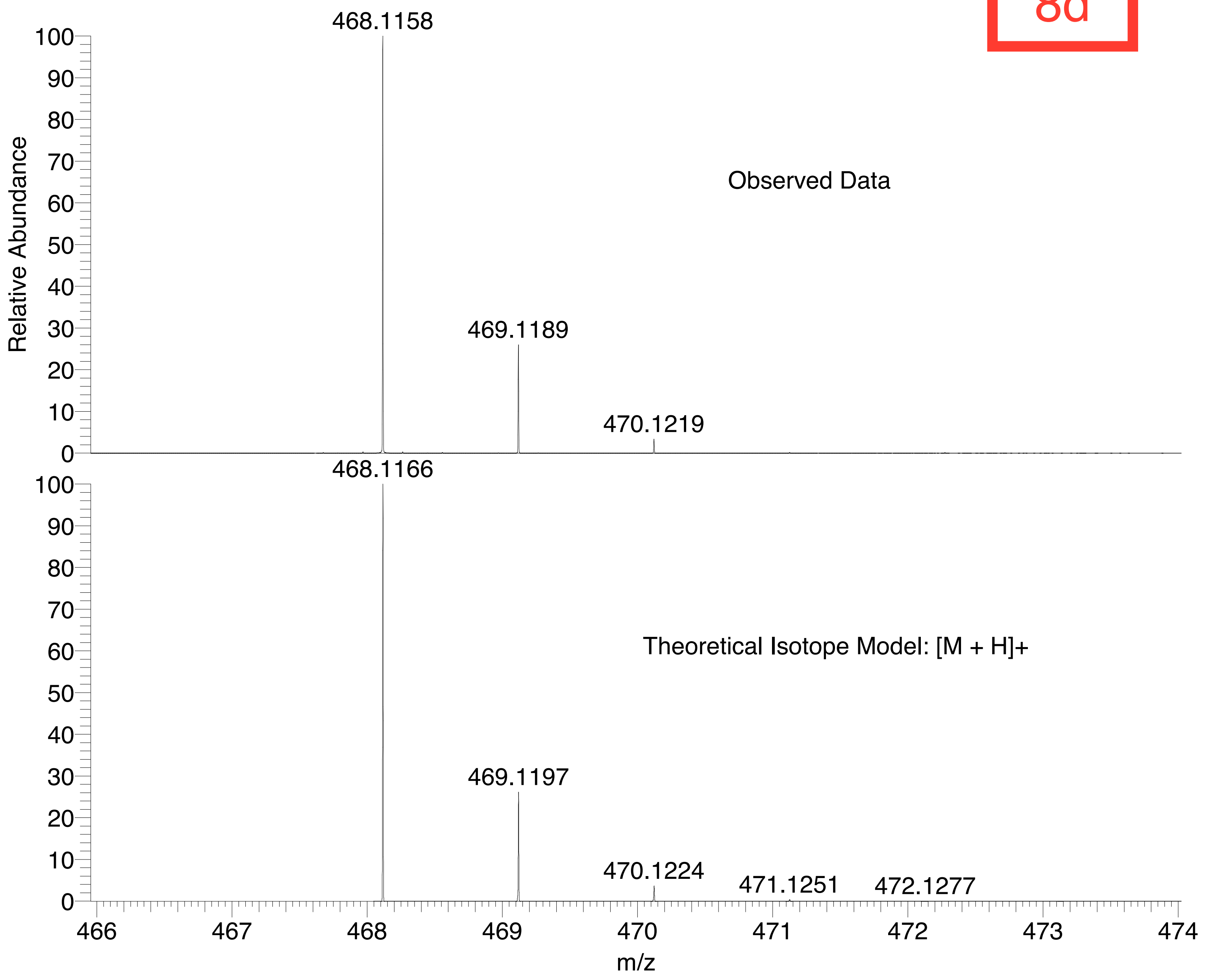

NL:
8.17E6
CWPWES118-OJ-HNESP#31-44 RT: 0.72-1.04 AV: 12 T: FTMS + p NSI Full ms [140.00-1935.00]

NL:
1.77E4
$C_{24}H_{16}F_3N_3O_4H$:
$C_{24}H_{17}F_3N_3O_4$
p (gss, s /p:40) Chrg 1
R: 100000 Res .Pwr . @FWHM

8e

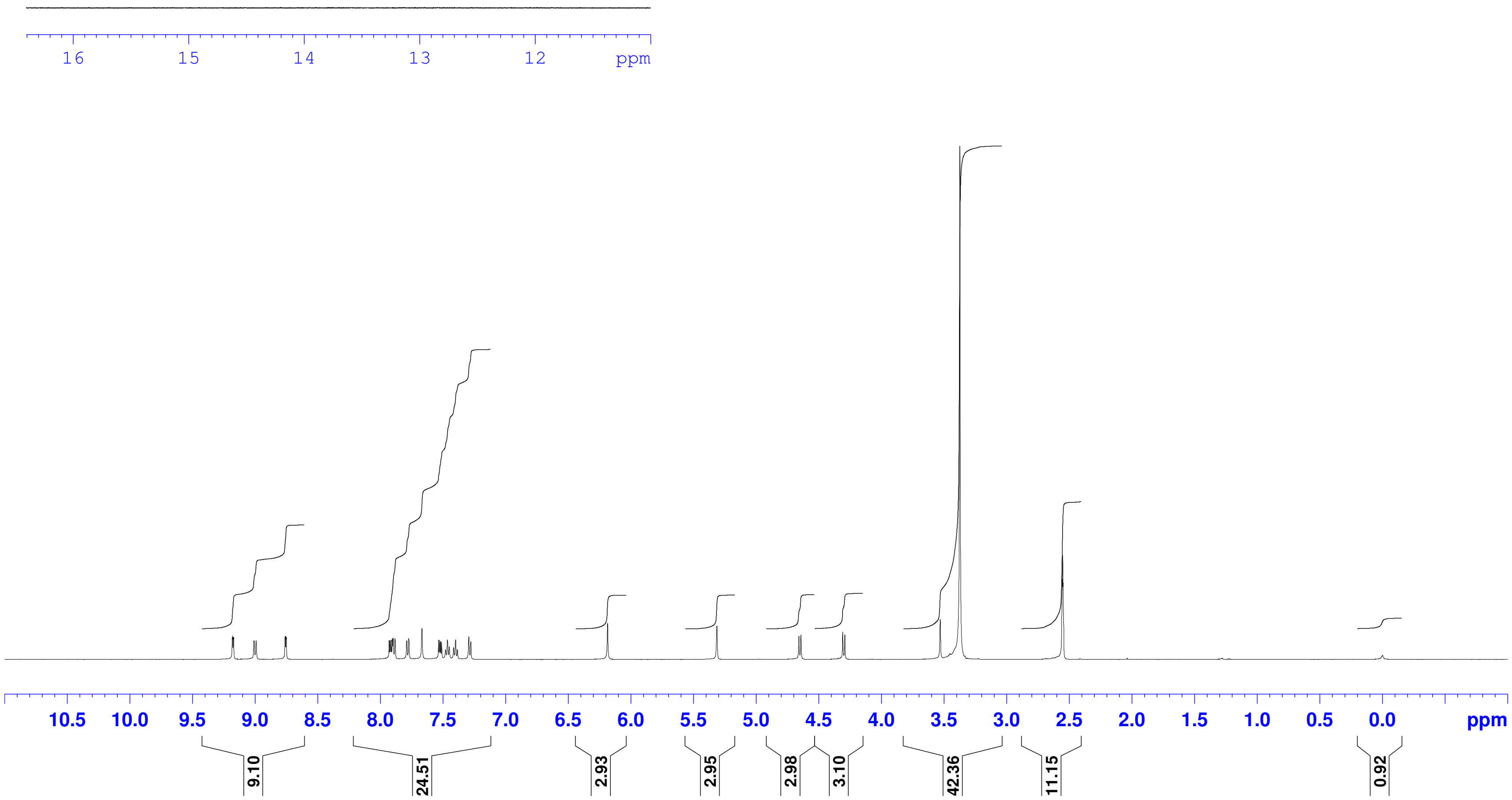

NAME	CM-SK85P
EXPNO	5
PROCNO	1
Date_	20141016
Time	11.13
INSTRUM	Avance500
PROBHD	5 mm QNP 1H/13
PULPROG	zg30
TD	65536
SOLVENT	DMSO
NS	16
DS	2
SWH	10330.578 Hz
FIDRES	0.157632 Hz
AQ	3.1719923 sec
RG	362
DW	48.400 use
DE	6.50 use
TE	298.1 K
D1	1.00000000 sec
TD0	1

======== CHANNEL f1 =======

NUC1	1H
P1	11.50 use
PL1	-1.00 dB
PL1W	11.38419914 W
SFO1	500.1330885 MHz
SI	32768
SF	500.1299781 MHz
WDW	EM
SSB	0
LB	0.30 Hz
GB	0
PC	1.00

8e

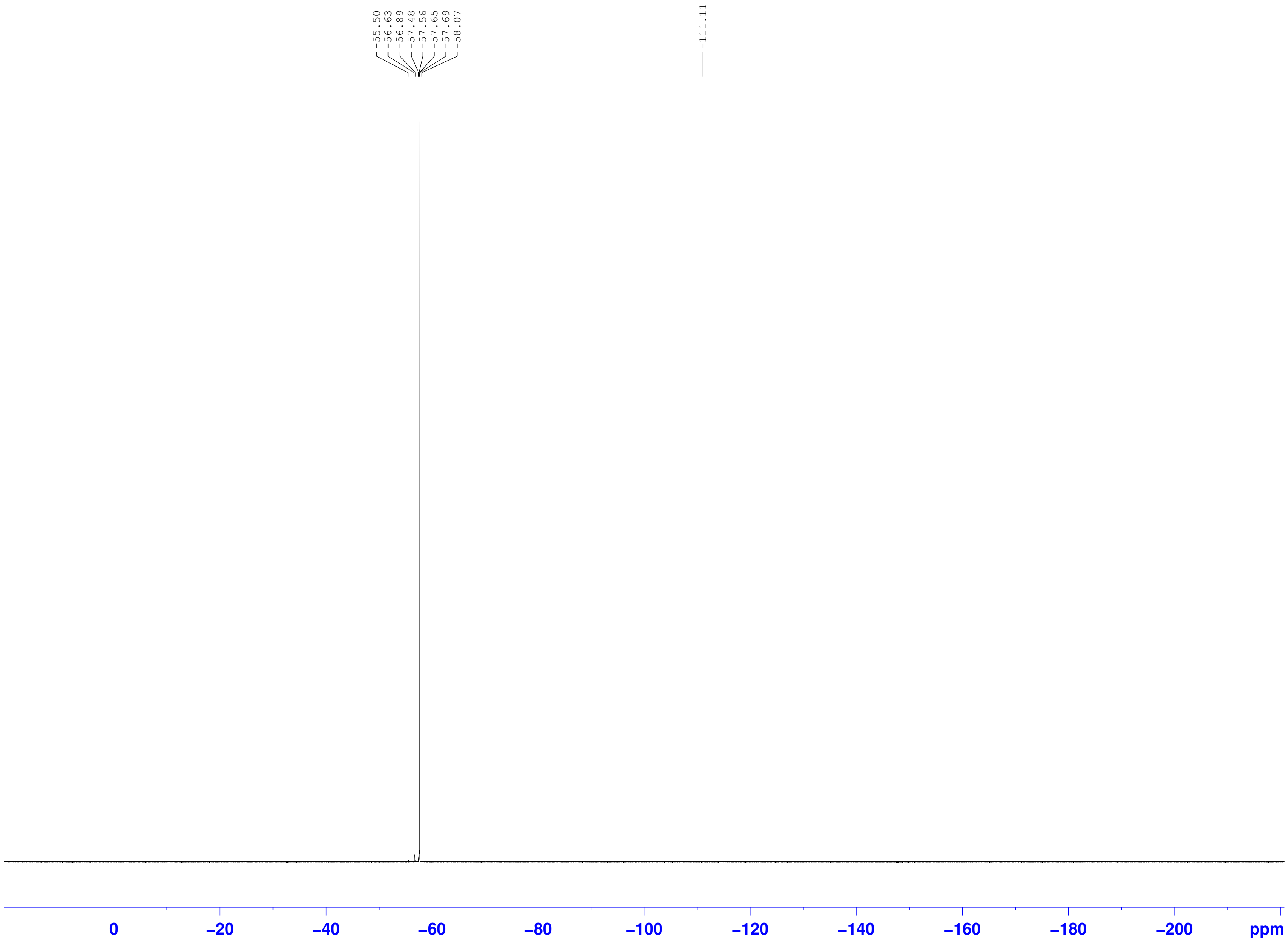

CARDIFF UNIVERSITY
PRIFYSGOL CAERDYDD

NAME	CM-SK85P
EXPNO	4
PROCNO	1
Date_	20141016
Time	11.11
INSTRUM	Avance500
PROBHD	5 mm QNP 1H/13
PULPROG	zgfhigqn
TD	131072
SOLVENT	DMSO
NS	16
DS	4
SWH	113636.367 Hz
FIDRES	0.866977 Hz
AQ	0.5767668 sec
RG	2300
DW	4.400 use
DE	6.00 use
TE	298.1 K
D1	1.00000000 sec
D11	0.03000000 sec
D12	0.00002000 sec
TD0	1

======== CHANNEL f1 =======

NUC1	19F
P1	18.60 use
PL1	-1.50 dB
PL1W	11.14113998 W
SFO1	470.5453180 MHz

======== CHANNEL f2 =======

CPDPRG2	waltz16
NUC2	1H
PCPD2	80.00 use
PL2	-2.00 dB
PL12	14.85 dB
PL2W	14.33185768 W
PL12W	0.29600734 W
SFO2	500.1320005 MHz
SI	65536
SF	470.5923770 MHz
WDW	EM
SSB	0
LB	0.30 Hz
GB	0
PC	1.40

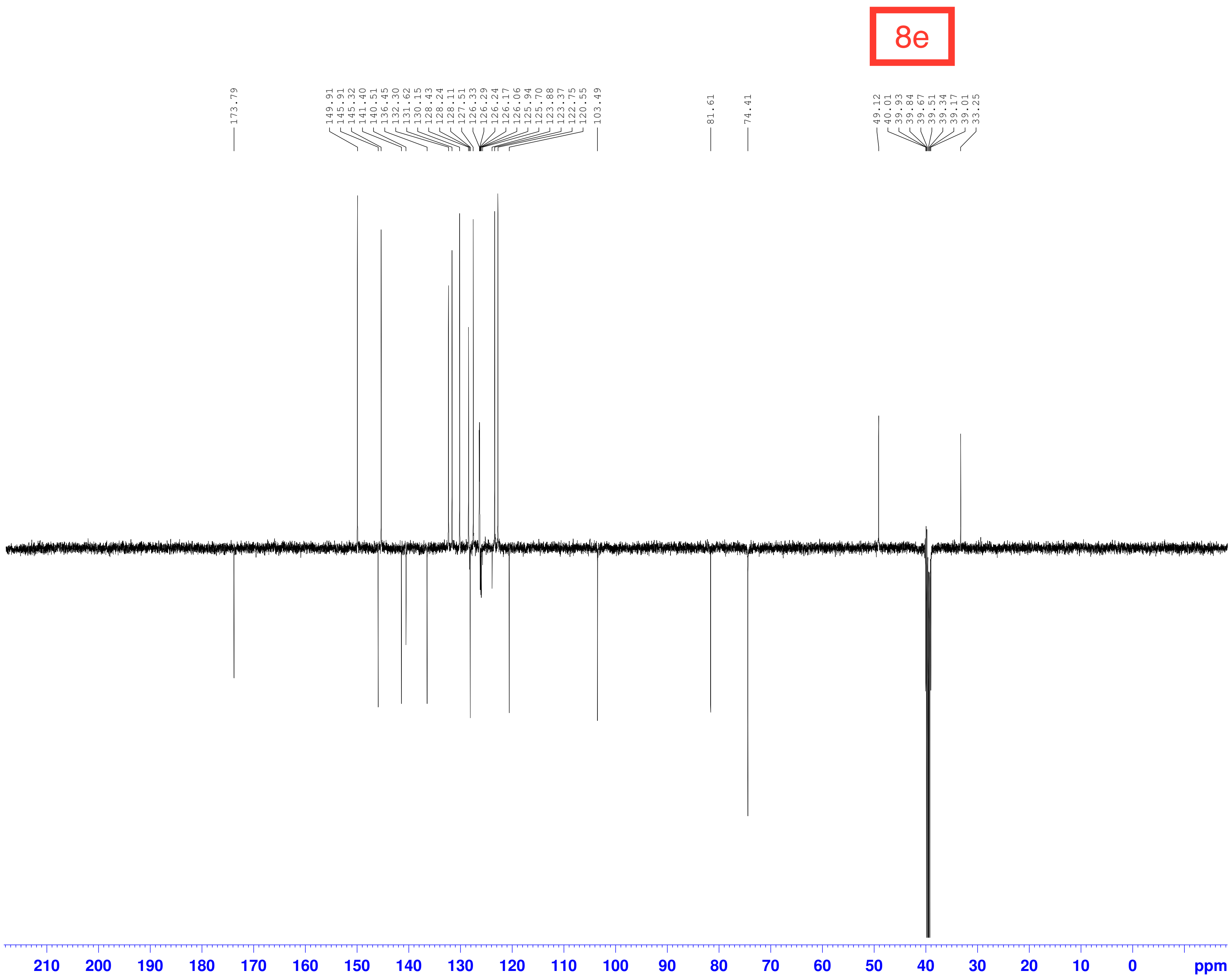

NAME	CM-SK85P
EXPNO	7
PROCNO	1
Date_	20141026
Time	2.36
INSTRUM	Avance500
PROBHD	5 mm QNP 1H/13
PULPROG	pendant
TD	65536
SOLVENT	DMSO
NS	3200
DS	4
SWH	29761.904 Hz
FIDRES	0.454131 Hz
AQ	1.1010548 sec
RG	3250
DW	16.800 usec
DE	12.00 usec
TE	298.1 K
CNST2	145.0000000
D1	2.00000000 sec
D4	0.00172414 sec
D12	0.00002000 sec
D15	0.00431034 sec
D20	0.00345000 sec
TD0	25

======== CHANNEL f1 ========

NUC1	13C
P1	7.20 usec
P2	14.40 usec
PL1	-2.00 dB
PL1W	101.27846527 W
SFO1	125.7703643 MHz

======== CHANNEL f2 ========

CPDPRG2	waltz16
NUC2	1H
P3	11.50 usec
P4	23.00 usec
PCPD2	80.00 usec
PL2	-2.00 dB
PL12	14.85 dB
PL2W	14.33185768 W
PL12W	0.29600734 W
SFO2	500.1320005 MHz
SI	32768
SF	125.7578519 MHz
WDW	EM
SSB	0
LB	1.00 Hz
GB	0
PC	1.40

SK85P MW=451?
(MeOH)/MeOH + NH4OAc
C24H16F3N3O3

EPSRC National Facility Swansea
LTQ Orbitrap XL

Dr AD Westwell
20/07/2015 09:17:35

8e

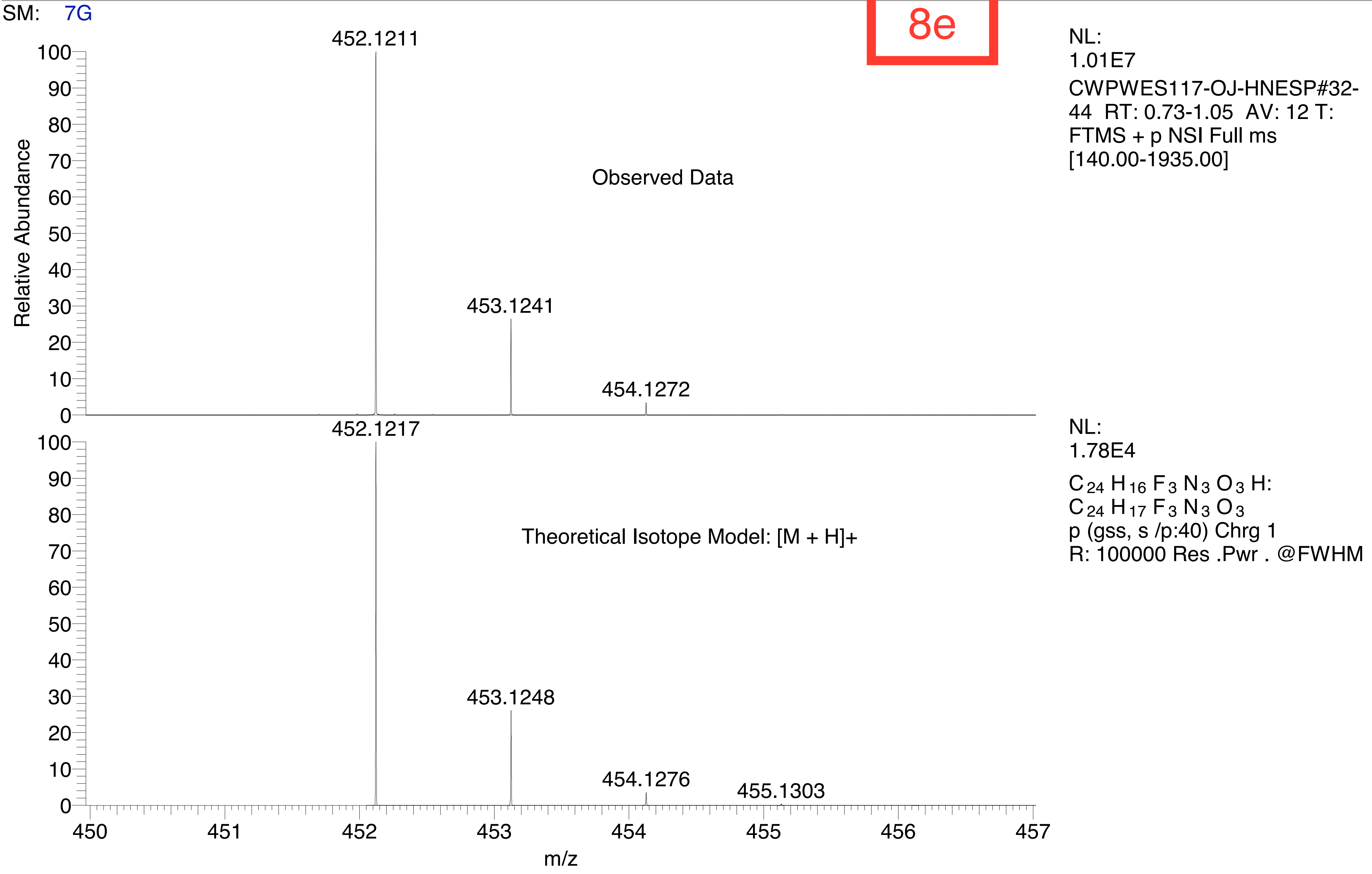

8f

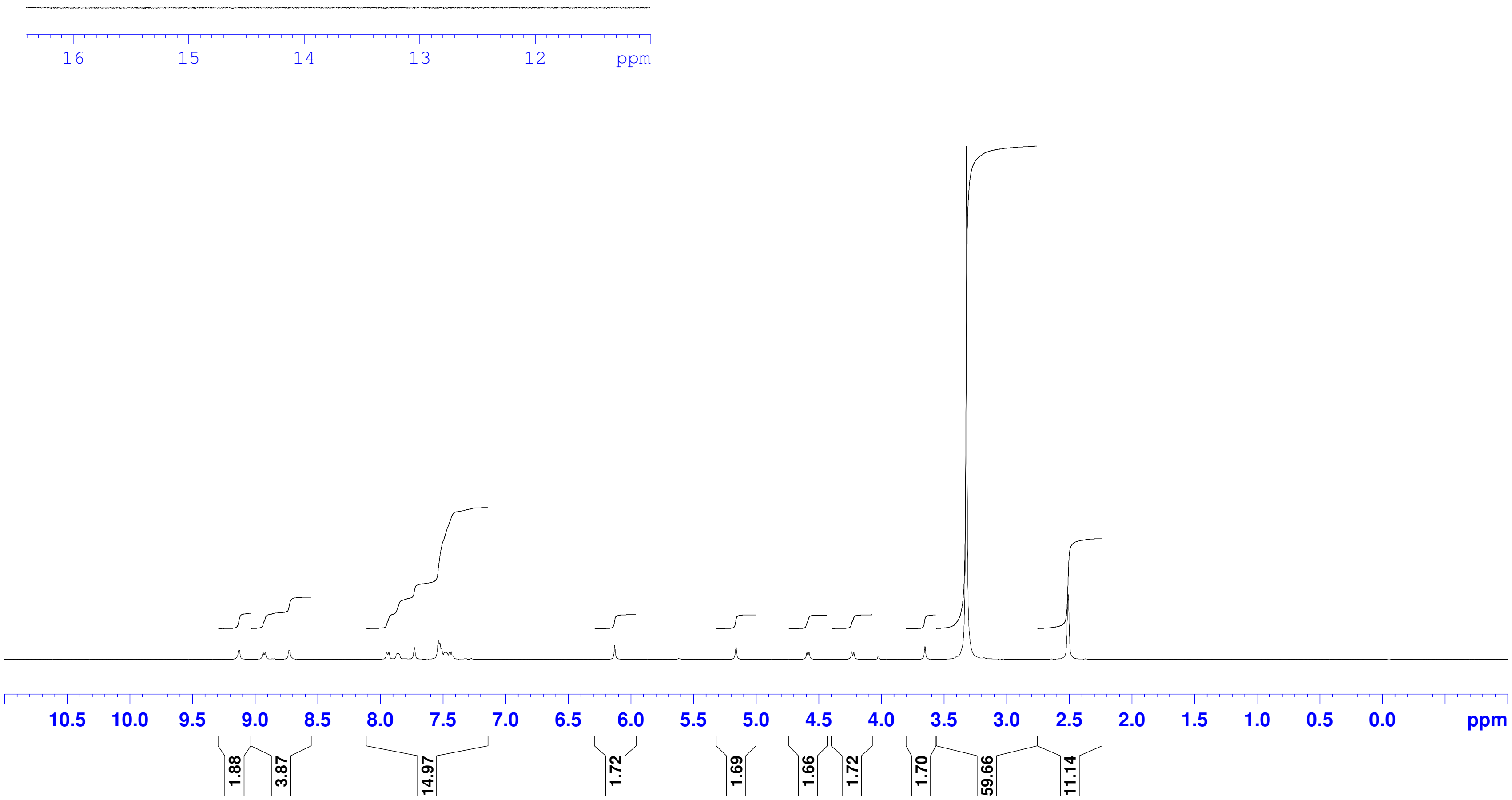

CARDIFF UNIVERSITY
PRIFYSGOL CAERDYDD

NAME	CM-SK86P
EXPNO	5
PROCNO	1
Date_	20141009
Time	10.28
INSTRUM	Avance500
PROBHD	5 mm QNP 1H/13
PULPROG	zg30
TD	65536
SOLVENT	DMSO
NS	16
DS	2
SWH	10330.578 Hz
FIDRES	0.157632 Hz
AQ	3.1719923 sec
RG	645
DW	48.400 use
DE	6.50 use
TE	298.1 K
D1	1.00000000 sec
TD0	1

======== CHANNEL f1 =======

NUC1	1H
P1	11.50 use
PL1	-1.00 dB
PL1W	11.38419914 W
SFO1	500.1330885 MHz
SI	32768
SF	500.1300000 MHz
WDW	EM
SSB	0
LB	0.30 Hz
GB	0
PC	1.00

8f

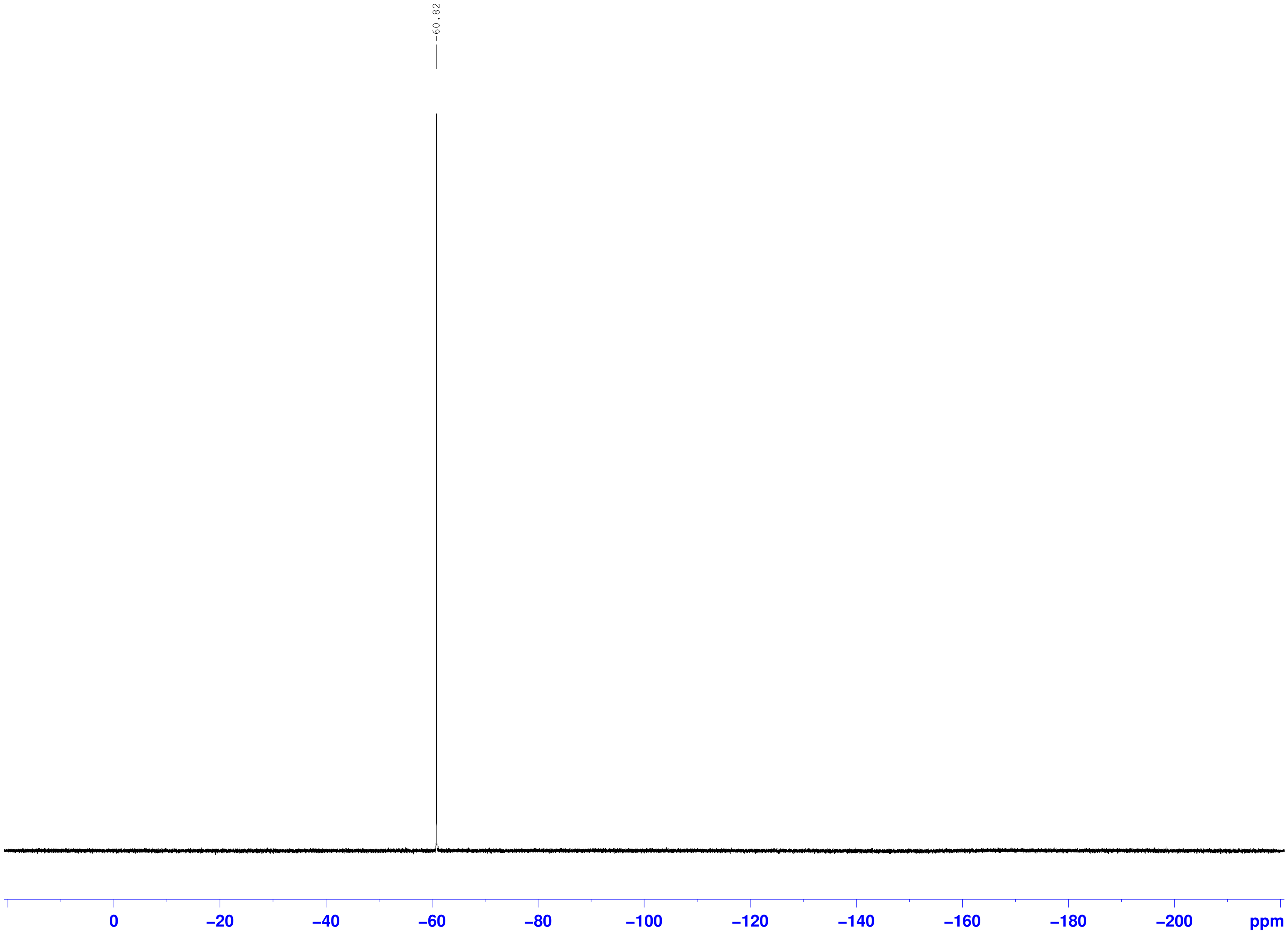

CARDIFF UNIVERSITY PRIFYSGOL CAERDYDD

NAME	CM-SK86P
EXPNO	4
PROCNO	1
Date_	20141009
Time	10.26
INSTRUM	Avance500
PROBHD	5 mm QNP 1H/13
PULPROG	zgfhigqn
TD	131072
SOLVENT	DMSO
NS	16
DS	4
SWH	113636.367 Hz
FIDRES	0.866977 Hz
AQ	0.5767668 sec
RG	2890
DW	4.400 use
DE	6.00 use
TE	298.2 K
D1	1.00000000 sec
D11	0.03000000 sec
D12	0.00002000 sec
TD0	1

======== CHANNEL f1 =======

NUC1	19F
P1	18.60 use
PL1	-1.50 dB
PL1W	11.14113998 W
SFO1	470.5453180 MHz

======== CHANNEL f2 =======

CPDPRG2	waltz16
NUC2	1H
PCPD2	80.00 use
PL2	-2.00 dB
PL12	14.85 dB
PL2W	14.33185768 W
PL12W	0.29600734 W
SFO2	500.1320005 MHz
SI	65536
SF	470.5923770 MHz
WDW	EM
SSB	0
LB	0.30 Hz
GB	0
PC	1.40

8f

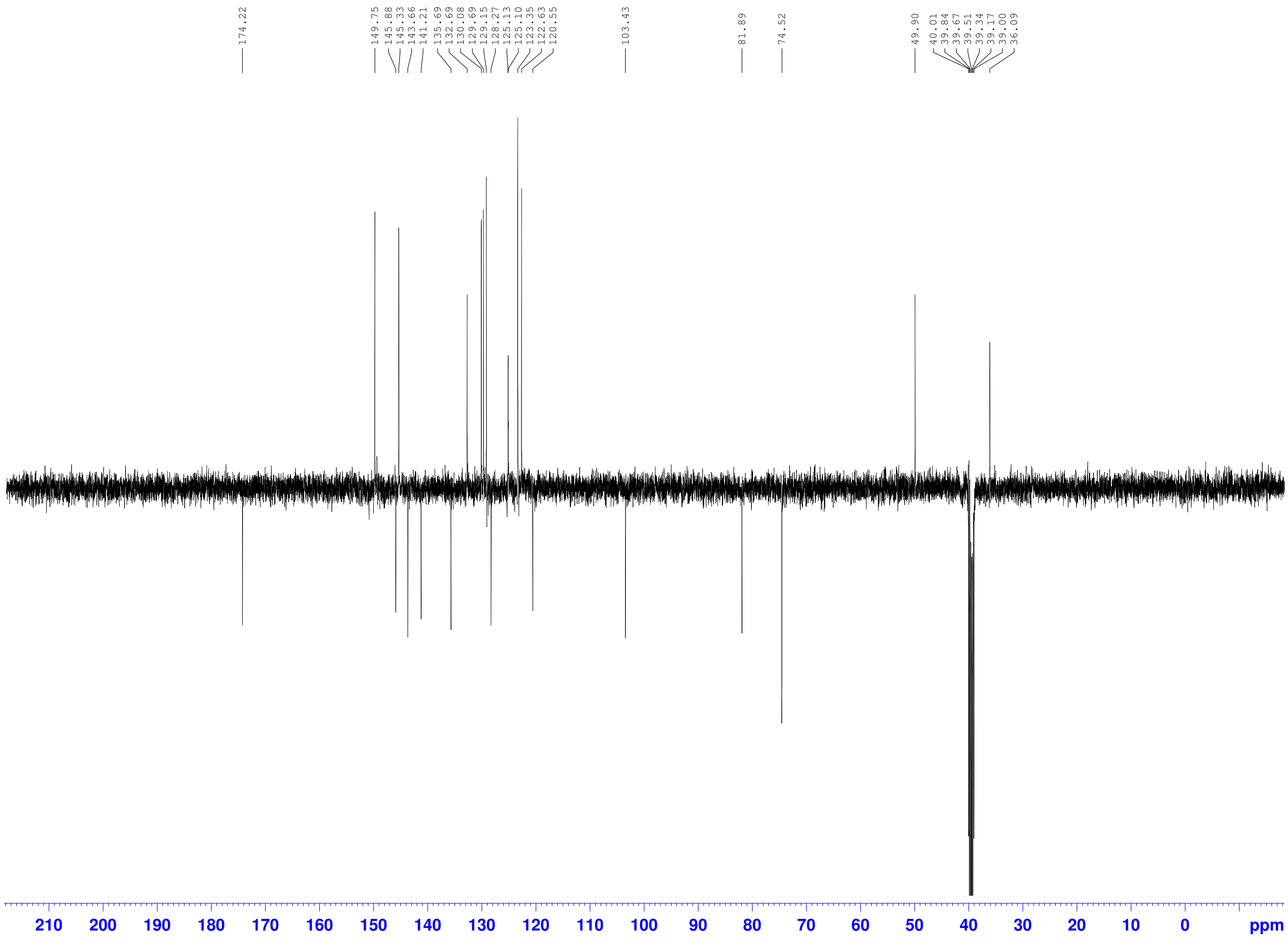

NAME	CM-SK86P	
EXPNO	7	
PROCNO	1	
Date_	20141104	
Time	2.32	
INSTRUM	Avance500	
PROBHD	5 mm QNP 1H/13	
PULPROG	pendant	
TD	65536	
SOLVENT	DMSO	
NS	2560	
DS	4	
SWH	29761.904	Hz
FIDRES	0.454131	Hz
AQ	1.1010548	sec
RG	3250	
DW	16.800	usec
DE	12.00	usec
TE	298.1	K
CNST2	145.0000000	
D1	2.00000000	sec
D4	0.00172414	sec
D12	0.00002000	sec
D15	0.00431034	sec
D20	0.00345000	sec
TD0	20	
======== CHANNEL f1 ========		
NUC1	13C	
P1	7.20	usec
P2	14.40	usec
PL1	-2.00	dB
PL1W	101.27846527	W
SFO1	125.7703643	MHz
======== CHANNEL f2 ========		
CPDPRG2	waltz16	
NUC2	1H	
P3	11.50	usec
P4	23.00	usec
PCPD2	80.00	usec
PL2	-2.00	dB
PL12	14.85	dB
PL2W	14.33185768	W
PL12W	0.29600734	W
SFO2	500.1320005	MHz
SI	32768	
SF	125.7578519	MHz
WDW	EM	
SSB	0	
LB	1.00	Hz
GB	0	
PC	1.40	

SK86P MW=451?
(MeOH)/MeOH + NH4OAc
C24H16F3N3O3

EPSRC National Facility Swansea
LTQ Orbitrap XL

Dr AD Westwell
28/07/2015 12:59:23

8f

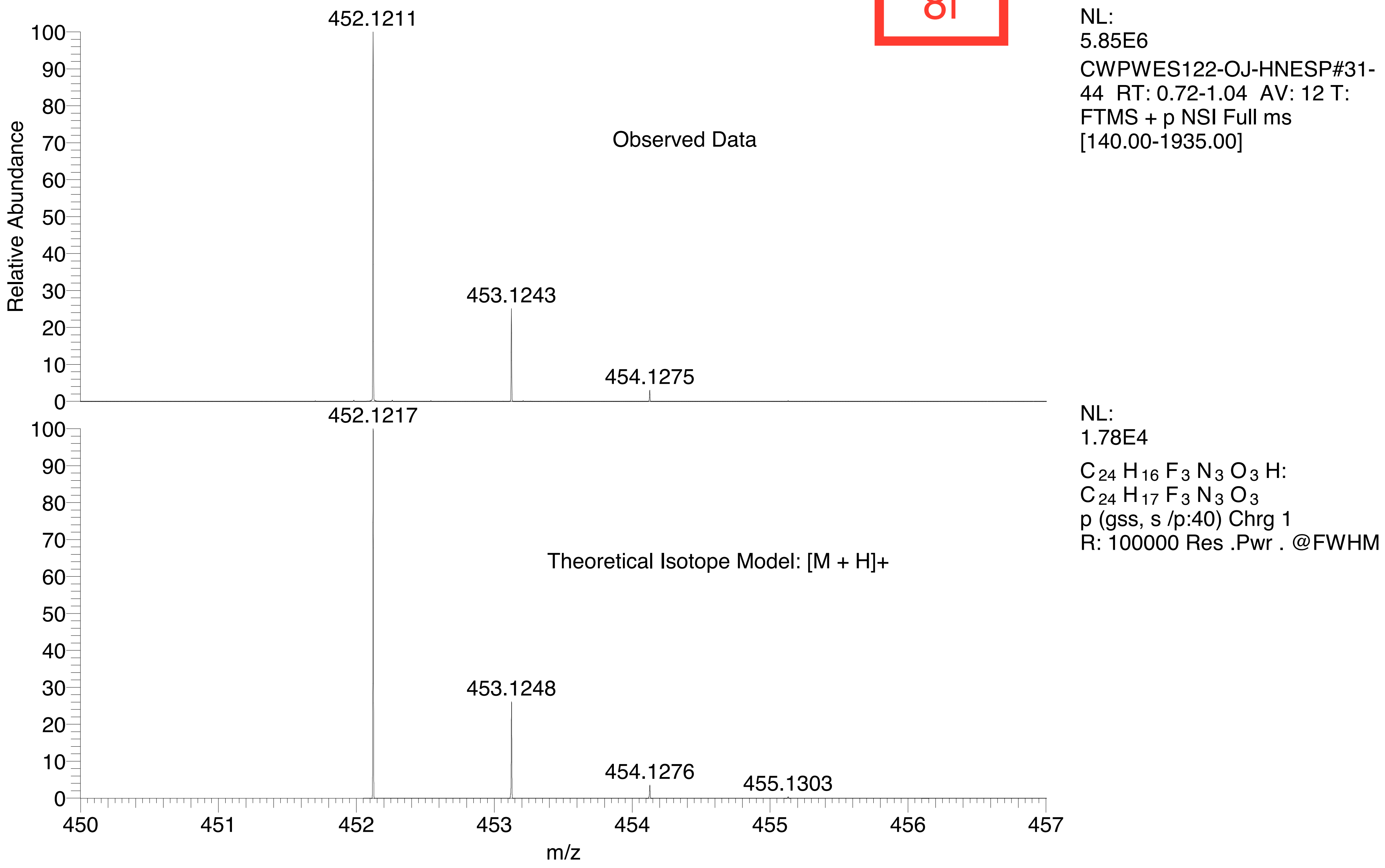

8g

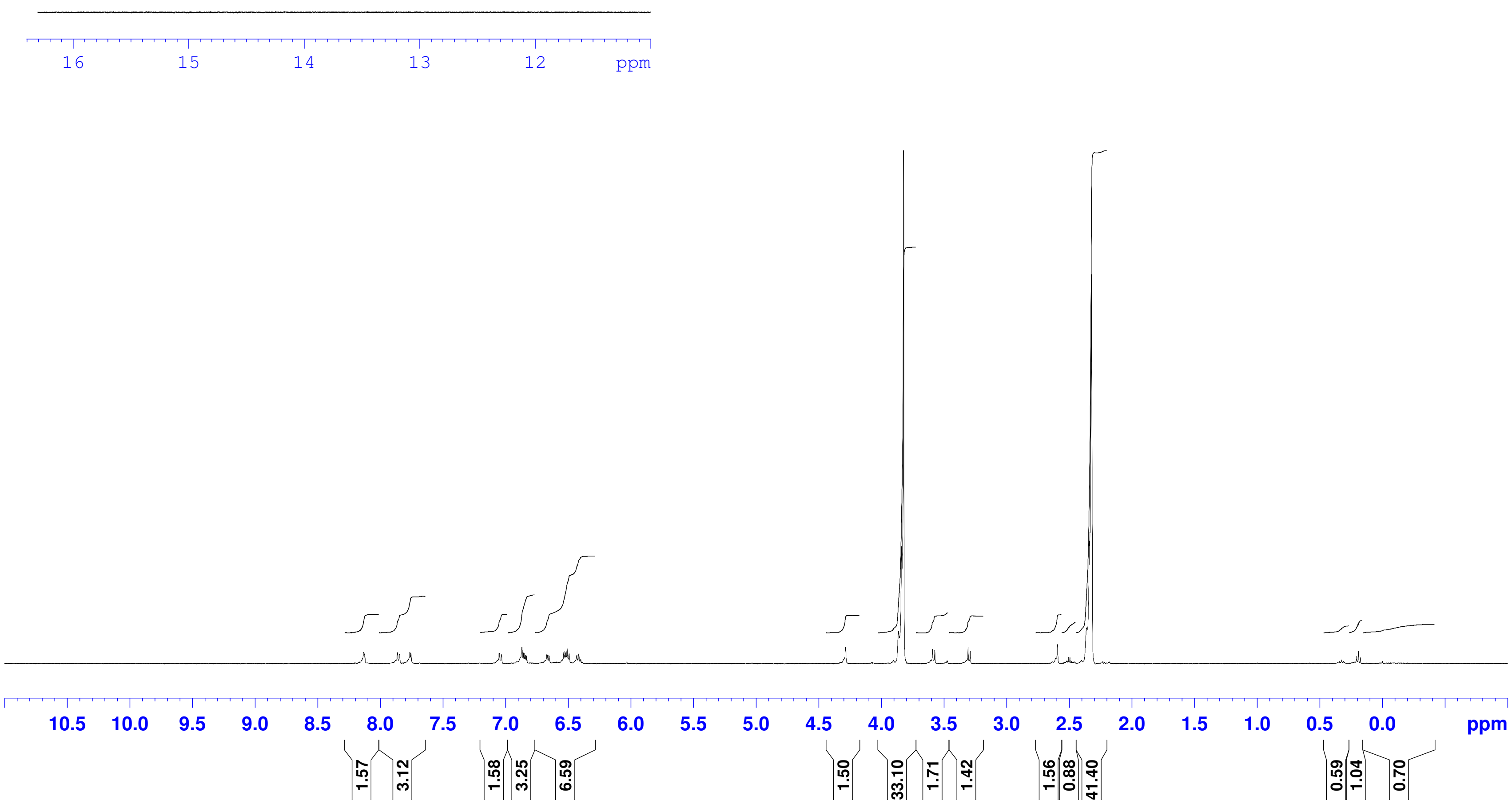

CARDIFF UNIVERSITY
PRIFYSGOL CAERDYDD

NAME	CM-SK87P
EXPNO	7
PROCNO	1
Date_	20141031
Time	13.26
INSTRUM	Avance500
PROBHD	5 mm QNP 1H/13
PULPROG	zg30
TD	65536
SOLVENT	DMSO
NS	16
DS	2
SWH	10330.578 Hz
FIDRES	0.157632 Hz
AQ	3.1719923 sec
RG	645
DW	48.400 use
DE	6.50 use
TE	298.1 K
D1	1.00000000 sec
TD0	1

======== CHANNEL f1 =======

NUC1	1H
P1	11.50 use
PL1	-1.00 dB
PL1W	11.38419914 W
SFO1	500.1330885 MHz
SI	32768
SF	500.1300989 MHz
WDW	EM
SSB	0
LB	0.30 Hz
GB	0
PC	1.00

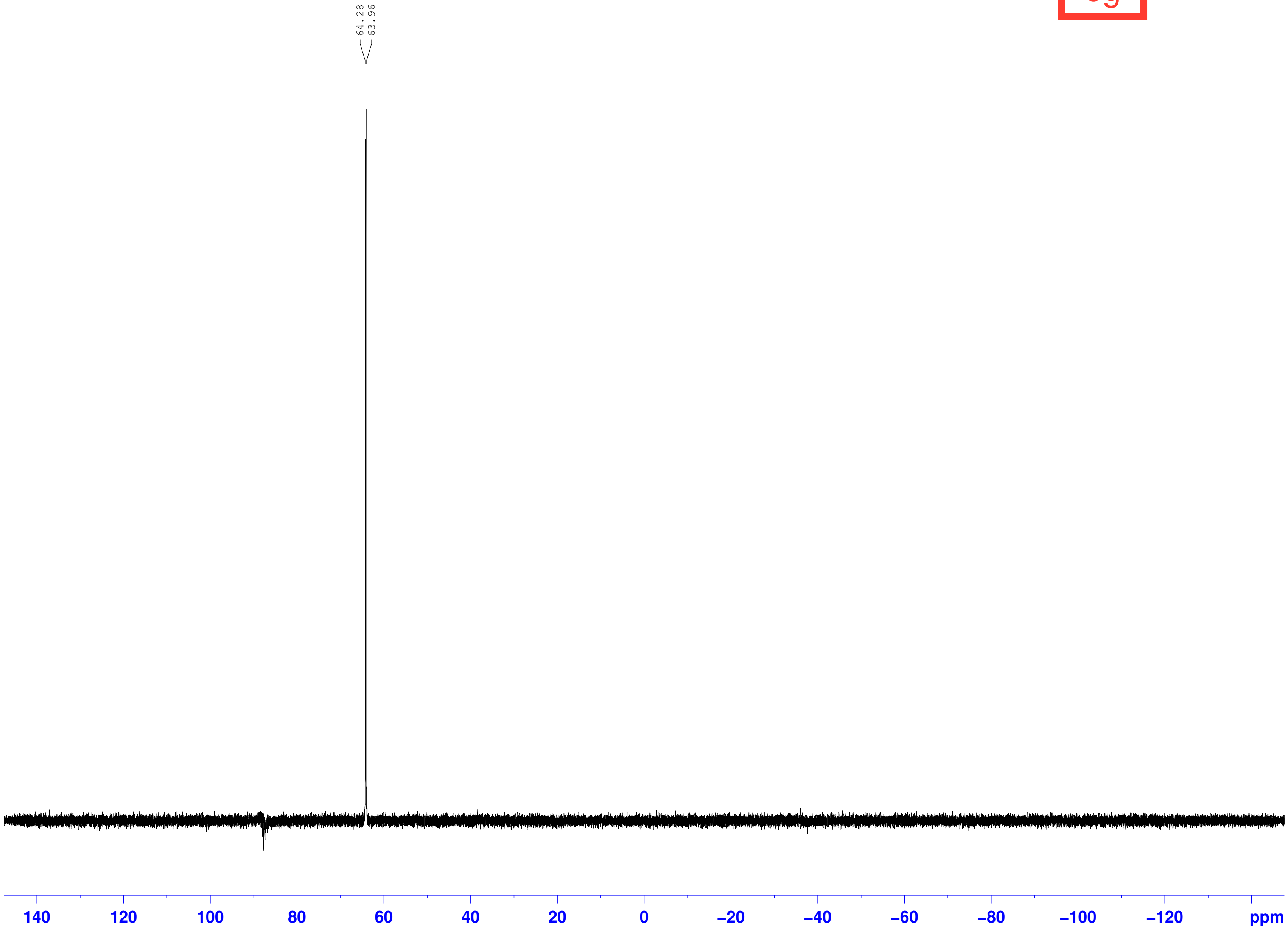

8g

CARDIFF UNIVERSITY
PRIFYSGOL CAERDYDD

NAME	CM-SK87P
EXPNO	3
PROCNO	1
Date_	20141006
Time	15.48
INSTRUM	Avance500
PROBHD	5 mm QNP 1H/13
PULPROG	zgfhigqn
TD	131072
SOLVENT	DMSO
NS	16
DS	4
SWH	138888.891 Hz
FIDRES	1.059638 Hz
AQ	0.4719092 sec
RG	2300
DW	3.600 use
DE	6.00 use
TE	290.0 K
D1	1.00000000 sec
D11	0.03000000 sec
D12	0.00002000 sec
TD0	1

======== CHANNEL f1 =======

NUC1	19F
P1	18.60 use
PL1	-1.50 dB
PL1W	11.14113998 W
SFO1	470.5923770 MHz

======== CHANNEL f2 =======

CPDPRG2	waltz16
NUC2	1H
PCPD2	80.00 use
PL2	-2.00 dB
PL12	14.85 dB
PL2W	14.33185768 W
PL12W	0.29600734 W
SFO2	500.1320005 MHz
SI	65536
SF	470.5923770 MHz
WDW	EM
SSB	0
LB	0.30 Hz
GB	0
PC	1.40

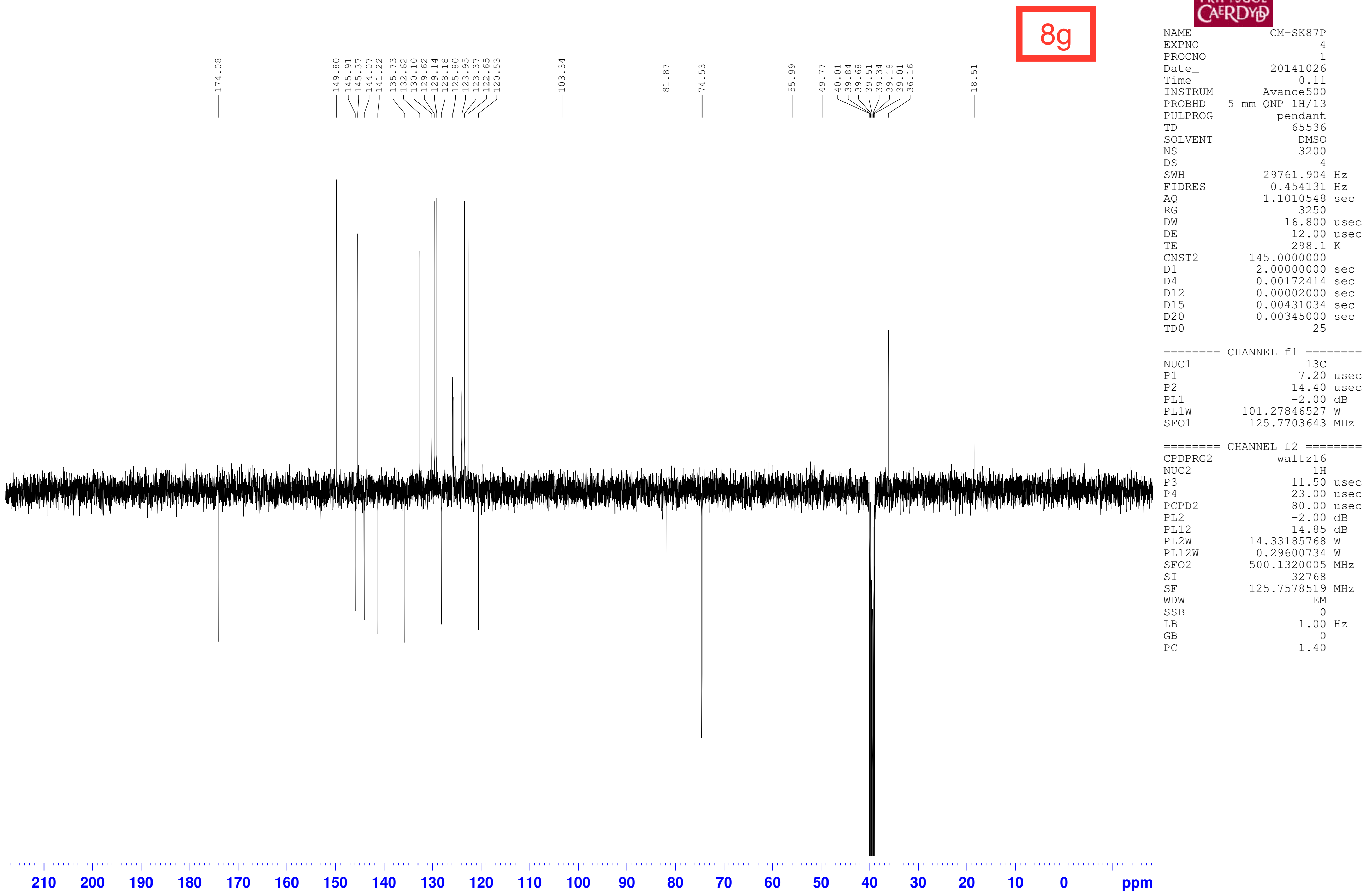
8g
174.08
149.80
145.91
145.37
144.07
141.22
135.73
132.62
130.10
129.62
129.14
128.18
125.80
123.95
123.37
122.65
120.53
103.34
81.87
74.53
55.99
49.77
40.01
39.84
39.68
39.51
39.34
39.18
39.01
36.16
18.51
210 200 190 180 170 160 150 140 130 120 110 100 90 80 70 60 50 40 30 20 10 0 ppm
NAME CM-SK87P
EXPNO 4
PROCNO 1
Date_ 20141026
Time 0.11
INSTRUM Avance500
PROBHD 5 mm QNP 1H/13
PULPROG pendant
TD 65536
SOLVENT DMSO
NS 3200
DS 4
SWH 29761.904 Hz
FIDRES 0.454131 Hz
AQ 1.1010548 sec
RG 3250
DW 16.800 usec
DE 12.00 usec
TE 298.1 K
CNST2 145.0000000
D1 2.00000000 sec
D4 0.00172414 sec
D12 0.00002000 sec
D15 0.00431034 sec
D20 0.00345000 sec
TD0 25
======== CHANNEL f1 ========
NUC1 13C
P1 7.20 usec
P2 14.40 usec
PL1 -2.00 dB
PL1W 101.27846527 W
SFO1 125.7703643 MHz
======== CHANNEL f2 ========
CPDPRG2 waltz16
NUC2 1H
P3 11.50 usec
P4 23.00 usec
PCPD2 80.00 usec
PL2 -2.00 dB
PL12 14.85 dB
PL2W 14.33185768 W
PL12W 0.29600734 W
SFO2 500.1320005 MHz
SI 32768
SF 125.7578519 MHz
WDW EM
SSB 0
LB 1.00 Hz
GB 0
PC 1.40

8g

MEDAC LTD

Analytical and chemical consultancy services

MEDAC Ltd
Alpha 319
Chobham Business Centre
Chertsey Road
Chobham
Surrey
GU24 8JB
United Kingdom

www.medacltd.com
Tel/Fax No. 01276 855410
Email: info@medacltd.com

A N A L Y T I C A L   R E P O R T

Date 29th July 2015

Name Dr Andrew Westwell

Sample ID SK87P

Formula $C_{23}H_{16}F_5N_3O_3S$

ELEMENT	C	H	N						
% Theory	54.23	3.17	8.24						
% Found 1	54.39	3.24	8.47						
% Found 2	53.99	3.09	8.20						

Comments: Insufficient sample for further analysis.

Assay No: 154658

Analyst: Richard Morris

8h

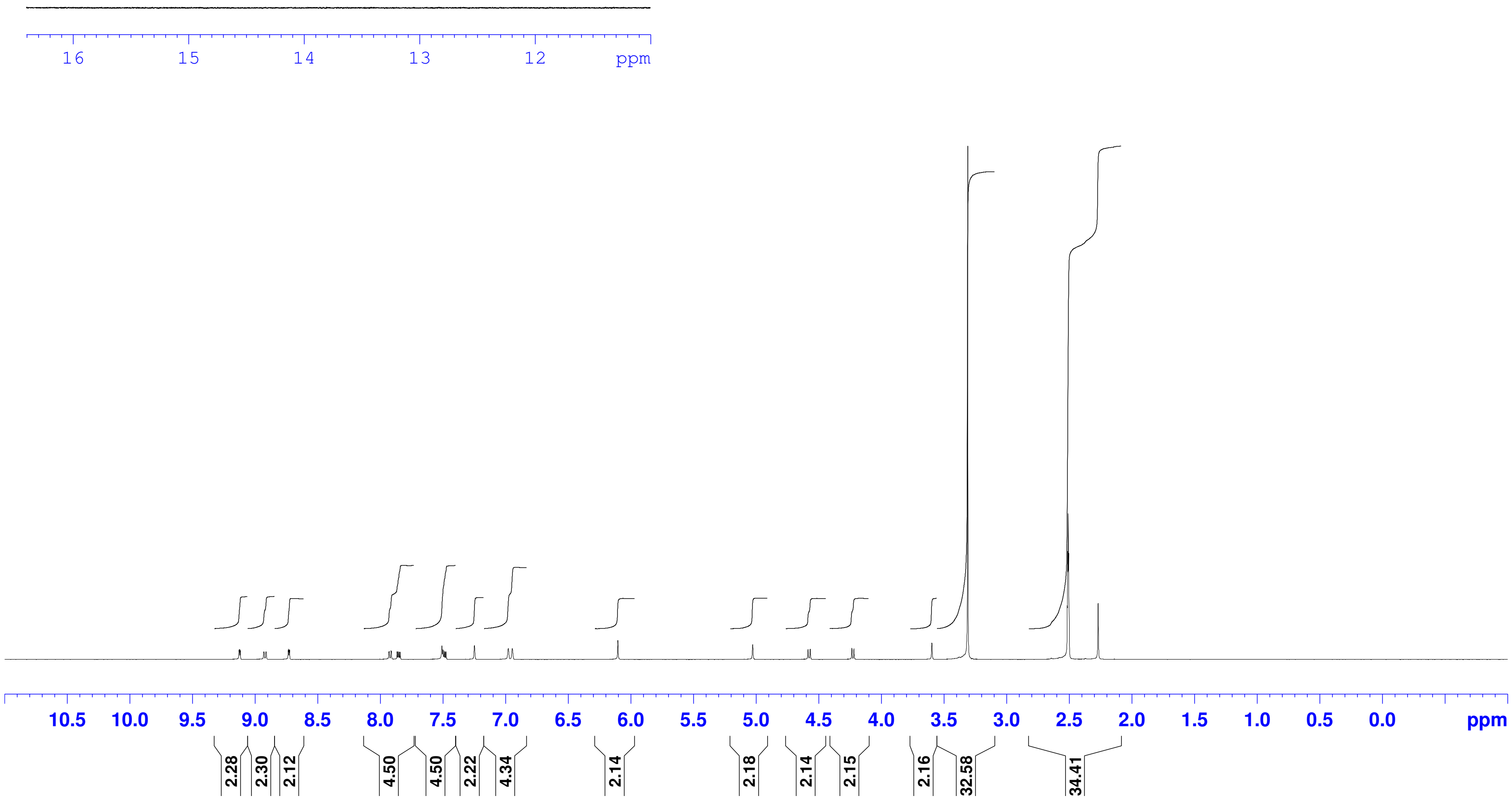

NAME	CM-SK88P
EXPNO	7
PROCNO	1
Date_	20141015
Time	12.57
INSTRUM	Avance500
PROBHD	5 mm QNP 1H/13
PULPROG	zg30
TD	65536
SOLVENT	DMSO
NS	16
DS	2
SWH	10330.578 Hz
FIDRES	0.157632 Hz
AQ	3.1719923 sec
RG	645
DW	48.400 use
DE	6.50 use
TE	298.1 K
D1	1.00000000 sec
TD0	1

======== CHANNEL f1 =======

NUC1	1H
P1	11.50 use
PL1	-1.00 dB
PL1W	11.38419914 W
SFO1	500.1330885 MHz
SI	32768
SF	500.1300000 MHz
WDW	EM
SSB	0
LB	0.30 Hz
GB	0
PC	1.00

8h

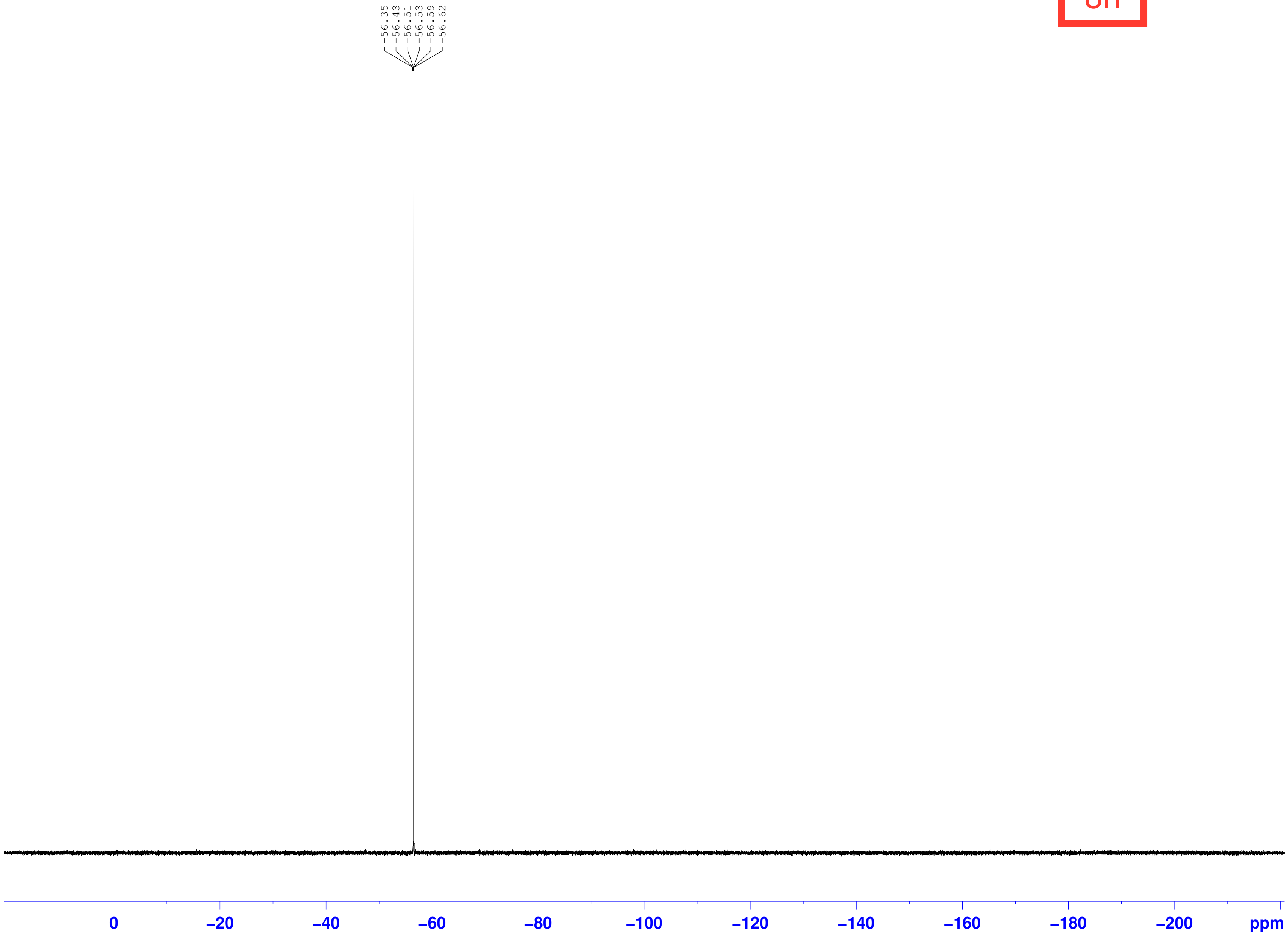

NAME	CM-SK88P
EXPNO	6
PROCNO	1
Date_	20141015
Time	11.46
INSTRUM	Avance500
PROBHD	5 mm QNP 1H/13
PULPROG	zgfhigqn
TD	131072
SOLVENT	DMSO
NS	16
DS	4
SWH	113636.367 Hz
FIDRES	0.866977 Hz
AQ	0.5767668 sec
RG	4100
DW	4.400 use
DE	6.00 use
TE	298.1 K
D1	1.00000000 sec
D11	0.03000000 sec
D12	0.00002000 sec
TD0	1

======== CHANNEL f1 =======

NUC1	19F
P1	18.60 use
PL1	-1.50 dB
PL1W	11.14113998 W
SFO1	470.5453180 MHz

======== CHANNEL f2 =======

CPDPRG2	waltz16
NUC2	1H
PCPD2	80.00 use
PL2	-2.00 dB
PL12	14.85 dB
PL2W	14.33185768 W
PL12W	0.29600734 W
SFO2	500.1320005 MHz
SI	65536
SF	470.5923770 MHz
WDW	EM
SSB	0
LB	0.30 Hz
GB	0
PC	1.40

8h

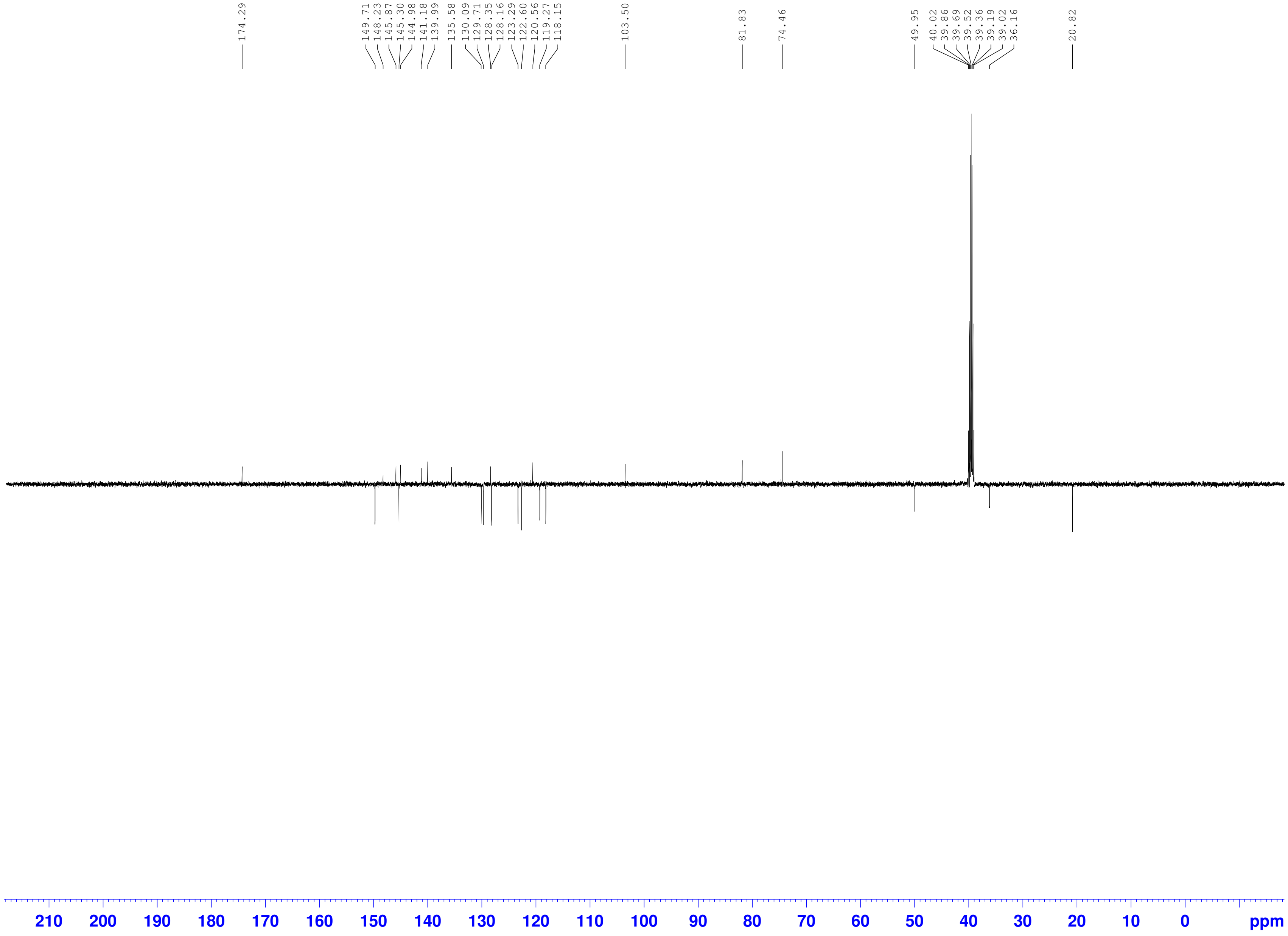

CARDIFF UNIVERSITY
PRIFYSGOL CAERDYDD

NAME	CM-SK88P
EXPNO	10
PROCNO	1
Date_	20141025
Time	20.46
INSTRUM	Avance500
PROBHD	5 mm QNP 1H/13
PULPROG	pendant
TD	65536
SOLVENT	DMSO
NS	3200
DS	4
SWH	29761.904 Hz
FIDRES	0.454131 Hz
AQ	1.1010548 sec
RG	3250
DW	16.800 usec
DE	12.00 usec
TE	298.1 K
CNST2	145.0000000
D1	2.00000000 sec
D4	0.00172414 sec
D12	0.00002000 sec
D15	0.00431034 sec
D20	0.00345000 sec
TD0	25

======== CHANNEL f1 ========

NUC1	13C
P1	7.20 usec
P2	14.40 usec
PL1	-2.00 dB
PL1W	101.27846527 W
SFO1	125.7703643 MHz

======== CHANNEL f2 ========

CPDPRG2	waltz16
NUC2	1H
P3	11.50 usec
P4	23.00 usec
PCPD2	80.00 usec
PL2	-2.00 dB
PL12	14.85 dB
PL2W	14.33185768 W
PL12W	0.29600734 W
SFO2	500.1320005 MHz
SI	32768
SF	125.7578519 MHz
WDW	EM
SSB	0
LB	1.00 Hz
GB	0
PC	1.40

8h

MEDAC LTD

Analytical and chemical consultancy services

MEDAC Ltd
Alpha 319
Chobham Business Centre
Chertsey Road
Chobham
Surrey
GU24 8JB
United Kingdom

www.medacltd.com
Tel/Fax No. 01276 855410
Email: info@medacltd.com

A N A L Y T I C A L   R E P O R T

Date 21st July 2015

Name Dr Andrew Westwell

Sample ID 5K88P

Formula $C_{25}H_{18}F_3N_3O_4$

**ELEMENT**	**C**	**H**	**N**						
**% Theory**	62.37	3.77	8.72						
**% Found 1**	62.32	3.75	8.70						
**% Found 2**	62.32	3.62	9.10						

Comments: All sample used.

Assay No: 154495

Analyst: Richard Morris

8i

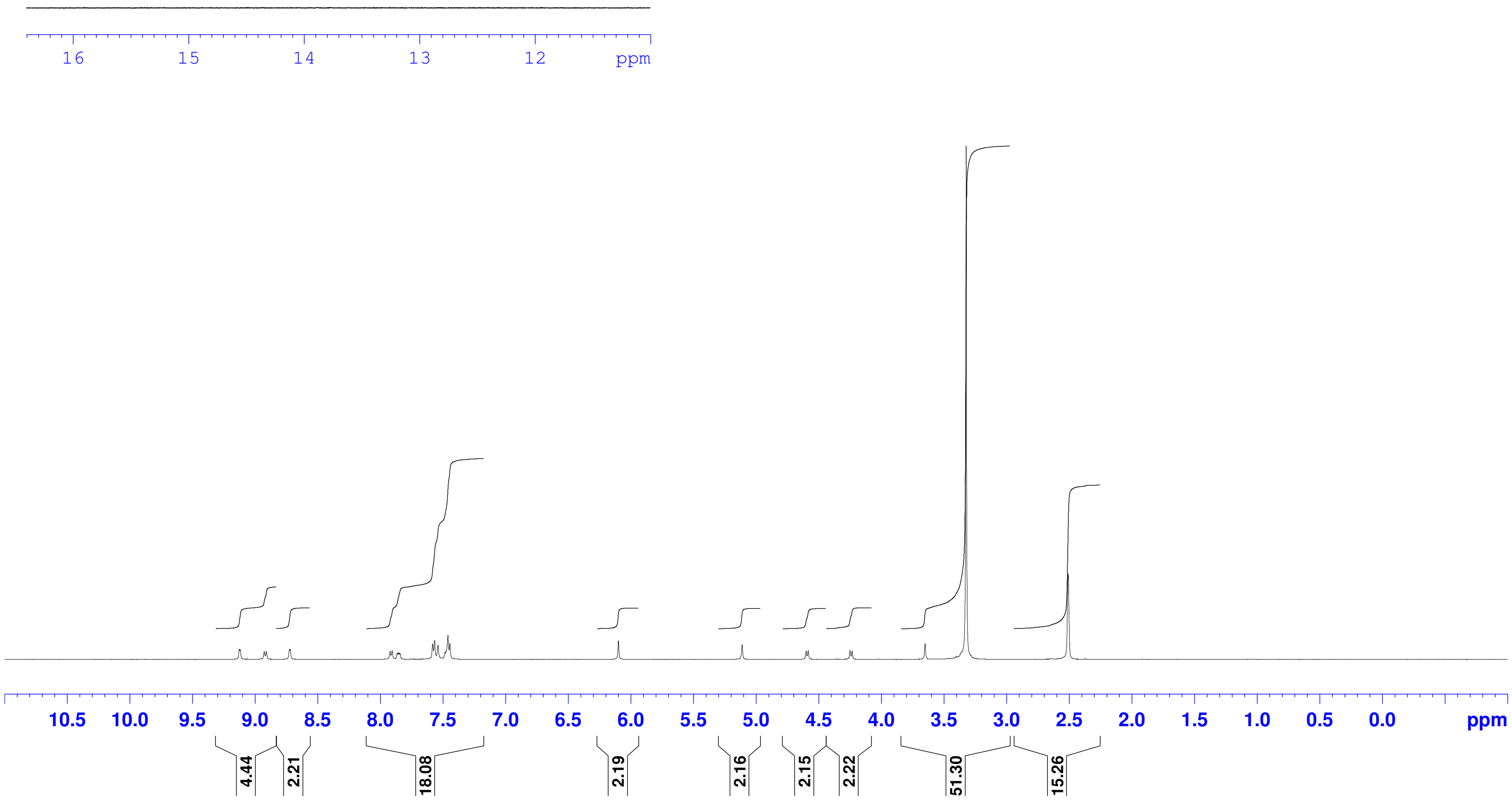

NAME	CM-SK89P
EXPNO	10
PROCNO	1
Date_	20141030
Time	16.28
INSTRUM	Avance500
PROBHD	5 mm QNP 1H/13
PULPROG	zg30
TD	65536
SOLVENT	DMSO
NS	16
DS	2
SWH	10330.578 Hz
FIDRES	0.157632 Hz
AQ	3.1719923 sec
RG	406
DW	48.400 use
DE	6.50 use
TE	298.1 K
D1	1.00000000 sec
TD0	1

======== CHANNEL f1 =======

NUC1	1H
P1	11.50 use
PL1	-1.00 dB
PL1W	11.38419914 W
SFO1	500.1330885 MHz
SI	32768
SF	500.1300000 MHz
WDW	EM
SSB	0
LB	0.30 Hz
GB	0
PC	1.00

8i

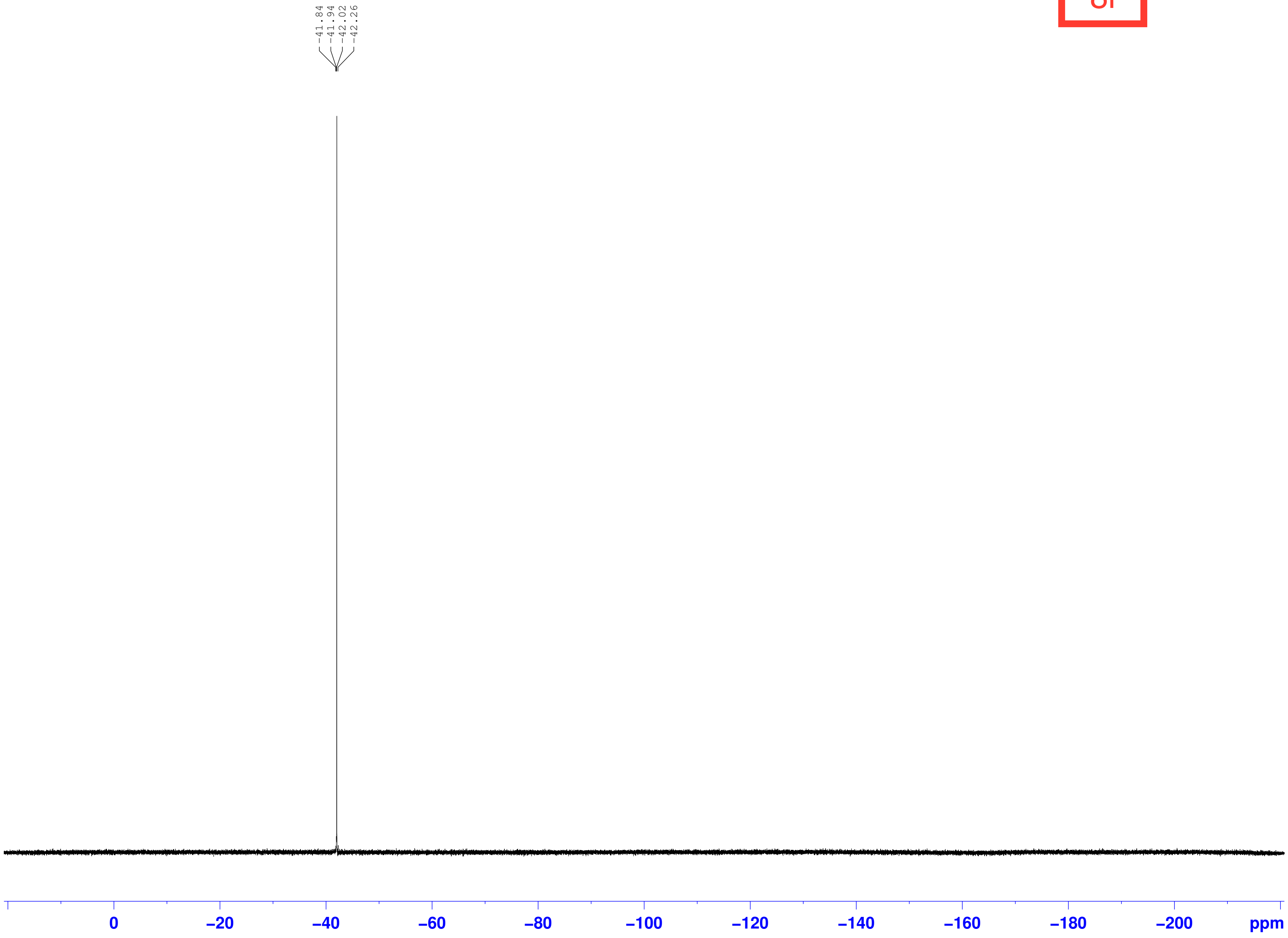

NAME	CM-SK89P	
EXPNO	9	
PROCNO	1	
Date_	20141030	
Time	16.26	
INSTRUM	Avance500	
PROBHD	5 mm QNP 1H/13	
PULPROG	zgfhigqn	
TD	131072	
SOLVENT	DMSO	
NS	16	
DS	4	
SWH	113636.367	Hz
FIDRES	0.866977	Hz
AQ	0.5767668	sec
RG	4100	
DW	4.400	use
DE	6.00	use
TE	298.1	K
D1	1.00000000	sec
D11	0.03000000	sec
D12	0.00002000	sec
TD0	1	

======== CHANNEL f1 =======

NUC1	19F	
P1	18.60	use
PL1	-1.50	dB
PL1W	11.14113998	W
SFO1	470.5453180	MHz

======== CHANNEL f2 =======

CPDPRG2	waltz16	
NUC2	1H	
PCPD2	80.00	use
PL2	-2.00	dB
PL12	14.85	dB
PL2W	14.33185768	W
PL12W	0.29600734	W
SFO2	500.1320005	MHz
SI	65536	
SF	470.5923770	MHz
WDW	EM	
SSB	0	
LB	0.30	Hz
GB	0	
PC	1.40	

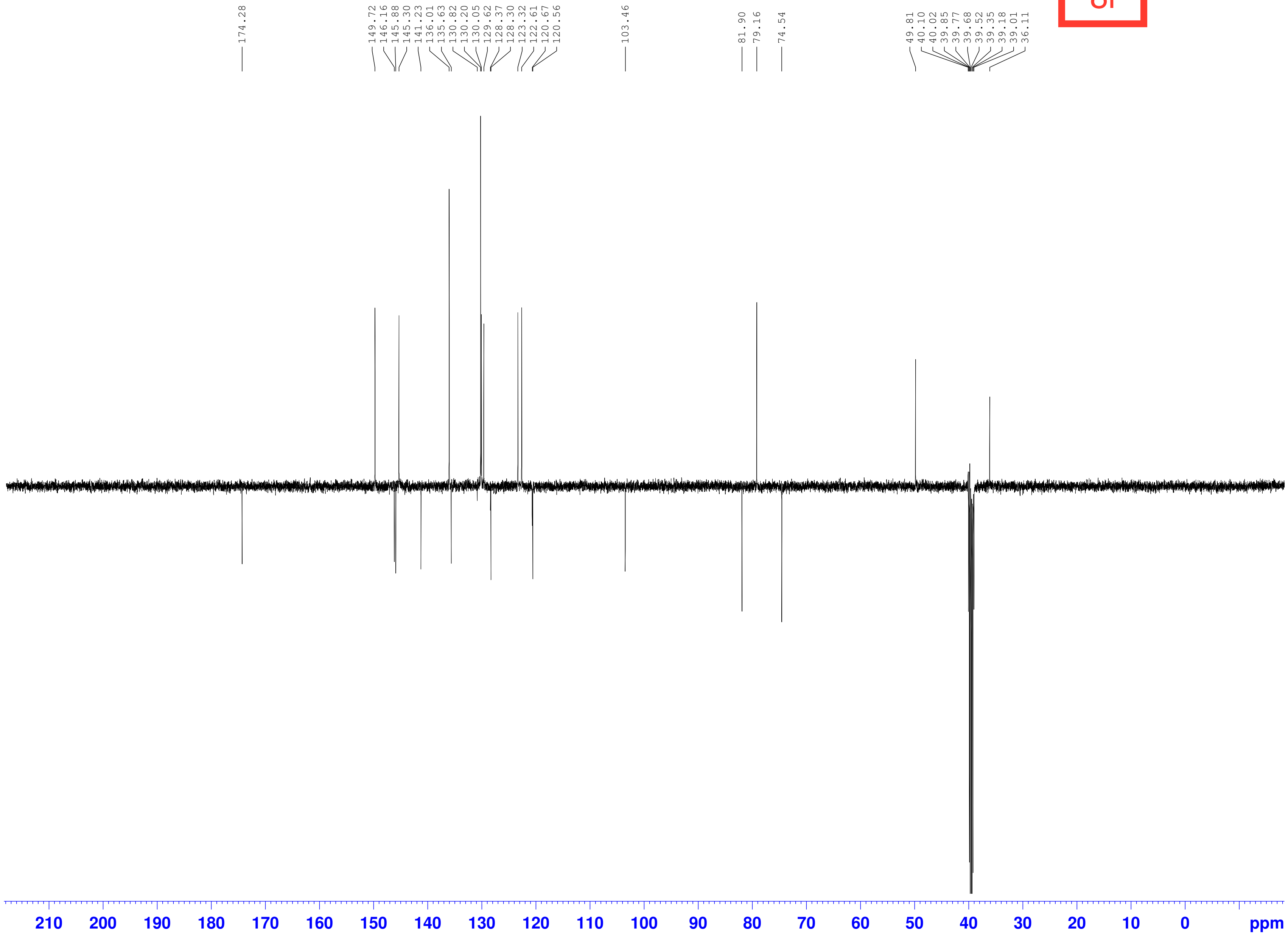

8i

CARDIFF UNIVERSITY PRIFYSGOL CAERDYDD

NAME	CM-SK89P	
EXPNO	12	
PROCNO	1	
Date_	20141103	
Time	23.49	
INSTRUM	Avance500	
PROBHD	5 mm QNP 1H/13	
PULPROG	pendant	
TD	65536	
SOLVENT	DMSO	
NS	2560	
DS	4	
SWH	29761.904	Hz
FIDRES	0.454131	Hz
AQ	1.1010548	sec
RG	3250	
DW	16.800	usec
DE	12.00	usec
TE	298.1	K
CNST2	145.0000000	
D1	2.00000000	sec
D4	0.00172414	sec
D12	0.00002000	sec
D15	0.00431034	sec
D20	0.00345000	sec
TD0	20	

======== CHANNEL f1 ========

NUC1	13C	
P1	7.20	usec
P2	14.40	usec
PL1	-2.00	dB
PL1W	101.27846527	W
SFO1	125.7703643	MHz

======== CHANNEL f2 ========

CPDPRG2	waltz16	
NUC2	1H	
P3	11.50	usec
P4	23.00	usec
PCPD2	80.00	usec
PL2	-2.00	dB
PL12	14.85	dB
PL2W	14.33185768	W
PL12W	0.29600734	W
SFO2	500.1320005	MHz
SI	32768	
SF	125.7578519	MHz
WDW	EM	
SSB	0	
LB	1.00	Hz
GB	0	
PC	1.40	

8i

MEDAC LTD

Analytical and chemical consultancy services

MEDAC Ltd
Alpha 319
Chobham Business Centre
Chertsey Road
Chobham
Surrey
GU24 8JB
United Kingdom

www.medacltd.com
Tel/Fax No. 01276 855410
Email: info@medacltd.com

A N A L Y T I C A L   R E P O R T

Date 21st July 2015

Name Dr Andrew Westwell

Sample ID 5K89P

Formula $C_{24}H_{18}F_3N_3O_3S$

ELEMENT	C	H	N						
% Theory	59.62	3.34	8.69						
% Found 1	59.29	3.35	8.55						
% Found 2	59.19	3.15	8.33						

Comments: All sample used.

Assay No: 154499

Analyst: Richard Morris

8j

NAME	CM-SK90P
EXPNO	5
PROCNO	1
Date_	20141004
Time	1.49
INSTRUM	Avance500
PROBHD	5 mm QNP 1H/13
PULPROG	zg30
TD	65536
SOLVENT	DMSO
NS	32
DS	2
SWH	10330.578 Hz
FIDRES	0.157632 Hz
AQ	3.1719923 sec
RG	645
DW	48.400 use
DE	6.50 use
TE	289.9 K
D1	1.00000000 sec
TD0	1

======== CHANNEL f1 =======

NUC1	1H
P1	11.50 use
PL1	-1.00 dB
PL1W	11.38419914 W
SFO1	500.1330885 MHz
SI	32768
SF	500.1300000 MHz
WDW	EM
SSB	0
LB	0.30 Hz
GB	0
PC	1.00

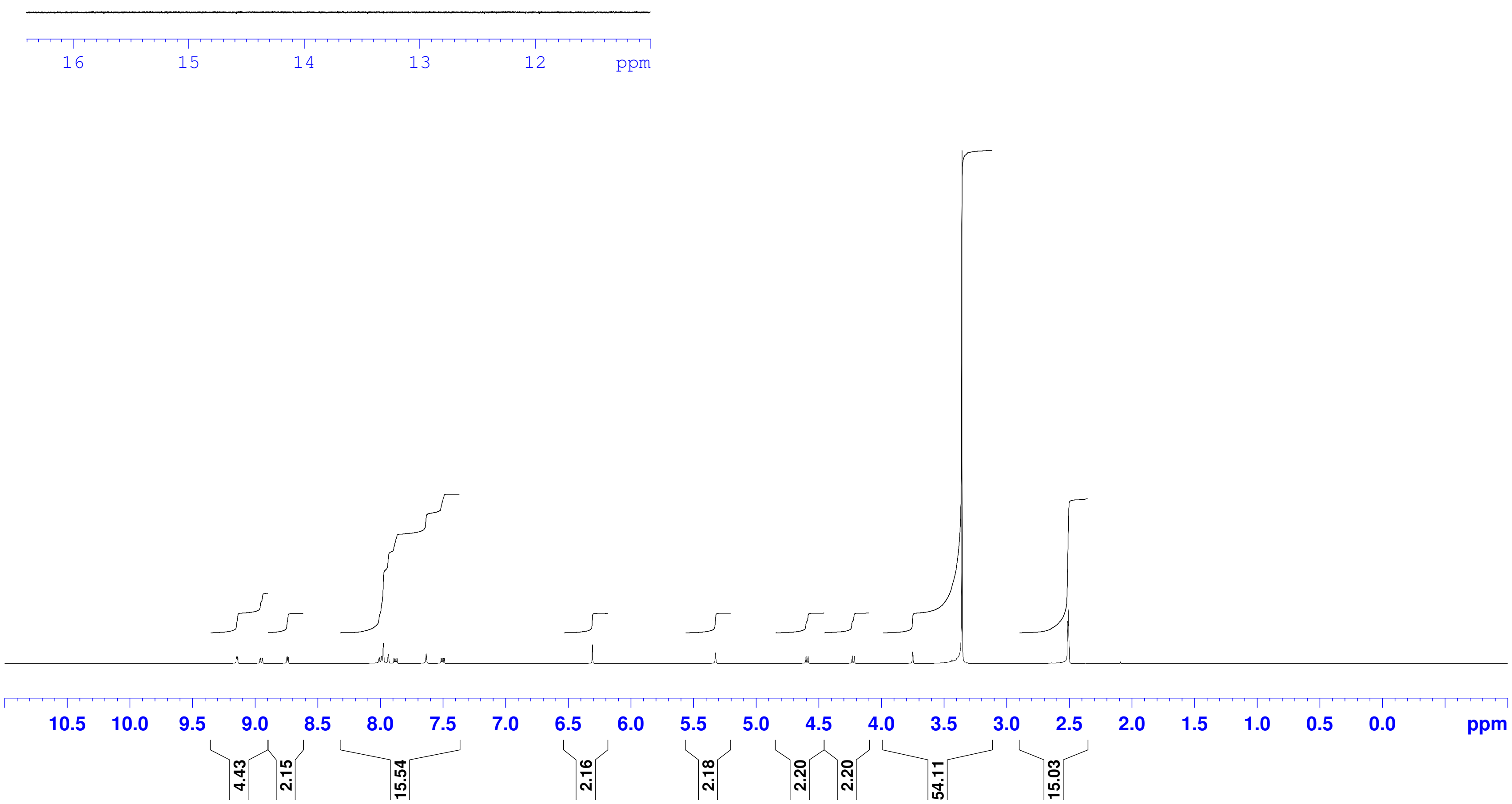

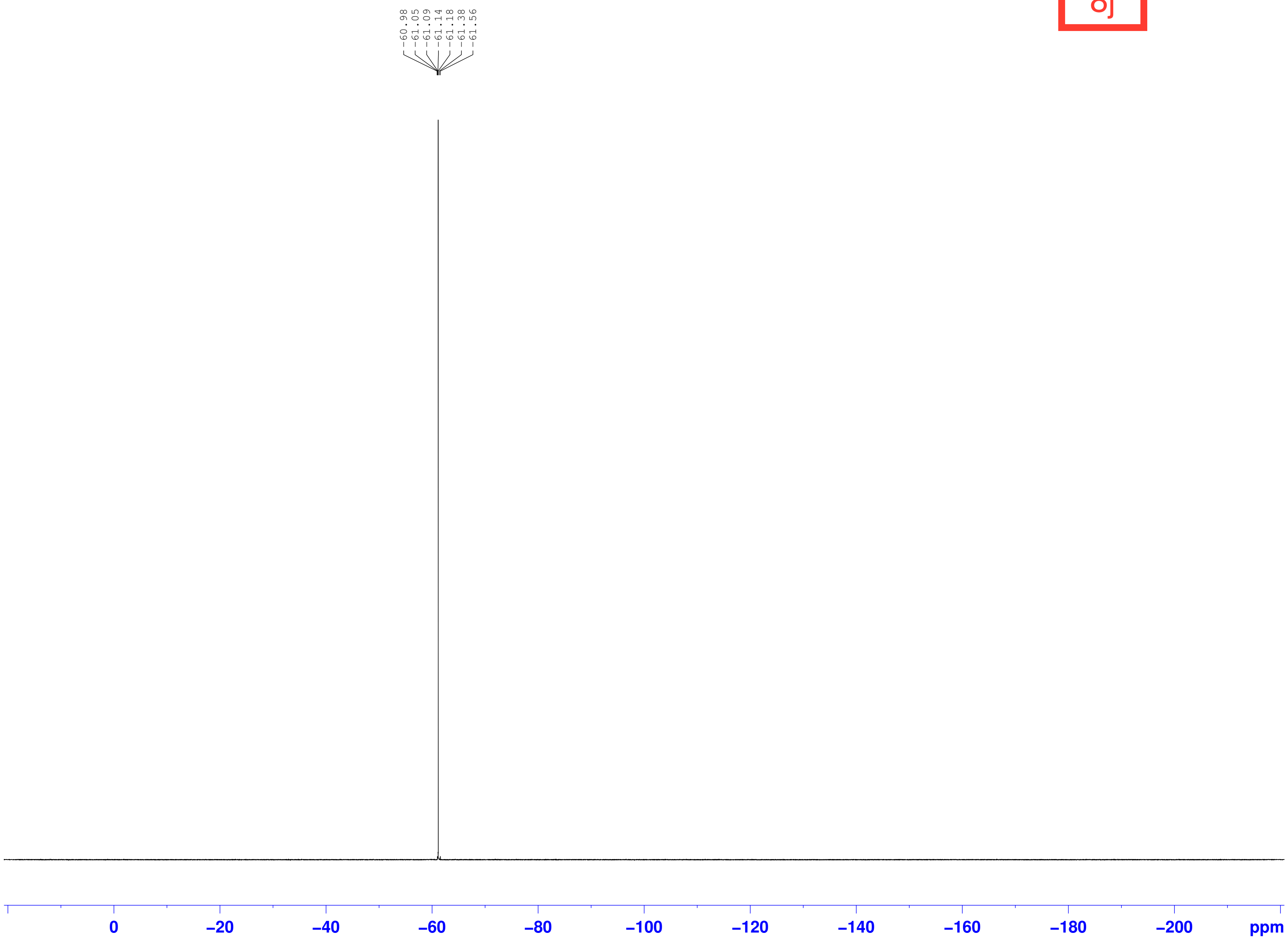

8j

CARDIFF UNIVERSITY
PRIFYSGOL CAERDYDD

NAME	CM-SK90P
EXPNO	3
PROCNO	1
Date_	20141003
Time	15.07
INSTRUM	Avance500
PROBHD	5 mm QNP 1H/13
PULPROG	zgfhigqn
TD	131072
SOLVENT	DMSO
NS	16
DS	4
SWH	113636.367 Hz
FIDRES	0.866977 Hz
AQ	0.5767668 sec
RG	2580
DW	4.400 use
DE	6.00 use
TE	289.5 K
D1	1.00000000 sec
D11	0.03000000 sec
D12	0.00002000 sec
TD0	1

======== CHANNEL f1 =======

NUC1	19F
P1	18.60 use
PL1	-1.50 dB
PL1W	11.14113998 W
SFO1	470.5453180 MHz

======== CHANNEL f2 =======

CPDPRG2	waltz16
NUC2	1H
PCPD2	80.00 use
PL2	-2.00 dB
PL12	14.85 dB
PL2W	14.33185768 W
PL12W	0.29600734 W
SFO2	500.1320005 MHz
SI	65536
SF	470.5923770 MHz
WDW	EM
SSB	0
LB	0.30 Hz
GB	0
PC	1.40

CARDIFF UNIVERSITY
PRIFYSGOL CAERDYDD

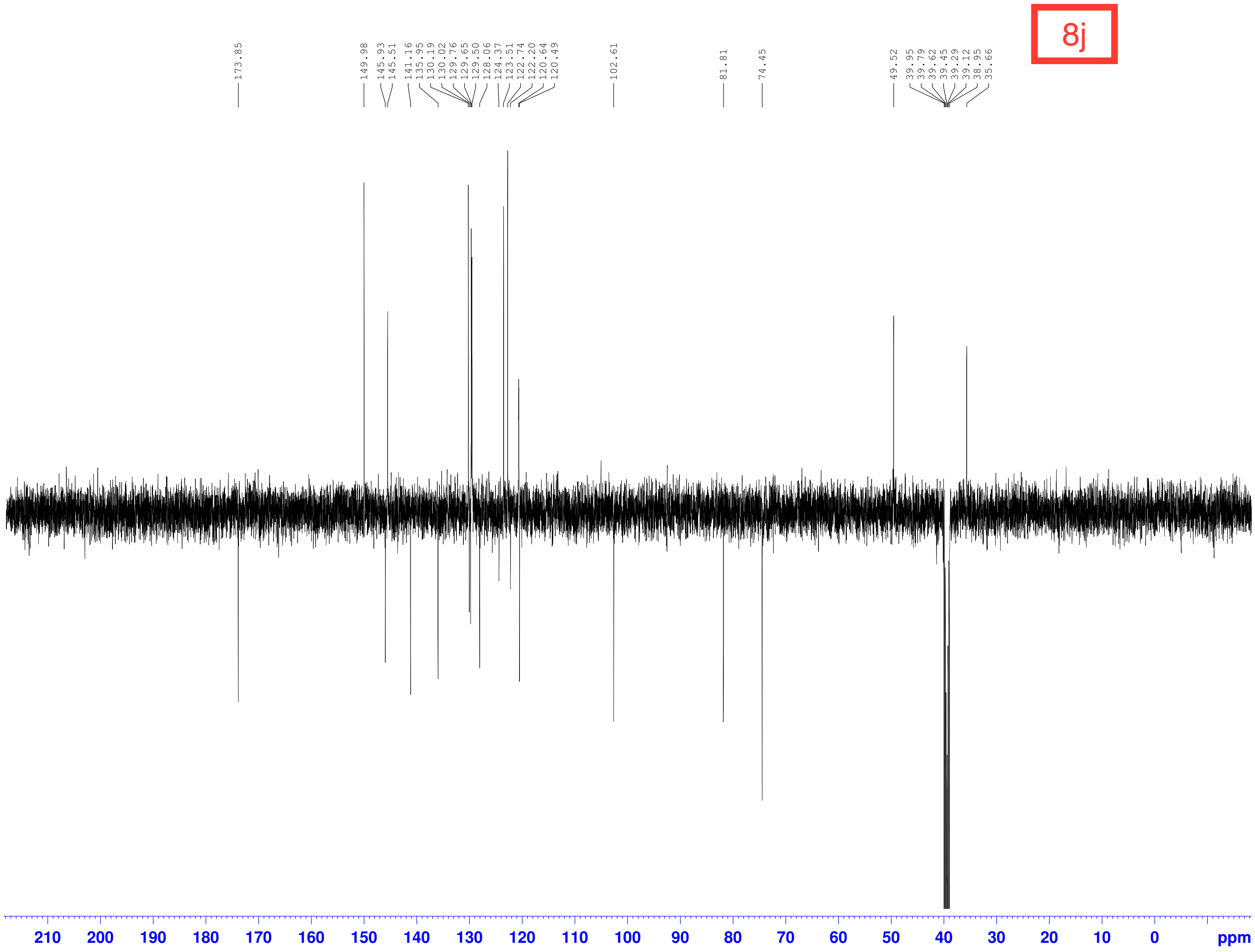

NAME	CM-SK90P
EXPNO	4
PROCNO	1
Date_	20141004
Time	0.39
INSTRUM	Avance500
PROBHD	5 mm QNP 1H/13
PULPROG	pendant
TD	65536
SOLVENT	DMSO
NS	1408
DS	4
SWH	29761.904 Hz
FIDRES	0.454131 Hz
AQ	1.1010548 sec
RG	3250
DW	16.800 usec
DE	12.00 usec
TE	290.5 K
CNST2	145.0000000
D1	2.00000000 sec
D4	0.00172414 sec
D12	0.00002000 sec
D15	0.00431034 sec
D20	0.00345000 sec
TD0	11

======== CHANNEL f1 ========

NUC1	13C
P1	7.20 usec
P2	14.40 usec
PL1	-2.00 dB
PL1W	101.27846527 W
SFO1	125.7703643 MHz

======== CHANNEL f2 ========

CPDPRG2	waltz16
NUC2	1H
P3	11.50 usec
P4	23.00 usec
PCPD2	80.00 usec
PL2	-2.00 dB
PL12	14.85 dB
PL2W	14.33185768 W
PL12W	0.29600734 W
SFO2	500.1320005 MHz
SI	32768
SF	125.7578519 MHz
WDW	EM
SSB	0
LB	1.00 Hz
GB	0
PC	1.40

8j

MEDAC LTD

Analytical and chemical consultancy services

MEDAC Ltd
Alpha 319
Chobham Business Centre
Chertsey Road
Chobham
Surrey
GU24 8JB
United Kingdom

www.medacltd.com
Tel/Fax No. 01276 855410
Email: info@medacltd.com

A N A L Y T I C A L   R E P O R T

Date 21st July 2015

Name Dr Andrew Westwell

Sample ID 5K90P

Formula $C_{25}H_{15}F_6N_3O_3$

ELEMENT	C	H	N						
% Theory	57.81	2.91	8.09						
% Found 1	57.60	2.96	8.21						
% Found 2	57.51	2.99	8.13						

Comments: All sample used.

Assay No: 154498

Analyst: Richard Morris

8k

NAME	CM-SK91P
EXPNO	14
PROCNO	1
Date_	20141027
Time	11.47
INSTRUM	Avance500
PROBHD	5 mm QNP 1H/13
PULPROG	zg30
TD	65536
SOLVENT	DMSO
NS	16
DS	2
SWH	10330.578 Hz
FIDRES	0.157632 Hz
AQ	3.1719923 sec
RG	645
DW	48.400 use
DE	6.50 use
TE	298.1 K
D1	1.00000000 sec
TD0	1

======== CHANNEL f1 =======

NUC1	1H
P1	11.50 use
PL1	-1.00 dB
PL1W	11.38419914 W
SFO1	500.1330885 MHz
SI	32768
SF	500.1300000 MHz
WDW	EM
SSB	0
LB	0.30 Hz
GB	0
PC	1.00

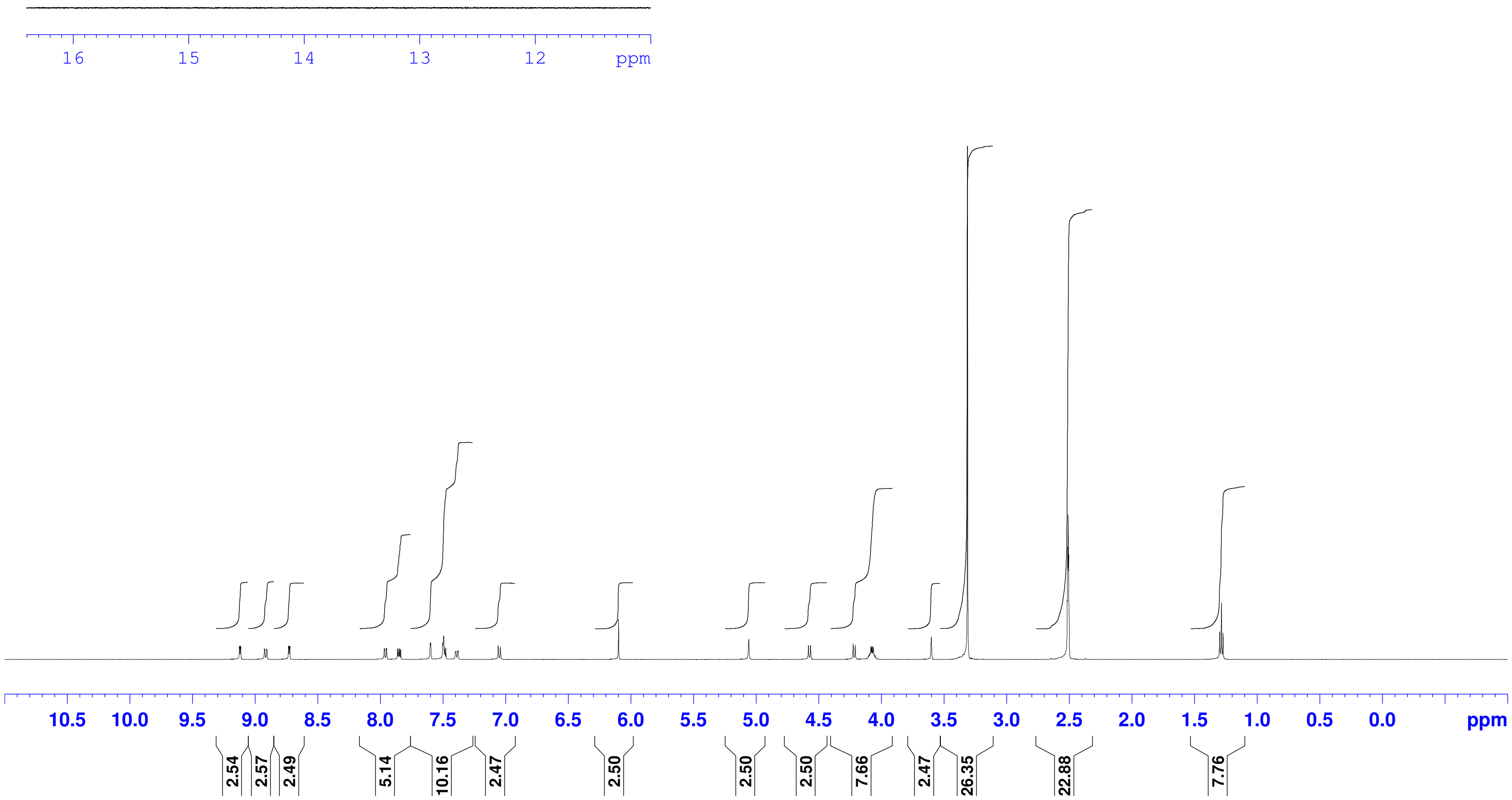

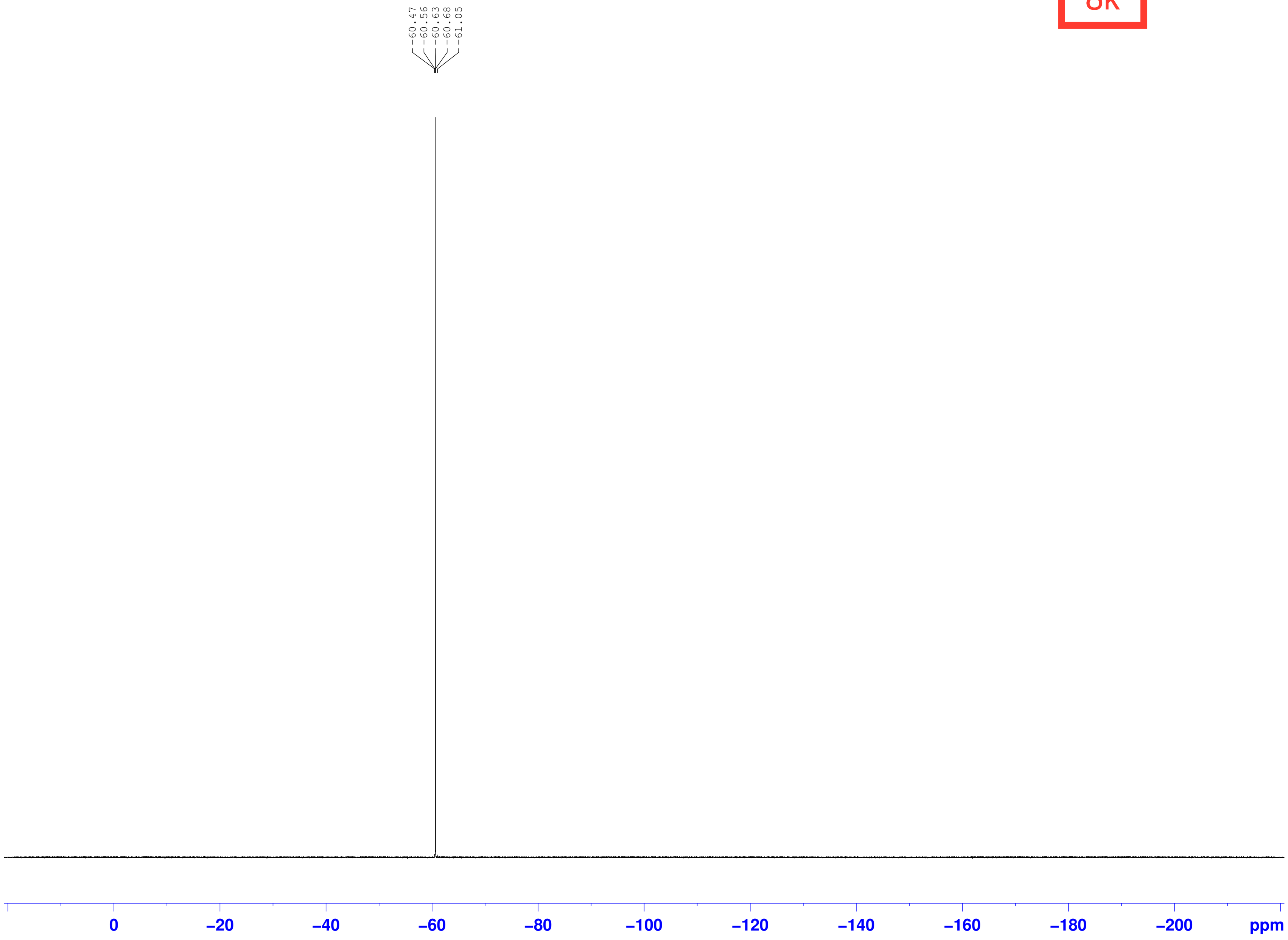

8k

CARDIFF UNIVERSITY
PRIFYSGOL CAERDYDD

NAME	CM-SK91P
EXPNO	15
PROCNO	1
Date_	20141027
Time	11.49
INSTRUM	Avance500
PROBHD	5 mm QNP 1H/13
PULPROG	zgfhigqn
TD	131072
SOLVENT	DMSO
NS	16
DS	4
SWH	113636.367 Hz
FIDRES	0.866977 Hz
AQ	0.5767668 sec
RG	4100
DW	4.400 use
DE	6.00 use
TE	298.2 K
D1	1.00000000 sec
D11	0.03000000 sec
D12	0.00002000 sec
TD0	1
======== CHANNEL f1 =======	
NUC1	19F
P1	18.60 use
PL1	-1.50 dB
PL1W	11.14113998 W
SFO1	470.5453180 MHz
======== CHANNEL f2 =======	
CPDPRG2	waltz16
NUC2	1H
PCPD2	80.00 use
PL2	-2.00 dB
PL12	14.85 dB
PL2W	14.33185768 W
PL12W	0.29600734 W
SFO2	500.1320005 MHz
SI	65536
SF	470.5923770 MHz
WDW	EM
SSB	0
LB	0.30 Hz
GB	0
PC	1.40

8k

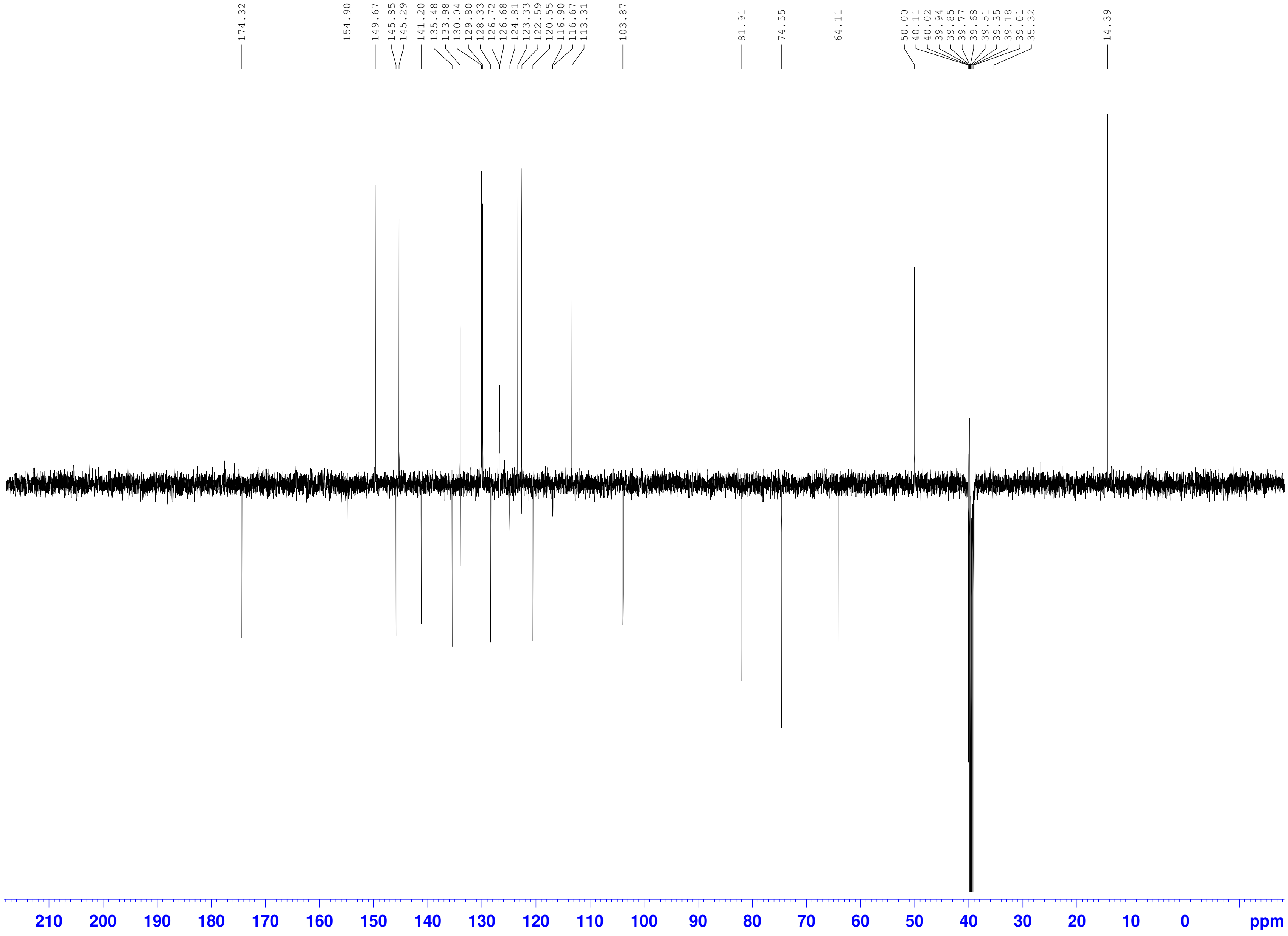

CARDIFF UNIVERSITY
PRIFYSGOL CAERDYDD

NAME	CM-SK91P
EXPNO	17
PROCNO	1
Date_	20141103
Time	21.19
INSTRUM	Avance500
PROBHD	5 mm QNP 1H/13
PULPROG	pendant
TD	65536
SOLVENT	DMSO
NS	2560
DS	4
SWH	29761.904 Hz
FIDRES	0.454131 Hz
AQ	1.1010548 sec
RG	3250
DW	16.800 usec
DE	12.00 usec
TE	298.1 K
CNST2	145.0000000
D1	2.00000000 sec
D4	0.00172414 sec
D12	0.00002000 sec
D15	0.00431034 sec
D20	0.00345000 sec
TD0	20

======== CHANNEL f1 ========

NUC1	13C
P1	7.20 usec
P2	14.40 usec
PL1	-2.00 dB
PL1W	101.27846527 W
SFO1	125.7703643 MHz

======== CHANNEL f2 ========

CPDPRG2	waltz16
NUC2	1H
P3	11.50 usec
P4	23.00 usec
PCPD2	80.00 usec
PL2	-2.00 dB
PL12	14.85 dB
PL2W	14.33185768 W
PL12W	0.29600734 W
SFO2	500.1320005 MHz
SI	32768
SF	125.7578519 MHz
WDW	EM
SSB	0
LB	1.00 Hz
GB	0
PC	1.40

8k

MEDAC LTD

Analytical and chemical consultancy services

MEDAC Ltd
Alpha 319
Chobham Business Centre
Chertsey Road
Chobham
Surrey
GU24 8JB
United Kingdom

www.medacltd.com
Tel/Fax No. 01276 855410
Email: info@medacltd.com

A N A L Y T I C A L   R E P O R T

Date 21st July 2015

Name Dr Andrew Westwell

Sample ID 5K91P

Formula $C_{26}H_{20}F_3N_3O_4$

ELEMENT	C	H	N						
% Theory	63.03	4.07	8.48						
% Found 1	62.68	3.98	8.55						
% Found 2	62.56	4.01	8.72						

Comments: All sample used.

Assay No: 154497

Analyst: Richard Morris

8l

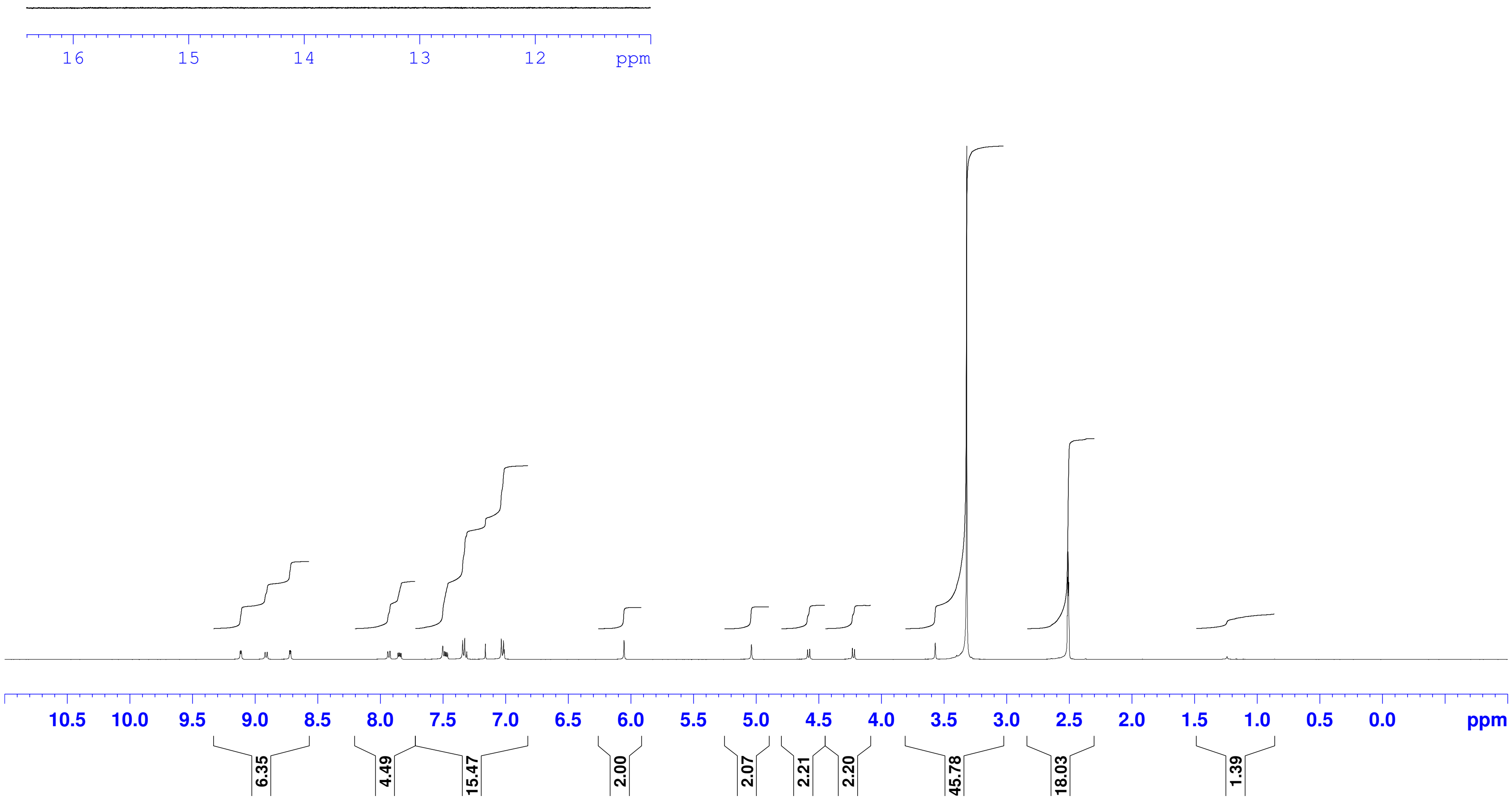

CARDIFF UNIVERSITY
PRIFYSGOL CAERDYDD

NAME	CM-SK92P	
EXPNO	5	
PROCNO	1	
Date_	20141020	
Time	10.43	
INSTRUM	Avance500	
PROBHD	5 mm QNP 1H/13	
PULPROG	zg30	
TD	65536	
SOLVENT	DMSO	
NS	16	
DS	2	
SWH	10330.578	Hz
FIDRES	0.157632	Hz
AQ	3.1719923	sec
RG	645	
DW	48.400	use
DE	6.50	use
TE	298.1	K
D1	1.00000000	sec
TD0	1	
======== CHANNEL f1 =======		
NUC1	1H	
P1	11.50	use
PL1	-1.00	dB
PL1W	11.38419914	W
SFO1	500.1330885	MHz
SI	32768	
SF	500.1300000	MHz
WDW	EM	
SSB	0	
LB	0.30	Hz
GB	0	
PC	1.00	

8l

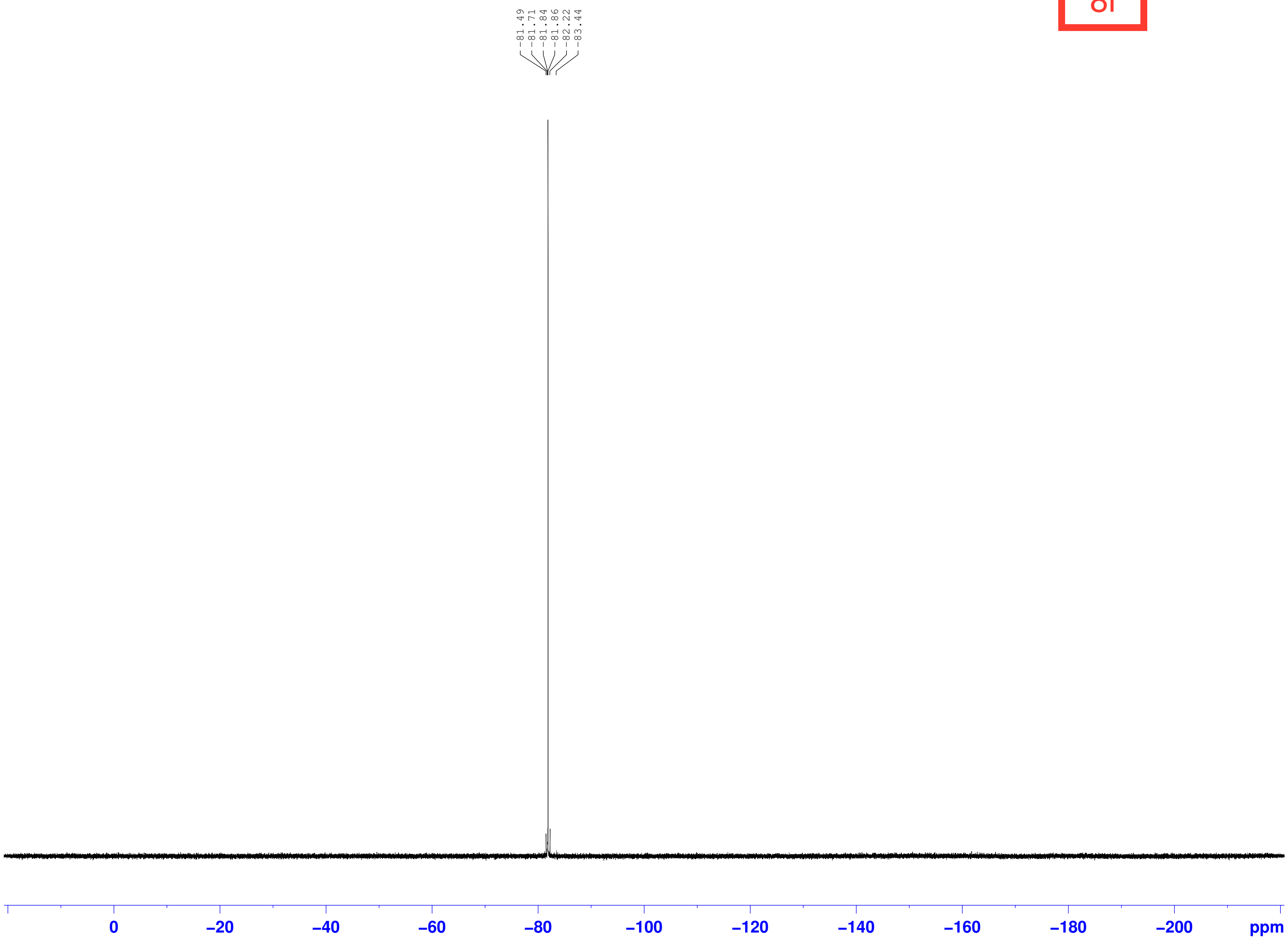

NAME	CM-SK92P
EXPNO	4
PROCNO	1
Date_	20141020
Time	10.40
INSTRUM	Avance500
PROBHD	5 mm QNP 1H/13
PULPROG	zgfhigqn
TD	131072
SOLVENT	DMSO
NS	16
DS	4
SWH	113636.367 Hz
FIDRES	0.866977 Hz
AQ	0.5767668 sec
RG	4100
DW	4.400 use
DE	6.00 use
TE	298.2 K
D1	1.00000000 sec
D11	0.03000000 sec
D12	0.00002000 sec
TD0	1

======== CHANNEL f1 =======

NUC1	19F
P1	18.60 use
PL1	-1.50 dB
PL1W	11.14113998 W
SFO1	470.5453180 MHz

======== CHANNEL f2 =======

CPDPRG2	waltz16
NUC2	1H
PCPD2	80.00 use
PL2	-2.00 dB
PL12	14.85 dB
PL2W	14.33185768 W
PL12W	0.29600734 W
SFO2	500.1320005 MHz
SI	65536
SF	470.5923770 MHz
WDW	EM
SSB	0
LB	0.30 Hz
GB	0
PC	1.40

8l

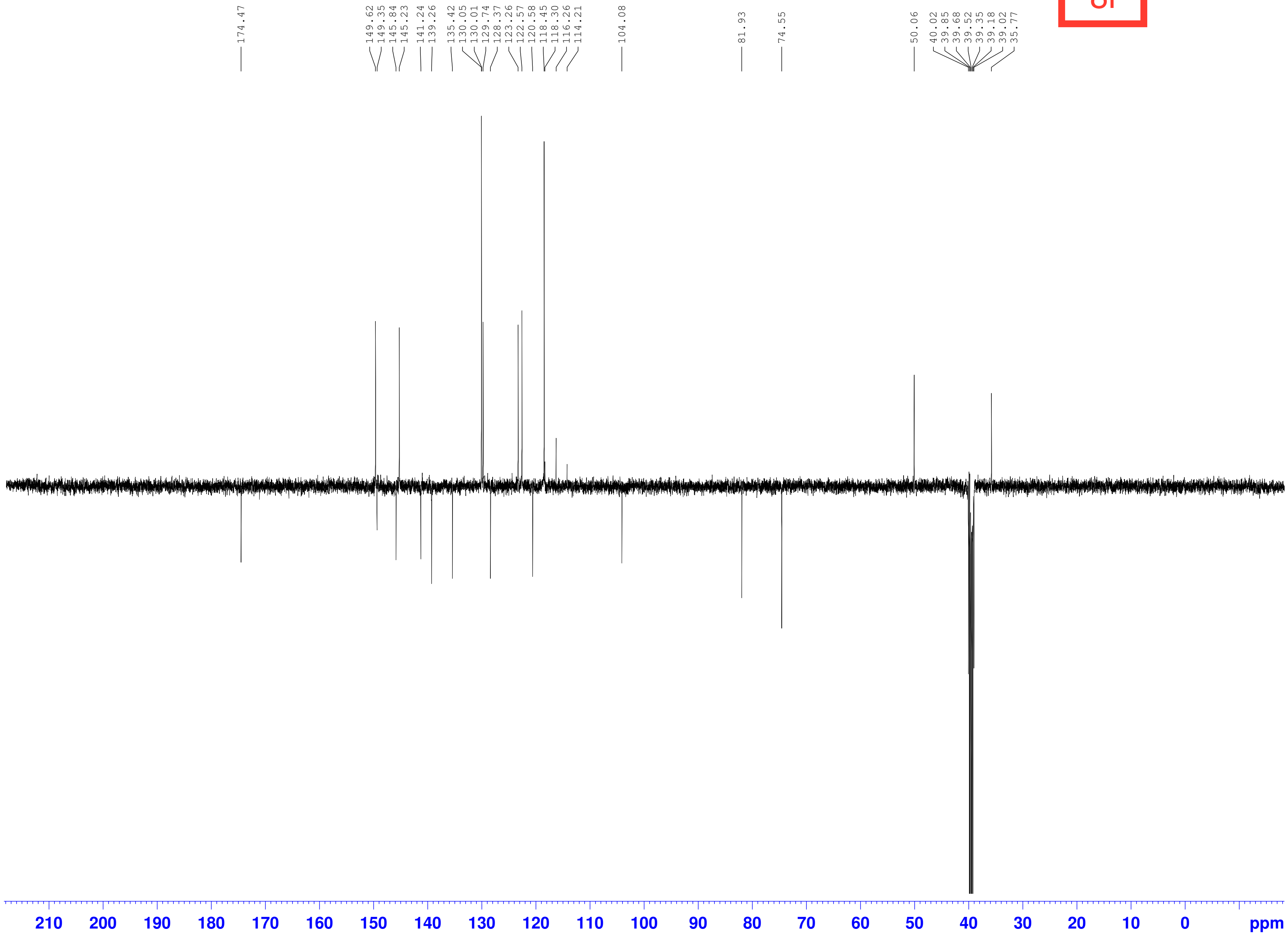

NAME	CM-SK92P	
EXPNO	7	
PROCNO	1	
Date_	20141025	
Time	17.34	
INSTRUM	Avance500	
PROBHD	5 mm QNP 1H/13	
PULPROG	pendant	
TD	65536	
SOLVENT	DMSO	
NS	3200	
DS	4	
SWH	29761.904	Hz
FIDRES	0.454131	Hz
AQ	1.1010548	sec
RG	3250	
DW	16.800	usec
DE	12.00	usec
TE	298.1	K
CNST2	145.0000000	
D1	2.00000000	sec
D4	0.00172414	sec
D12	0.00002000	sec
D15	0.00431034	sec
D20	0.00345000	sec
TD0	25	

======== CHANNEL f1 ========

NUC1	13C	
P1	7.20	usec
P2	14.40	usec
PL1	-2.00	dB
PL1W	101.27846527	W
SFO1	125.7703643	MHz

======== CHANNEL f2 ========

CPDPRG2	waltz16	
NUC2	1H	
P3	11.50	usec
P4	23.00	usec
PCPD2	80.00	usec
PL2	-2.00	dB
PL12	14.85	dB
PL2W	14.33185768	W
PL12W	0.29600734	W
SFO2	500.1320005	MHz
SI	32768	
SF	125.7578519	MHz
WDW	EM	
SSB	0	
LB	1.00	Hz
GB	0	
PC	1.40	

SK92P MW=449?
(MeOH)/MeOH + NH4OAc
C24H17F2N3O4

EPSRC National Facility Swansea
LTQ Orbitrap XL

Dr AD Westwell
05/08/2015 16:37:23

8l

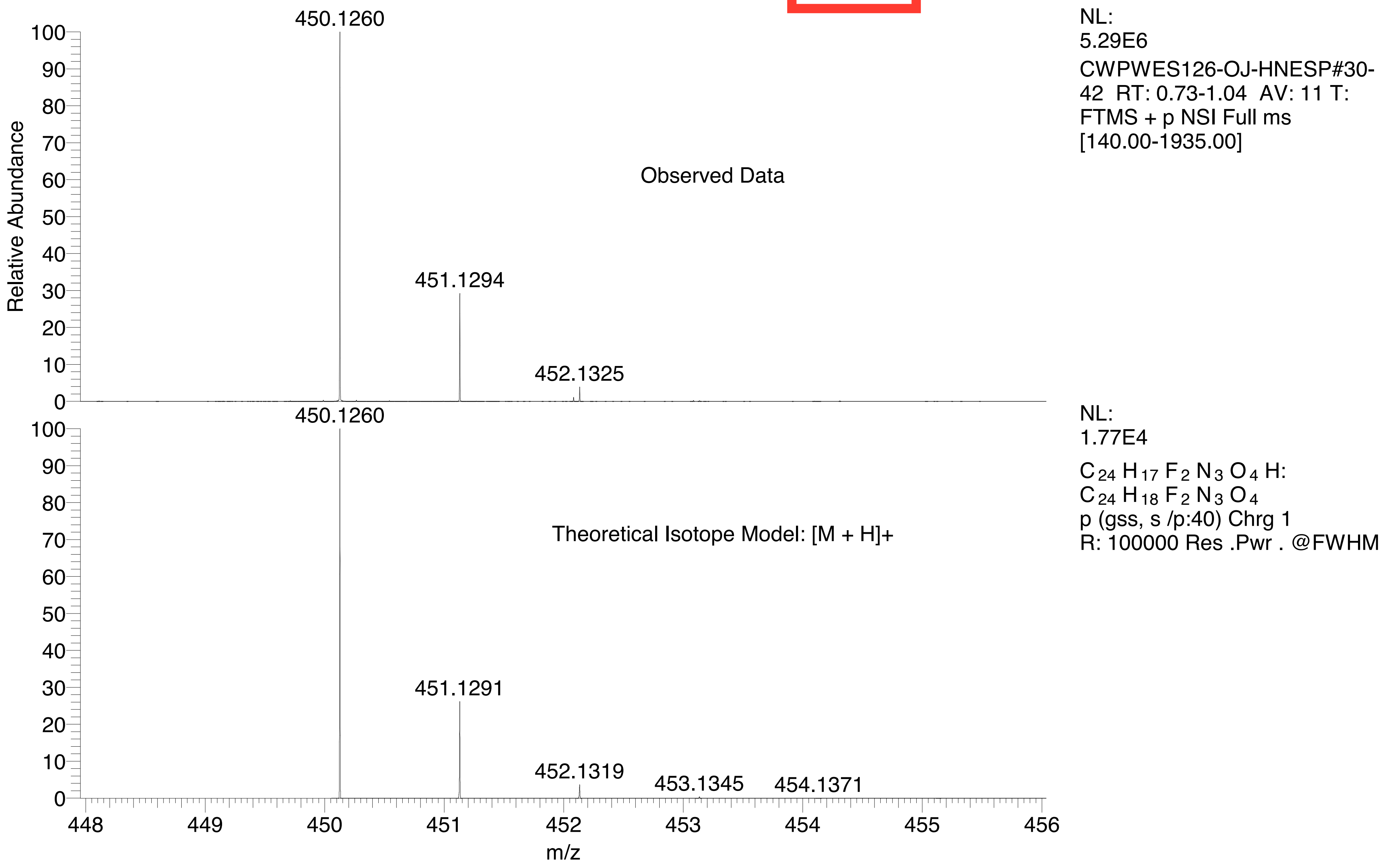

8m

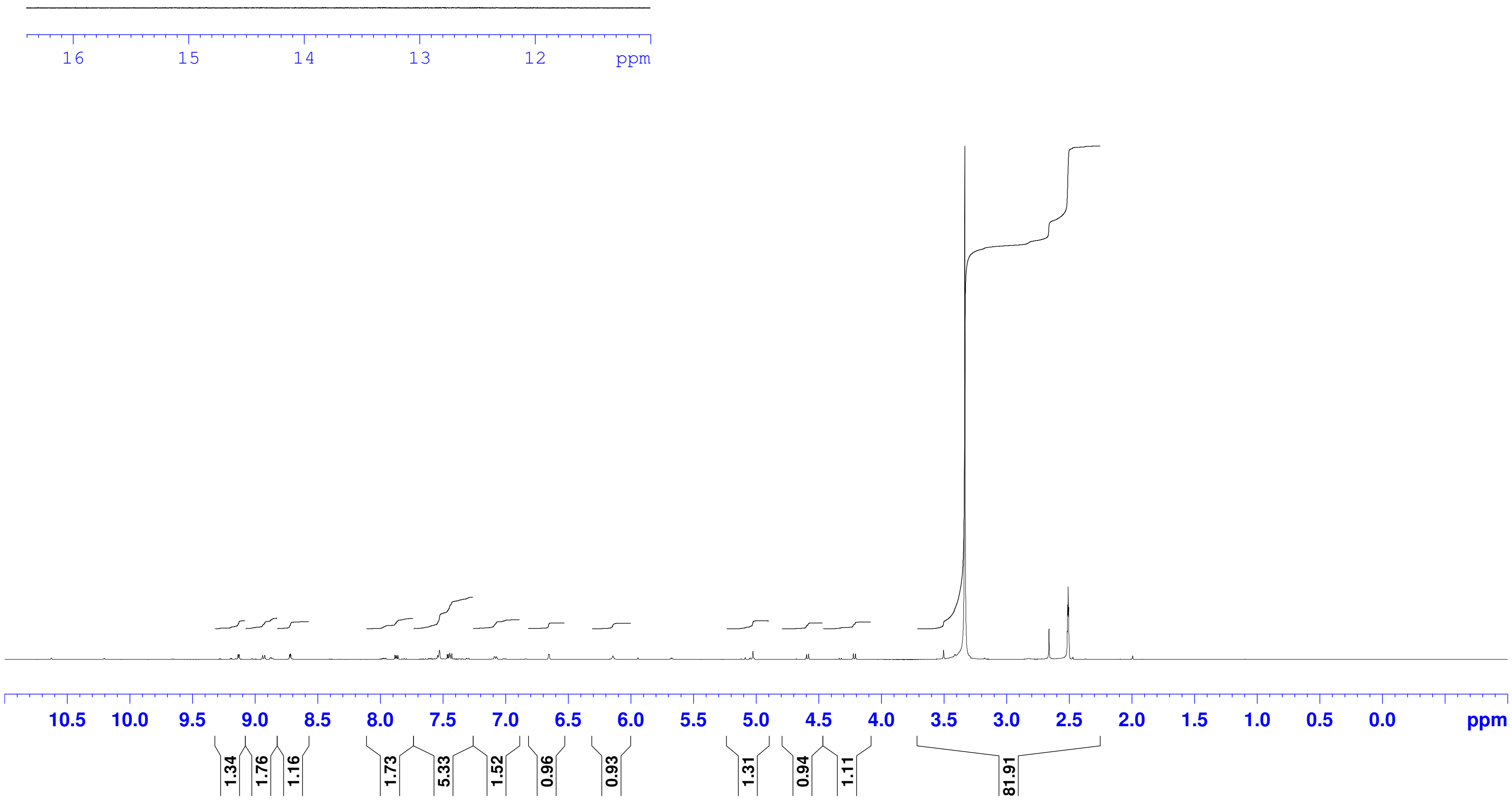

CARDIFF UNIVERSITY PRIFYSGOL CAERDYDD

NAME	CM-SK93P
EXPNO	16
PROCNO	1
Date_	20141027
Time	8.27
INSTRUM	Avance500
PROBHD	5 mm QNP 1H/13
PULPROG	zg30
TD	65536
SOLVENT	DMSO
NS	16
DS	2
SWH	10330.578 Hz
FIDRES	0.157632 Hz
AQ	3.1719923 sec
RG	362
DW	48.400 use
DE	6.50 use
TE	298.1 K
D1	1.00000000 sec
TD0	1

======== CHANNEL f1 =======

NUC1	1H
P1	11.50 use
PL1	-1.00 dB
PL1W	11.38419914 W
SFO1	500.1330885 MHz
SI	32768
SF	500.1300000 MHz
WDW	EM
SSB	0
LB	0.30 Hz
GB	0
PC	1.00

8m

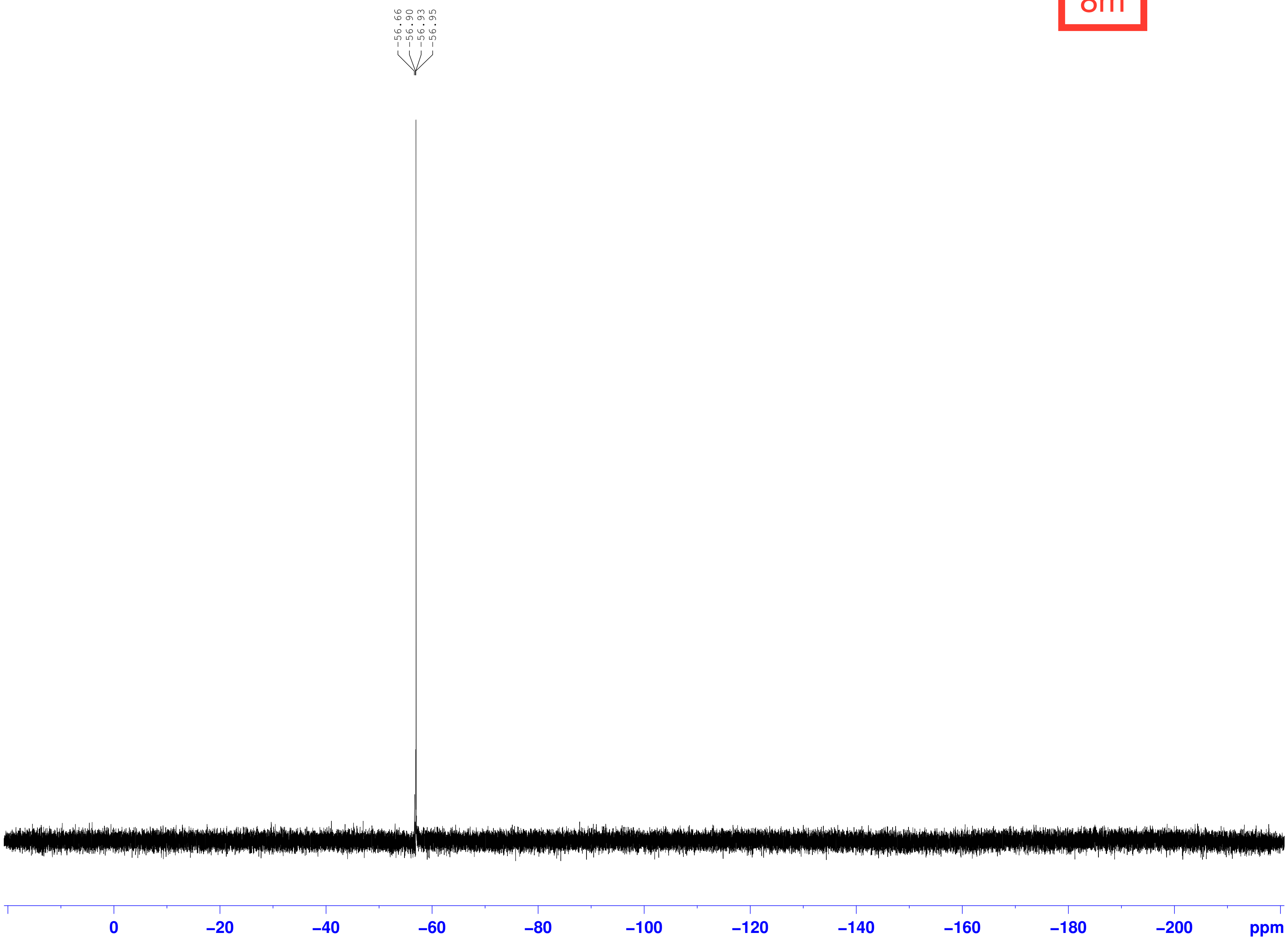

CARDIFF UNIVERSITY
PRIFYSGOL CAERDYDD

NAME	CM-SK93P	
EXPNO	6	
PROCNO	1	
Date_	20141015	
Time	15.46	
INSTRUM	Avance500	
PROBHD	5 mm QNP 1H/13	
PULPROG	zgfhigqn	
TD	131072	
SOLVENT	DMSO	
NS	16	
DS	4	
SWH	113636.367	Hz
FIDRES	0.866977	Hz
AQ	0.5767668	sec
RG	4100	
DW	4.400	use
DE	6.00	use
TE	298.1	K
D1	1.00000000	sec
D11	0.03000000	sec
D12	0.00002000	sec
TD0	1	
======== CHANNEL f1 =======		
NUC1	19F	
P1	18.60	use
PL1	-1.50	dB
PL1W	11.14113998	W
SFO1	470.5453180	MHz
======== CHANNEL f2 =======		
CPDPRG2	waltz16	
NUC2	1H	
PCPD2	80.00	use
PL2	-2.00	dB
PL12	14.85	dB
PL2W	14.33185768	W
PL12W	0.29600734	W
SFO2	500.1320005	MHz
SI	65536	
SF	470.5923770	MHz
WDW	EM	
SSB	0	
LB	0.30	Hz
GB	0	
PC	1.40	

8m

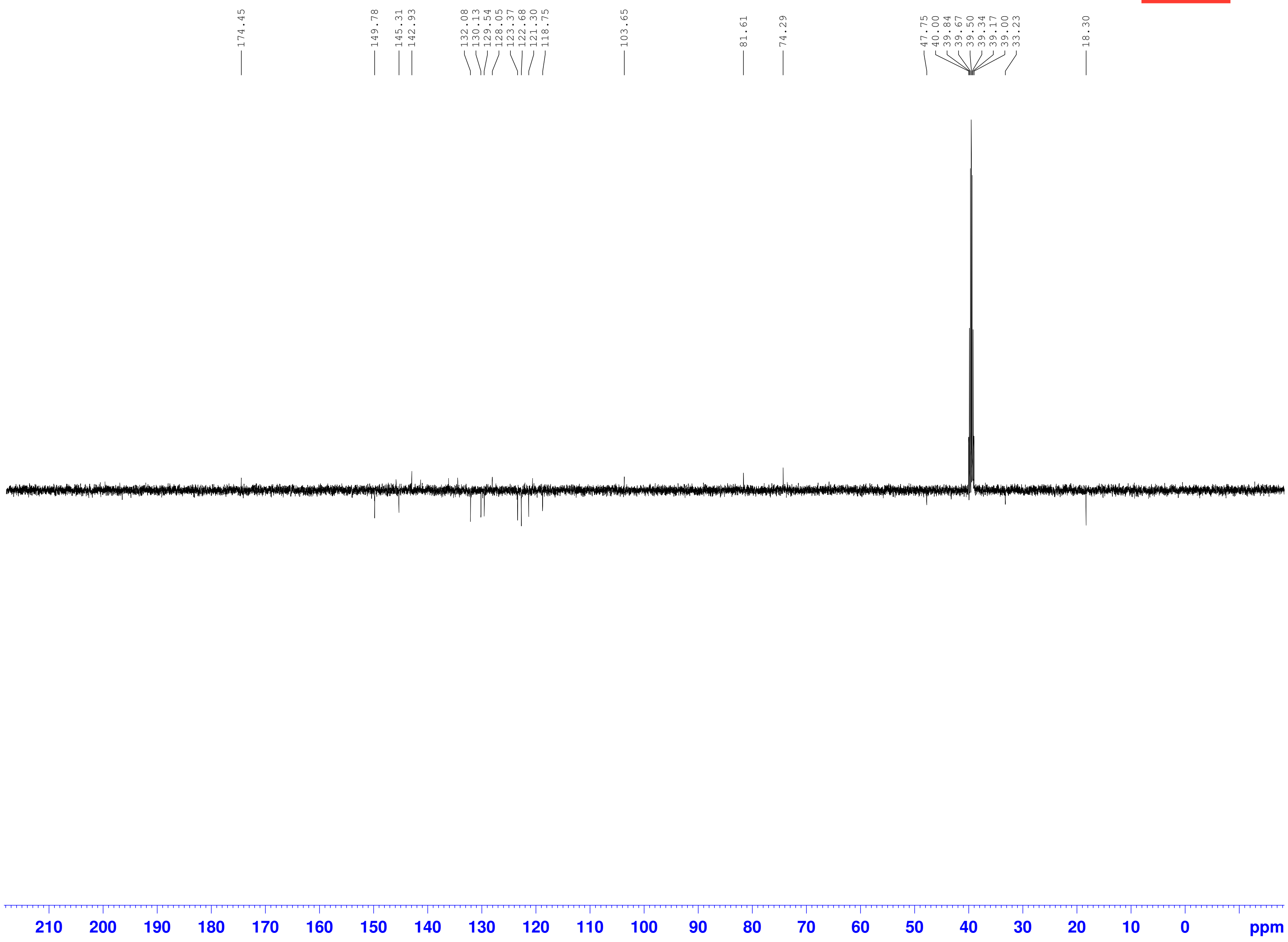

CARDIFF UNIVERSITY PRIFYSGOL CAERDYDD

```
NAME          CM-SK93P
EXPNO               15
PROCNO               1
Date_         20141027
Time              7.44
INSTRUM      Avance500
PROBHD   5 mm QNP 1H/13
PULPROG        pendant
TD               65536
SOLVENT           DMSO
NS                 512
DS                   4
SWH          29761.904 Hz
FIDRES        0.454131 Hz
AQ           1.1010548 sec
RG                3250
DW              16.800 usec
DE               12.00 usec
TE               298.1 K
CNST2      145.0000000
D1          2.00000000 sec
D4          0.00172414 sec
D12         0.00002000 sec
D15         0.00431034 sec
D20         0.00345000 sec
TD0                  4

======== CHANNEL f1 ========
NUC1               13C
P1                7.20 usec
P2               14.40 usec
PL1              -2.00 dB
PL1W      101.27846527 W
SFO1       125.7703643 MHz

======== CHANNEL f2 ========
CPDPRG2        waltz16
NUC2                1H
P3               11.50 usec
P4               23.00 usec
PCPD2            80.00 usec
PL2              -2.00 dB
PL12             14.85 dB
PL2W       14.33185768 W
PL12W       0.29600734 W
SFO2       500.1320005 MHz
SI               32768
SF         125.7578519 MHz
WDW                 EM
SSB                  0
LB                1.00 Hz
GB                   0
PC                1.40
```

8m

MEDAC LTD

Analytical and chemical consultancy services

MEDAC Ltd
Alpha 319
Chobham Business Centre
Chertsey Road
Chobham
Surrey
GU24 8JB
United Kingdom

www.medacltd.com
Tel/Fax No. 01276 855410
Email: info@medacltd.com

A N A L Y T I C A L   R E P O R T

Date 29th July 2015

Name Dr Andrew Westwell

Sample ID SK93P

Formula $C_{25}H_{18}F_3N_3O_4$

ELEMENT	C	H	N						
**% Theory**	62.37	3.77	8.72						
**% Found 1**	61.91	3.43	9.13						
**% Found 2**	61.98	3.56	8.91						

Comments:

Assay No: 154659

Analyst: Richard Morris

8n

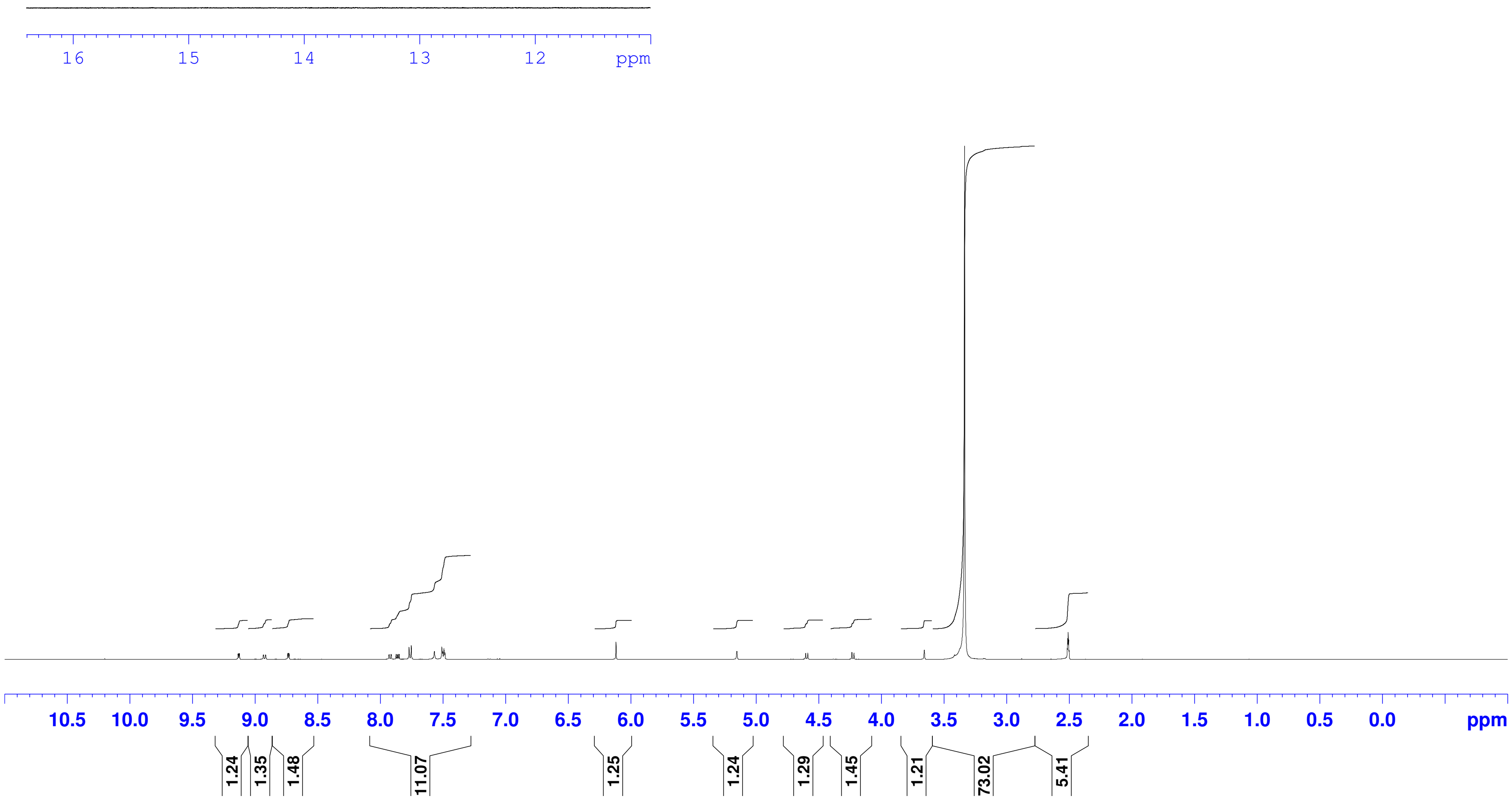

NAME	CM-SK94P	
EXPNO	8	
PROCNO	1	
Date_	20141010	
Time	8.18	
INSTRUM	Avance500	
PROBHD	5 mm QNP 1H/13	
PULPROG	zg30	
TD	65536	
SOLVENT	DMSO	
NS	16	
DS	2	
SWH	10330.578	Hz
FIDRES	0.157632	Hz
AQ	3.1719923	sec
RG	406	
DW	48.400	use
DE	6.50	use
TE	298.1	K
D1	1.00000000	sec
TD0	1	

======== CHANNEL f1 =======

NUC1	1H	
P1	11.50	use
PL1	-1.00	dB
PL1W	11.38419914	W
SFO1	500.1330885	MHz
SI	32768	
SF	500.1300000	MHz
WDW	EM	
SSB	0	
LB	0.30	Hz
GB	0	
PC	1.00	

8n

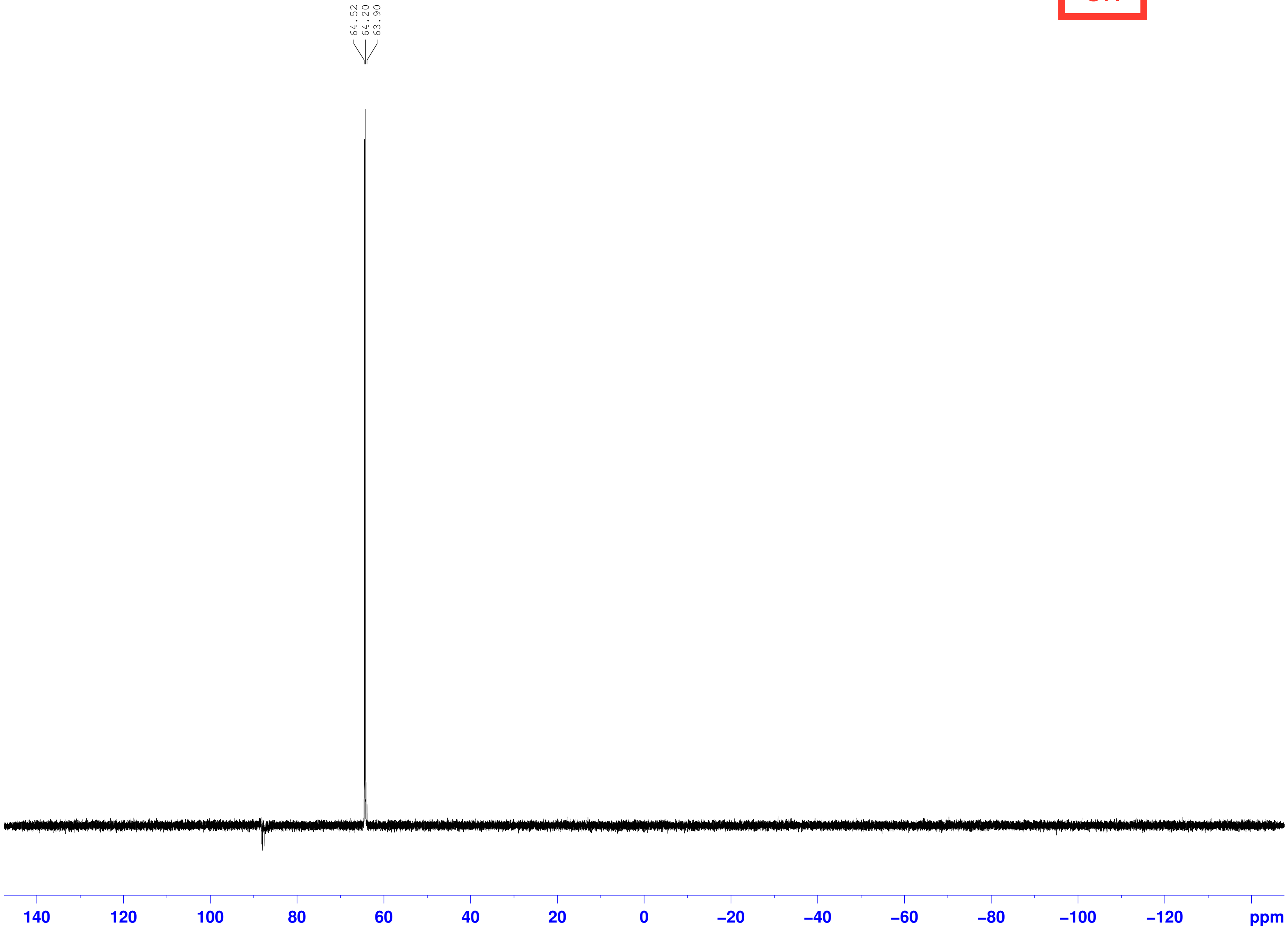

NAME	CM-SK94P
EXPNO	3
PROCNO	1
Date_	20140922
Time	9.43
INSTRUM	Avance500
PROBHD	5 mm QNP 1H/13
PULPROG	zgfhigqn
TD	131072
SOLVENT	DMSO
NS	16
DS	4
SWH	138888.891 Hz
FIDRES	1.059638 Hz
AQ	0.4719092 sec
RG	2300
DW	3.600 use
DE	6.00 use
TE	288.8 K
D1	1.00000000 sec
D11	0.03000000 sec
D12	0.00002000 sec
TD0	1

======== CHANNEL f1 =======

NUC1	19F
P1	18.60 use
PL1	-1.50 dB
PL1W	11.14113998 W
SFO1	470.5923770 MHz

======== CHANNEL f2 =======

CPDPRG2	waltz16
NUC2	1H
PCPD2	80.00 use
PL2	-2.00 dB
PL12	14.85 dB
PL2W	14.33185768 W
PL12W	0.29600734 W
SFO2	500.1320005 MHz
SI	65536
SF	470.5923770 MHz
WDW	EM
SSB	0
LB	0.30 Hz
GB	0
PC	1.40

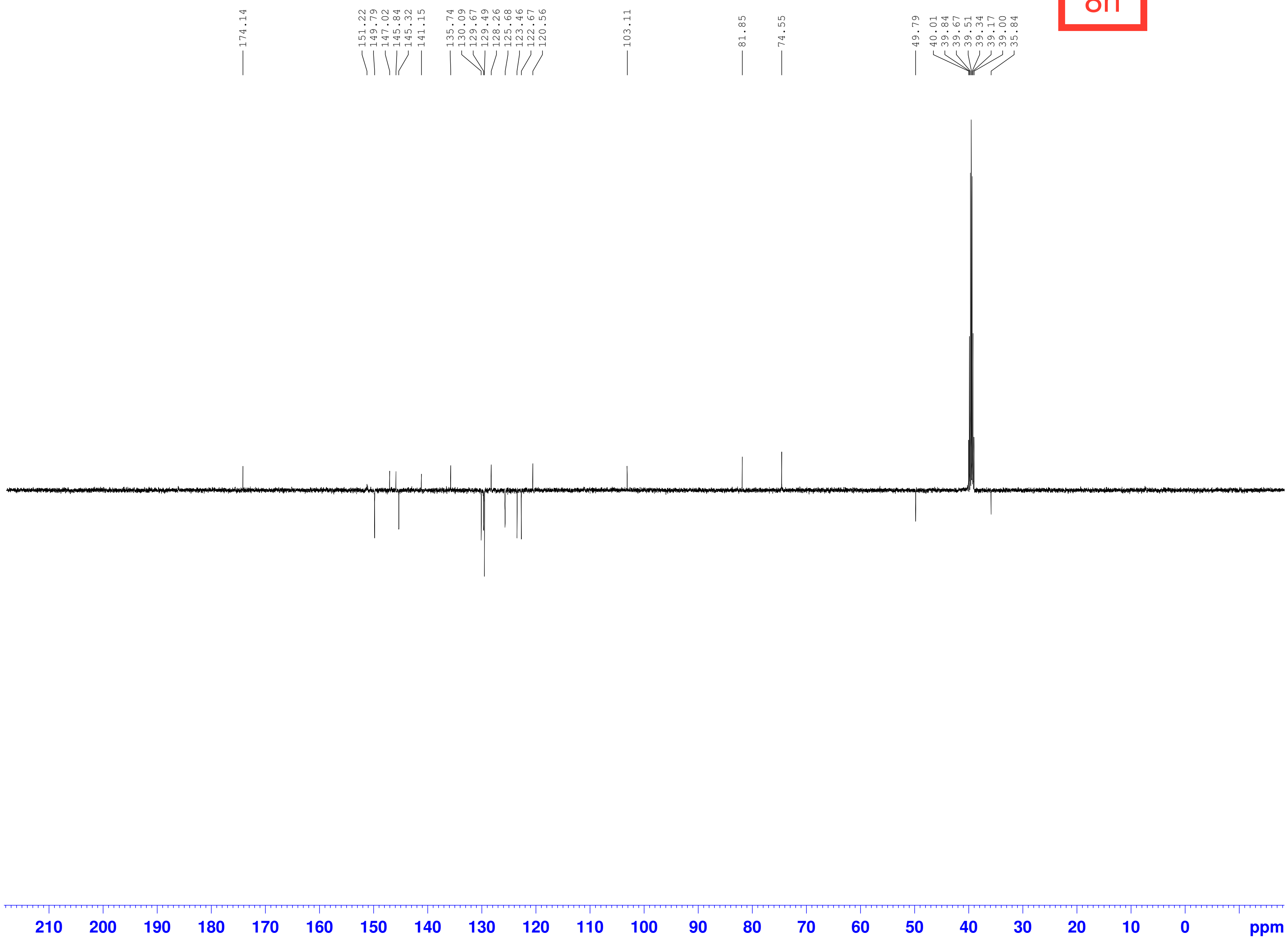

8n

NAME	CM-SK94P
EXPNO	7
PROCNO	1
Date_	20141010
Time	5.43
INSTRUM	Avance500
PROBHD	5 mm QNP 1H/13
PULPROG	pendant
TD	65536
SOLVENT	DMSO
NS	3072
DS	4
SWH	29761.904 Hz
FIDRES	0.454131 Hz
AQ	1.1010548 sec
RG	3250
DW	16.800 usec
DE	12.00 usec
TE	298.1 K
CNST2	145.0000000
D1	2.00000000 sec
D4	0.00172414 sec
D12	0.00002000 sec
D15	0.00431034 sec
D20	0.00345000 sec
TD0	24
======== CHANNEL f1 ========	
NUC1	13C
P1	7.20 usec
P2	14.40 usec
PL1	-2.00 dB
PL1W	101.27846527 W
SFO1	125.7703643 MHz
======== CHANNEL f2 ========	
CPDPRG2	waltz16
NUC2	1H
P3	11.50 usec
P4	23.00 usec
PCPD2	80.00 usec
PL2	-2.00 dB
PL12	14.85 dB
PL2W	14.33185768 W
PL12W	0.29600734 W
SFO2	500.1320005 MHz
SI	32768
SF	125.7578519 MHz
WDW	EM
SSB	0
LB	1.00 Hz
GB	0
PC	1.40

8n

MEDAC LTD

Analytical and chemical consultancy services

MEDAC Ltd
Alpha 319
Chobham Business Centre
Chertsey Road
Chobham
Surrey
GU24 8JB
United Kingdom

www.medacltd.com
Tel/Fax No. 01276 855410
Email: info@medacltd.com

A N A L Y T I C A L R E P O R T

Date 21st July 2015

Name Dr Andrew Westwell

Sample ID 5K94P

Formula $C_{23}H_{16}F_5N_3O_3S$

ELEMENT	C	H	N						
% Theory	54.23	3.17	8.24						
% Found 1	53.96	2.98	8.42						
% Found 2	53.93	3.14	8.33						

Comments: All sample used.

Assay No: 154496

Analyst: Richard Morris

CARDIFF UNIVERSITY
PRIFYSGOL CAERDYDD

8o

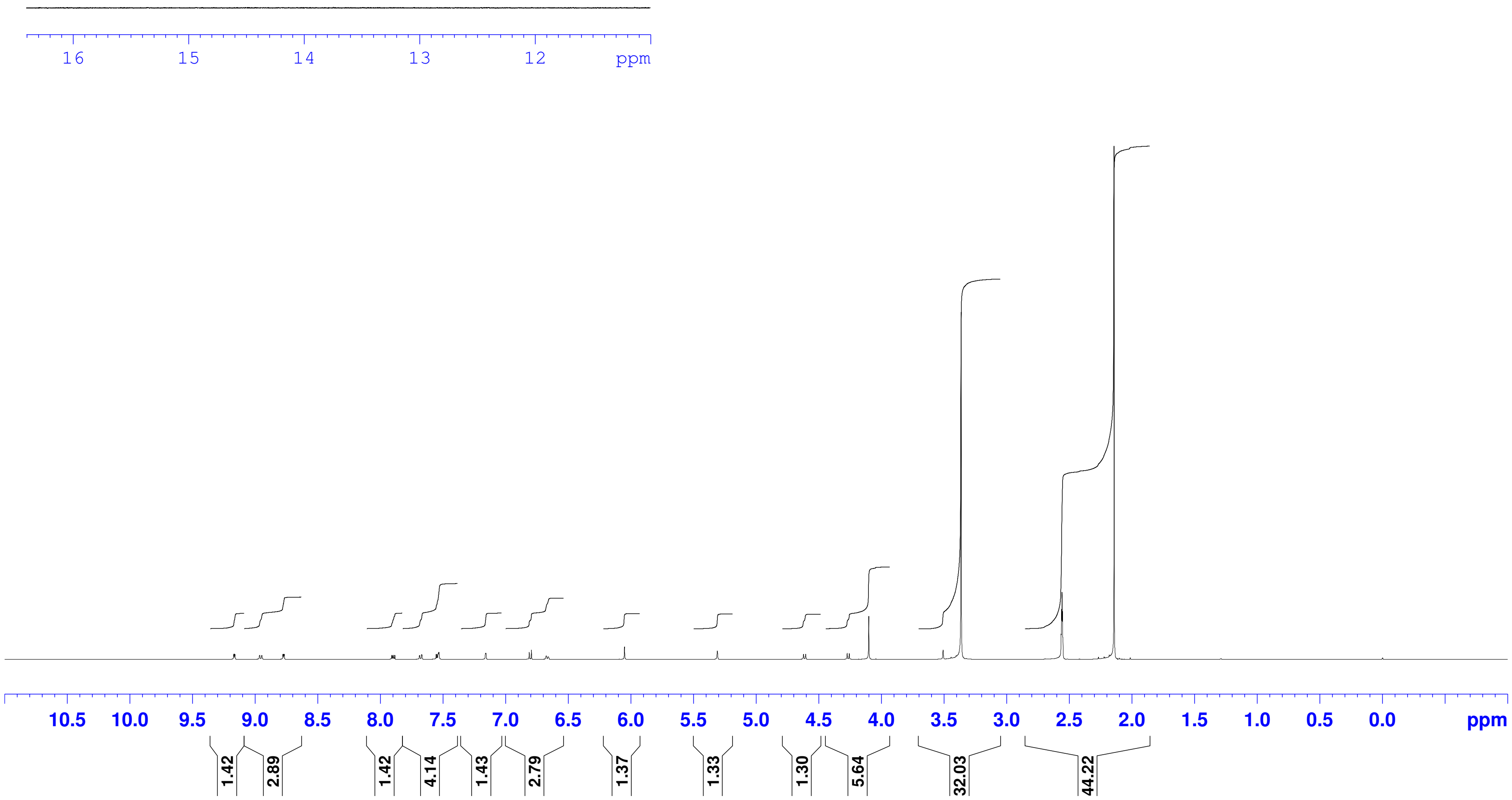

NAME	CM-SK95P
EXPNO	5
PROCNO	1
Date_	20141015
Time	13.31
INSTRUM	Avance500
PROBHD	5 mm QNP 1H/13
PULPROG	zg30
TD	65536
SOLVENT	DMSO
NS	16
DS	2
SWH	10330.578 Hz
FIDRES	0.157632 Hz
AQ	3.1719923 sec
RG	406
DW	48.400 use
DE	6.50 use
TE	298.1 K
D1	1.00000000 sec
TD0	1

======== CHANNEL f1 =======

NUC1	1H
P1	11.50 use
PL1	-1.00 dB
PL1W	11.38419914 W
SFO1	500.1330885 MHz
SI	32768
SF	500.1299754 MHz
WDW	EM
SSB	0
LB	0.30 Hz
GB	0
PC	1.00

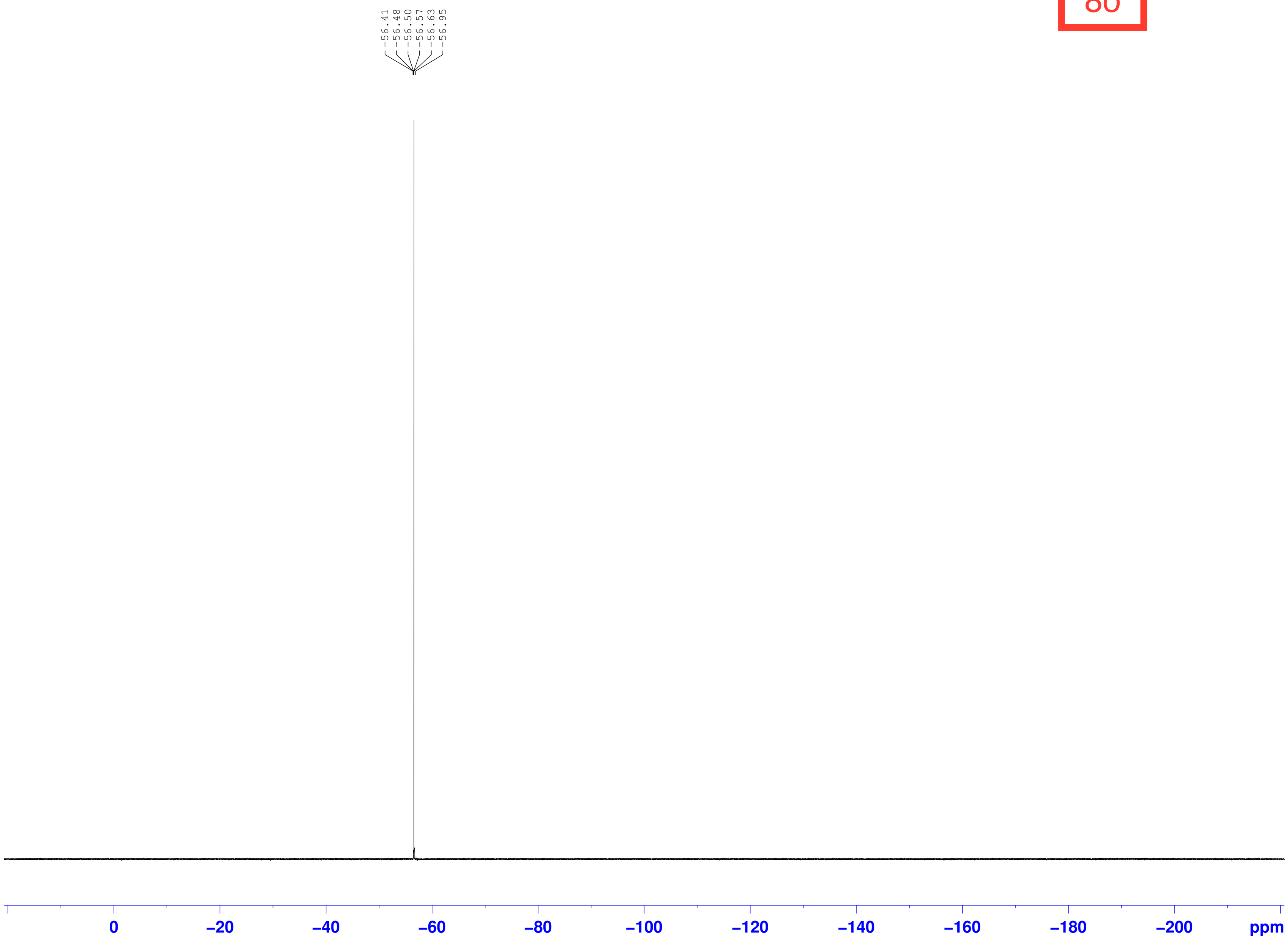

8o

CARDIFF UNIVERSITY
PRIFYSGOL CAERDYDD

NAME	CM-SK95P
EXPNO	7
PROCNO	1
Date_	20141015
Time	13.37
INSTRUM	Avance500
PROBHD	5 mm QNP 1H/13
PULPROG	zgfhigqn
TD	131072
SOLVENT	DMSO
NS	16
DS	4
SWH	113636.367 Hz
FIDRES	0.866977 Hz
AQ	0.5767668 sec
RG	4100
DW	4.400 use
DE	6.00 use
TE	298.2 K
D1	1.00000000 sec
D11	0.03000000 sec
D12	0.00002000 sec
TD0	1

======== CHANNEL f1 =======

NUC1	19F
P1	18.60 use
PL1	-1.50 dB
PL1W	11.14113998 W
SFO1	470.5453180 MHz

======== CHANNEL f2 =======

CPDPRG2	waltz16
NUC2	1H
PCPD2	80.00 use
PL2	-2.00 dB
PL12	14.85 dB
PL2W	14.33185768 W
PL12W	0.29600734 W
SFO2	500.1320005 MHz
SI	65536
SF	470.5923770 MHz
WDW	EM
SSB	0
LB	0.30 Hz
GB	0
PC	1.40

8o

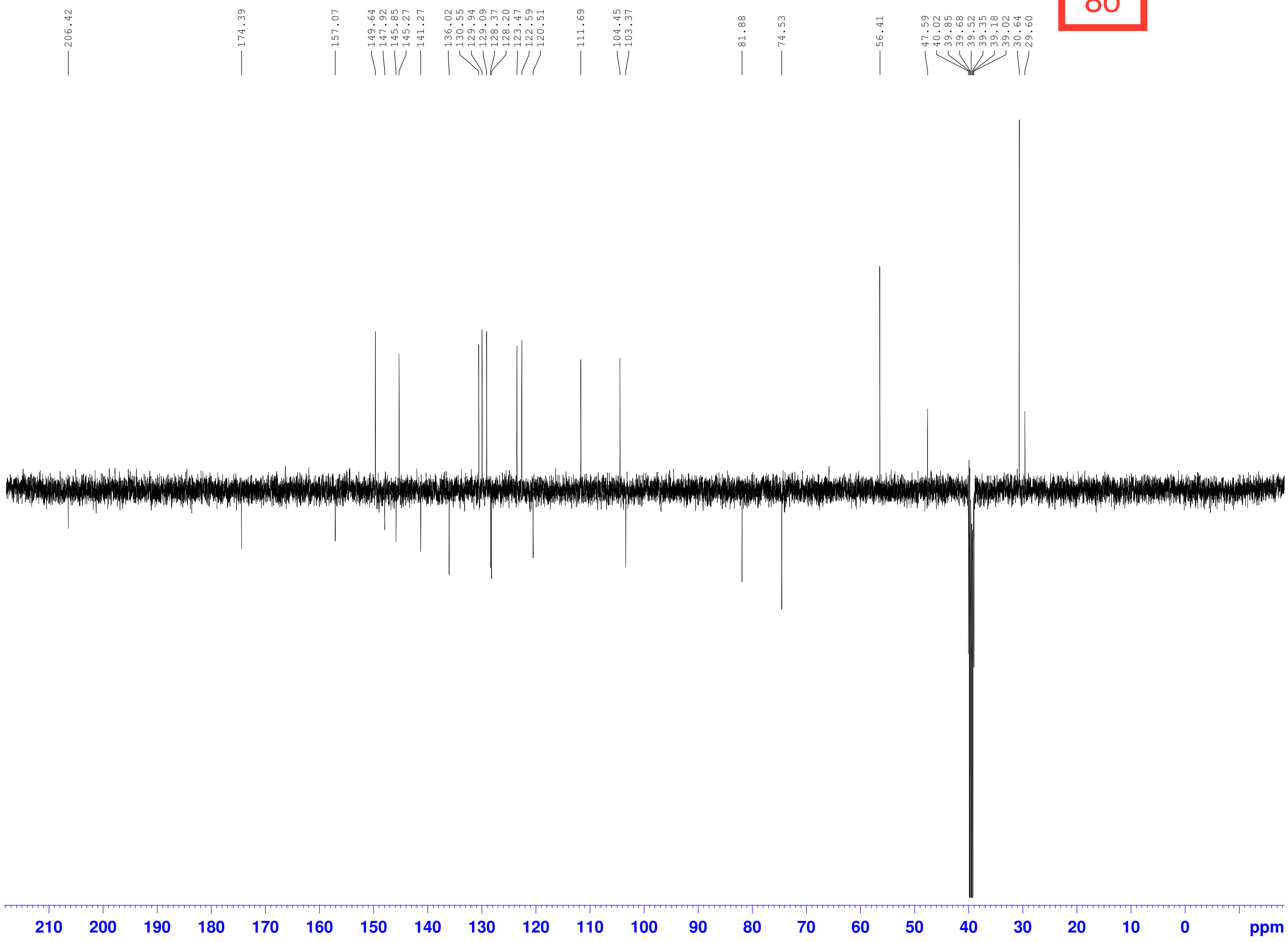

NAME	CM-SK95P
EXPNO	8
PROCNO	1
Date_	20141021
Time	23.08
INSTRUM	Avance500
PROBHD	5 mm QNP 1H/13
PULPROG	pendant
TD	65536
SOLVENT	DMSO
NS	1024
DS	4
SWH	29761.904 Hz
FIDRES	0.454131 Hz
AQ	1.1010548 sec
RG	3250
DW	16.800 usec
DE	12.00 usec
TE	298.1 K
CNST2	145.0000000
D1	2.00000000 sec
D4	0.00172414 sec
D12	0.00002000 sec
D15	0.00431034 sec
D20	0.00345000 sec
TD0	8

======== CHANNEL f1 ========

NUC1	13C
P1	7.20 usec
P2	14.40 usec
PL1	-2.00 dB
PL1W	101.27846527 W
SFO1	125.7703643 MHz

======== CHANNEL f2 ========

CPDPRG2	waltz16
NUC2	1H
P3	11.50 usec
P4	23.00 usec
PCPD2	80.00 usec
PL2	-2.00 dB
PL12	14.85 dB
PL2W	14.33185768 W
PL12W	0.29600734 W
SFO2	500.1320005 MHz
SI	32768
SF	125.7578519 MHz
WDW	EM
SSB	0
LB	1.00 Hz
GB	0
PC	1.40

SK95P MW=497?
(MeOH)/MeOH + NH4OAc
C25H18F3N3O5

EPSRC National Facility Swansea
LTQ Orbitrap XL

Dr AD Westwell
05/08/2015 16:34:32

8o

SM: 7G

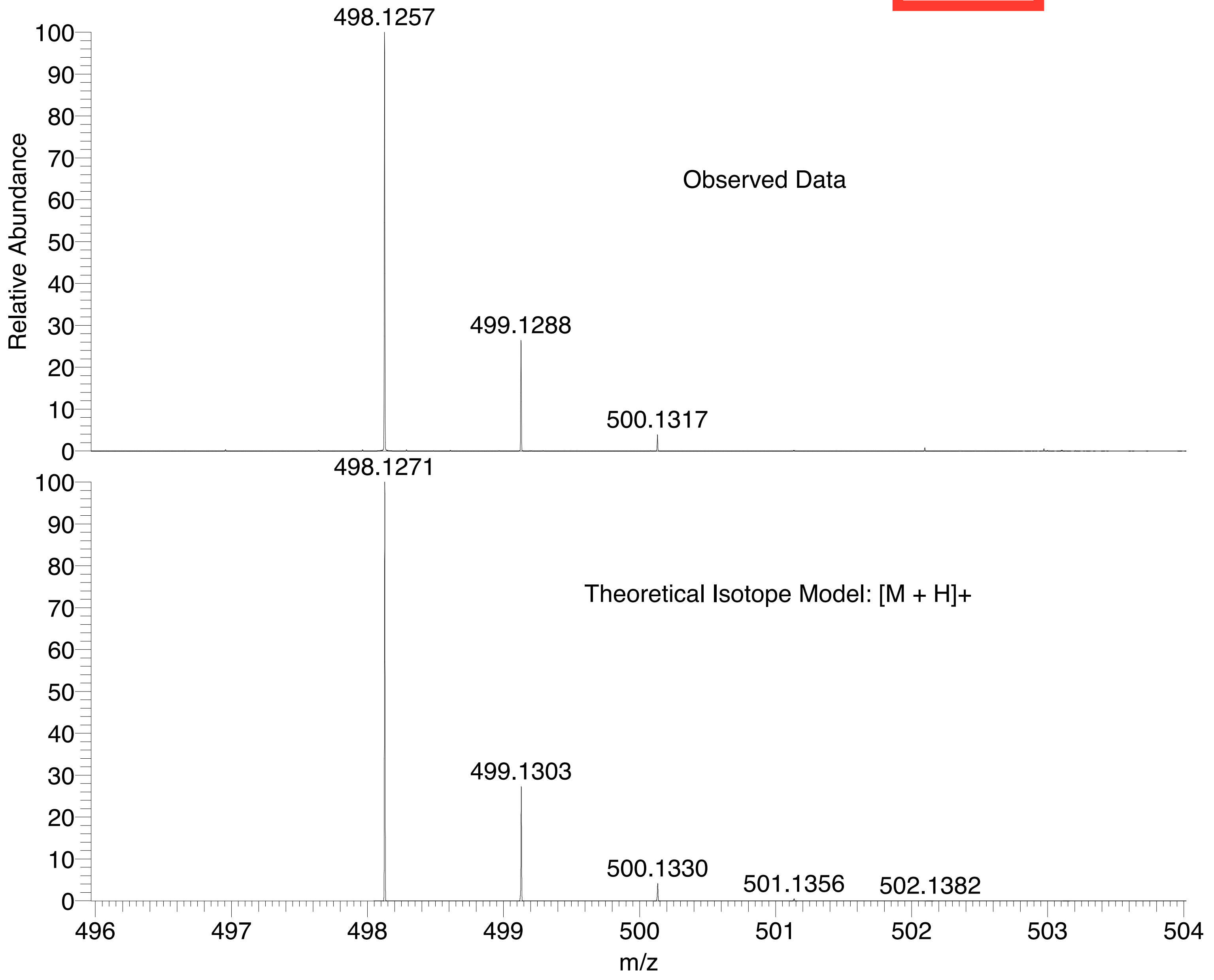

NL:
1.98E6
CWPWES125-OJ-HNESP#30-40 RT: 0.75-1.05 AV: 10 T: FTMS + p NSI Full ms [140.00-1935.00]

NL:
1.75E4
$C_{25}H_{18}F_3N_3O_5H$: $C_{25}H_{19}F_3N_3O_5$
p (gss, s /p:40) Chrg 1
R: 100000 Res .Pwr . @FWHM

CARDIFF UNIVERSITY
PRIFYSGOL CAERDYDD

8p

NAME	CM-SK96P
EXPNO	4
PROCNO	1
Date_	20141031
Time	11.35
INSTRUM	Avance500
PROBHD	5 mm QNP 1H/13
PULPROG	zg30
TD	65536
SOLVENT	DMSO
NS	16
DS	2
SWH	10330.578 Hz
FIDRES	0.157632 Hz
AQ	3.1719923 sec
RG	645
DW	48.400 use
DE	6.50 use
TE	298.1 K
D1	1.00000000 sec
TD0	1

======== CHANNEL f1 =======

NUC1	1H
P1	11.50 use
PL1	-1.00 dB
PL1W	11.38419914 W
SFO1	500.1330885 MHz
SI	32768
SF	500.1300000 MHz
WDW	EM
SSB	0
LB	0.30 Hz
GB	0
PC	1.00

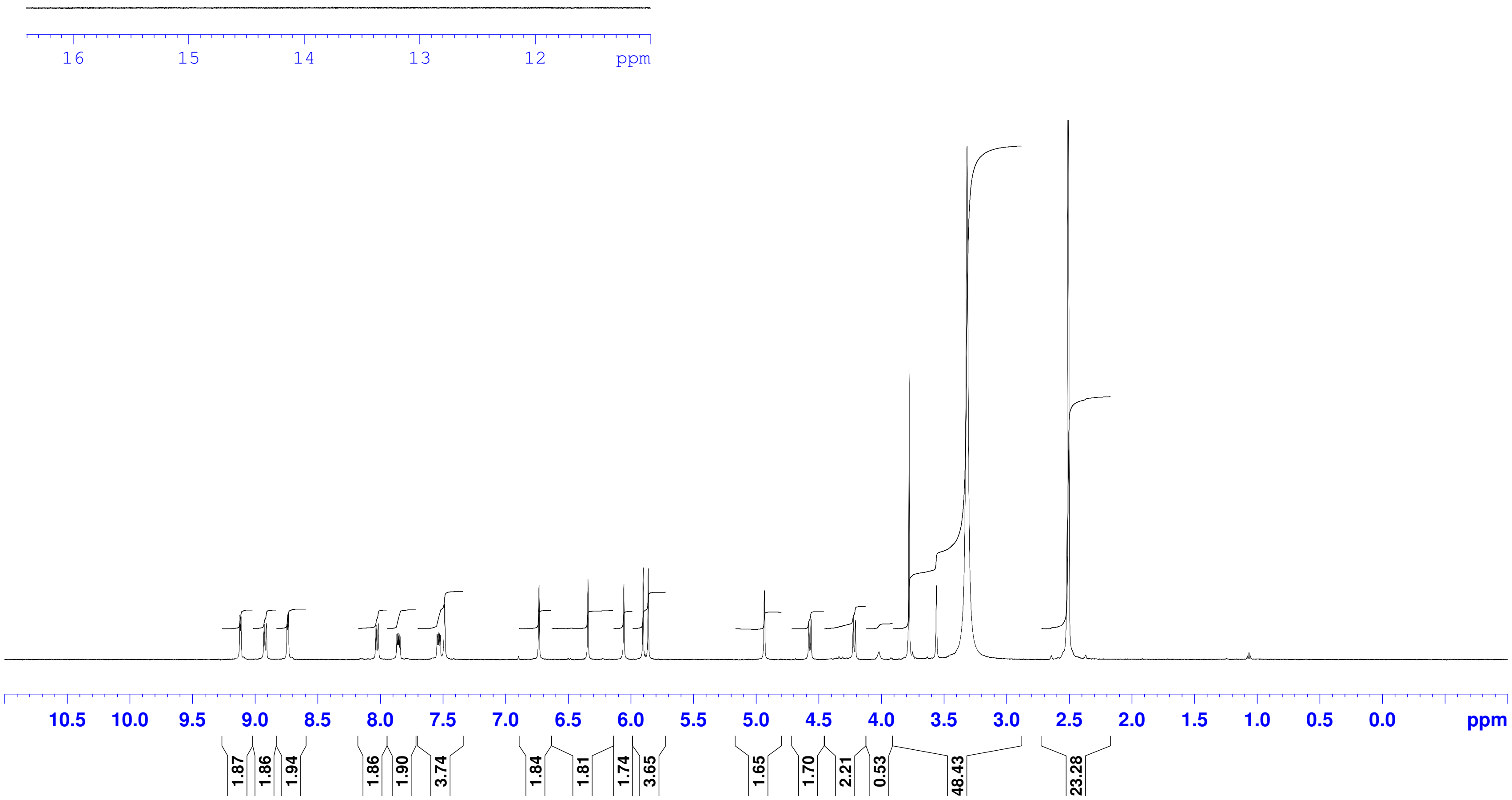

8p

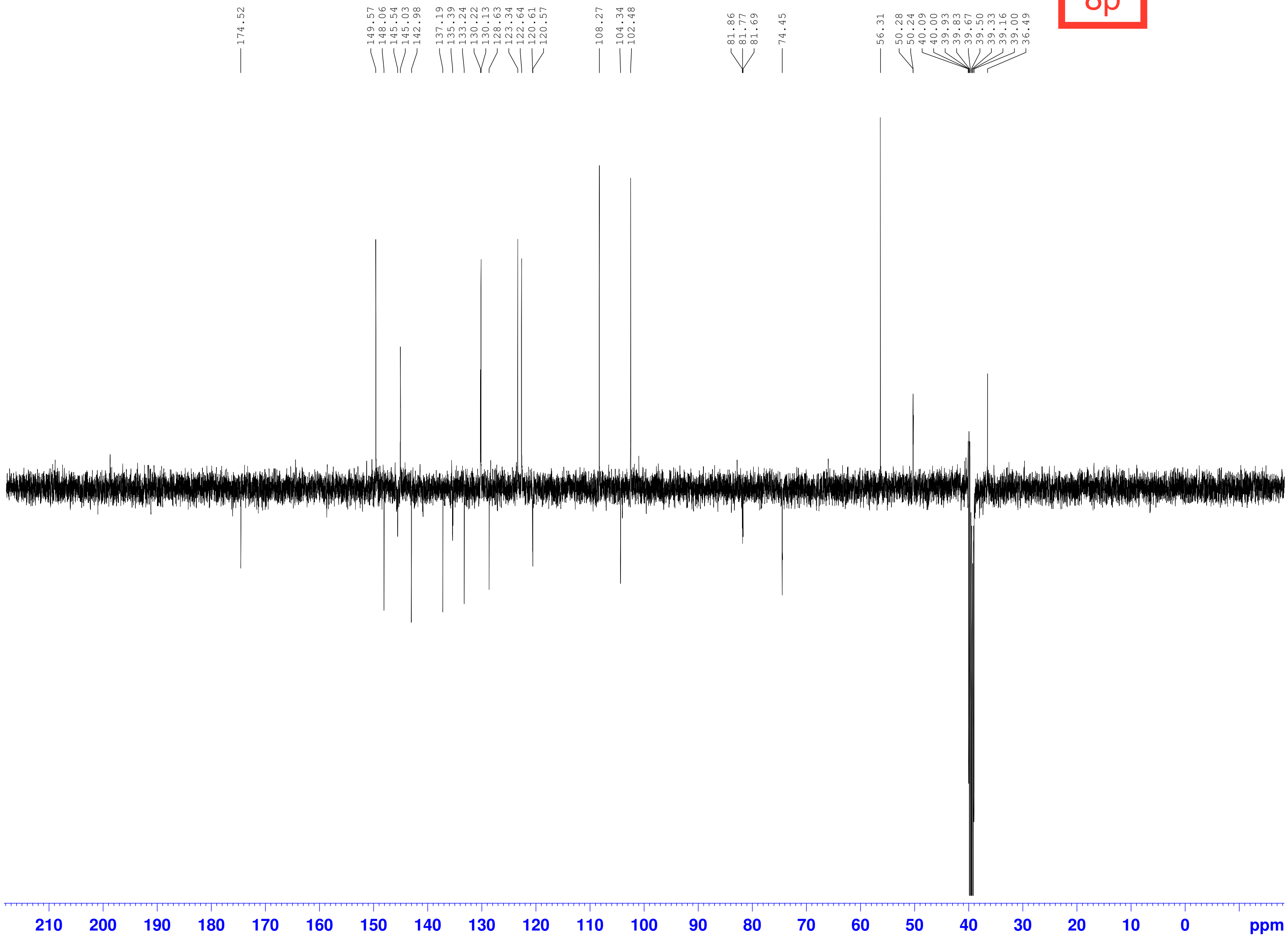

NAME	CM-SK96P	
EXPNO	7	
PROCNO	1	
Date_	20141104	
Time	5.29	
INSTRUM	Avance500	
PROBHD	5 mm QNP 1H/13	
PULPROG	pendant	
TD	65536	
SOLVENT	DMSO	
NS	2560	
DS	4	
SWH	29761.904	Hz
FIDRES	0.454131	Hz
AQ	1.1010548	sec
RG	3250	
DW	16.800	usec
DE	12.00	usec
TE	298.1	K
CNST2	145.0000000	
D1	2.00000000	sec
D4	0.00172414	sec
D12	0.00002000	sec
D15	0.00431034	sec
D20	0.00345000	sec
TD0	20	
======== CHANNEL f1 ========		
NUC1	13C	
P1	7.20	usec
P2	14.40	usec
PL1	-2.00	dB
PL1W	101.27846527	W
SFO1	125.7703643	MHz
======== CHANNEL f2 ========		
CPDPRG2	waltz16	
NUC2	1H	
P3	11.50	usec
P4	23.00	usec
PCPD2	80.00	usec
PL2	-2.00	dB
PL12	14.85	dB
PL2W	14.33185768	W
PL12W	0.29600734	W
SFO2	500.1320005	MHz
SI	32768	
SF	125.7578519	MHz
WDW	EM	
SSB	0	
LB	1.00	Hz
GB	0	
PC	1.40	

SK96P MW=457?
(MeOH)/MeOH + NH4OAc
C25H19N3O6

EPSRC National Facility Swansea
LTQ Orbitrap XL

8p

Dr AD Westwell
05/08/2015 16:31:45

SM: 7G

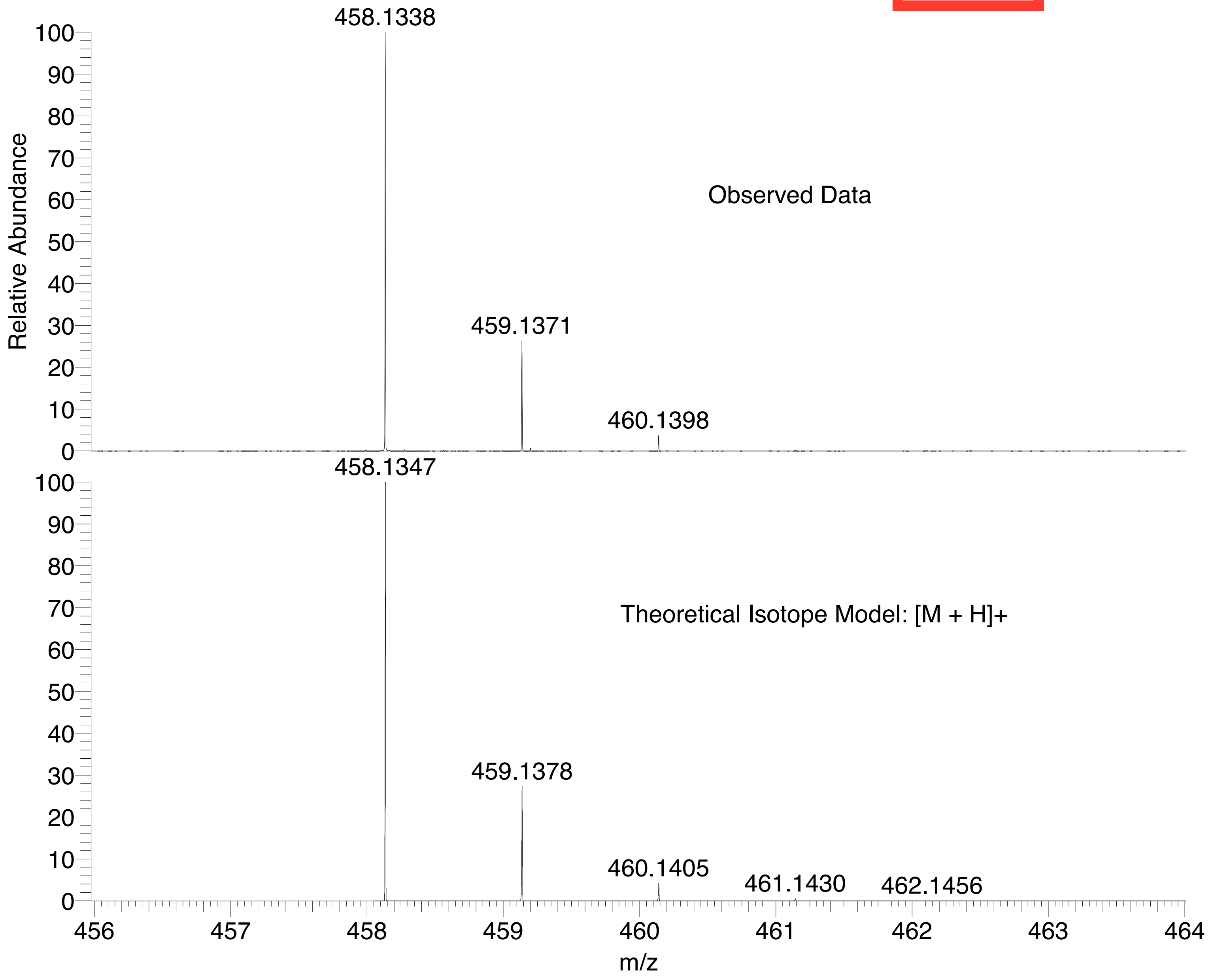

NL:
1.78E6
CWPWES124-OJ-HNESP#30-40 RT: 0.74-1.04 AV: 10 T: FTMS + p NSI Full ms [140.00-1935.00]

NL:
1.74E4
$C_{25}H_{19}N_3O_6$ H: $C_{25}H_{20}N_3O_6$
p (gss, s /p:40) Chrg 1
R: 100000 Res .Pwr . @FWHM

8q

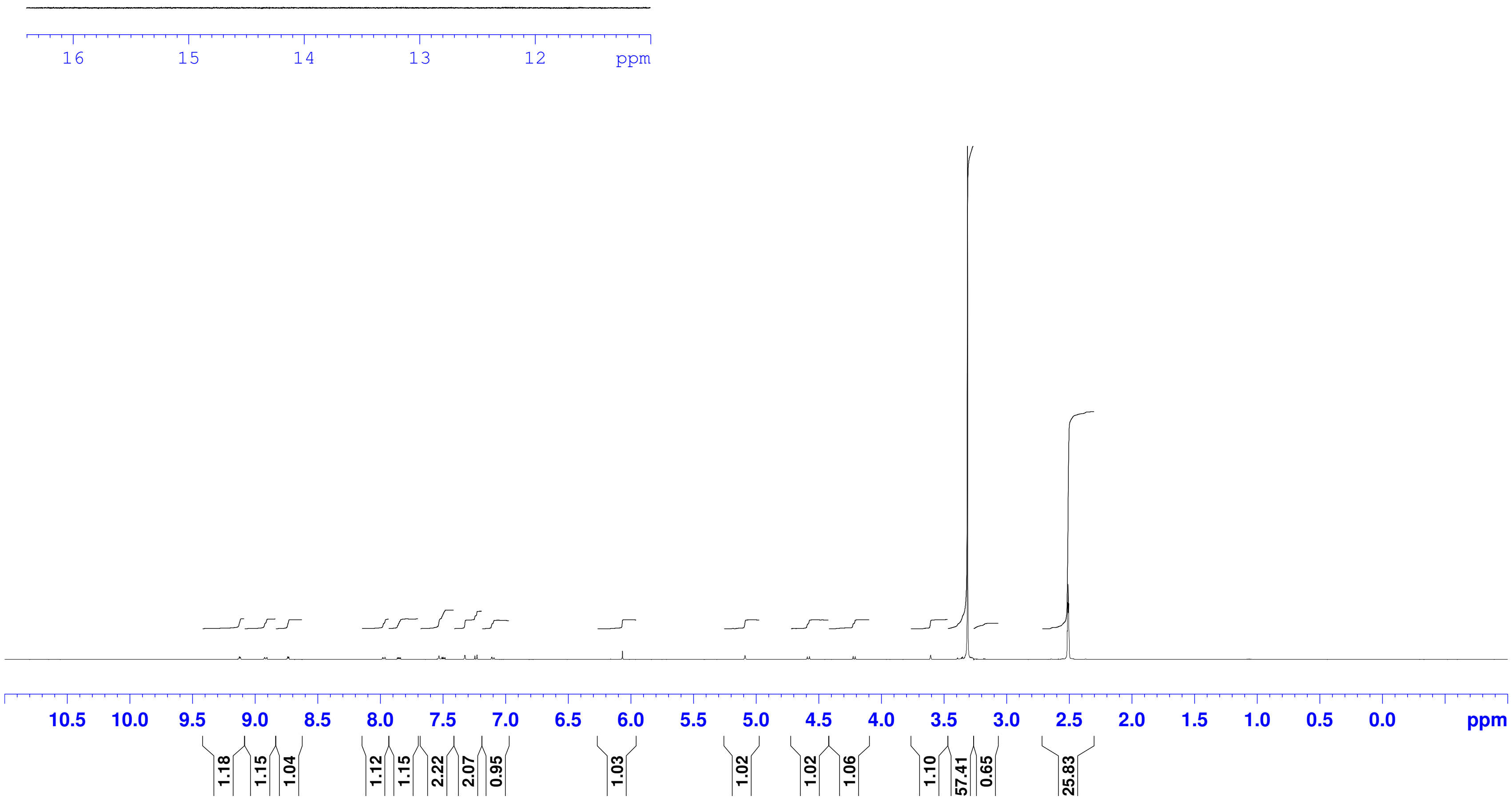

CARDIFF UNIVERSITY
PRIFYSGOL CAERDYDD

NAME	CM-SK97p
EXPNO	1
PROCNO	1
Date_	20140820
Time	13.05
INSTRUM	Avance500
PROBHD	5 mm QNP 1H/13
PULPROG	zg30
TD	65536
SOLVENT	DMSO
NS	16
DS	2
SWH	10330.578 Hz
FIDRES	0.157632 Hz
AQ	3.1719923 sec
RG	645
DW	48.400 use
DE	6.50 use
TE	298.1 K
D1	1.00000000 sec
TD0	1

======== CHANNEL f1 =======

NUC1	1H
P1	11.50 use
PL1	-1.00 dB
PL1W	11.38419914 W
SFO1	500.1330885 MHz
SI	32768
SF	500.1300000 MHz
WDW	EM
SSB	0
LB	0.30 Hz
GB	0
PC	1.00

8q

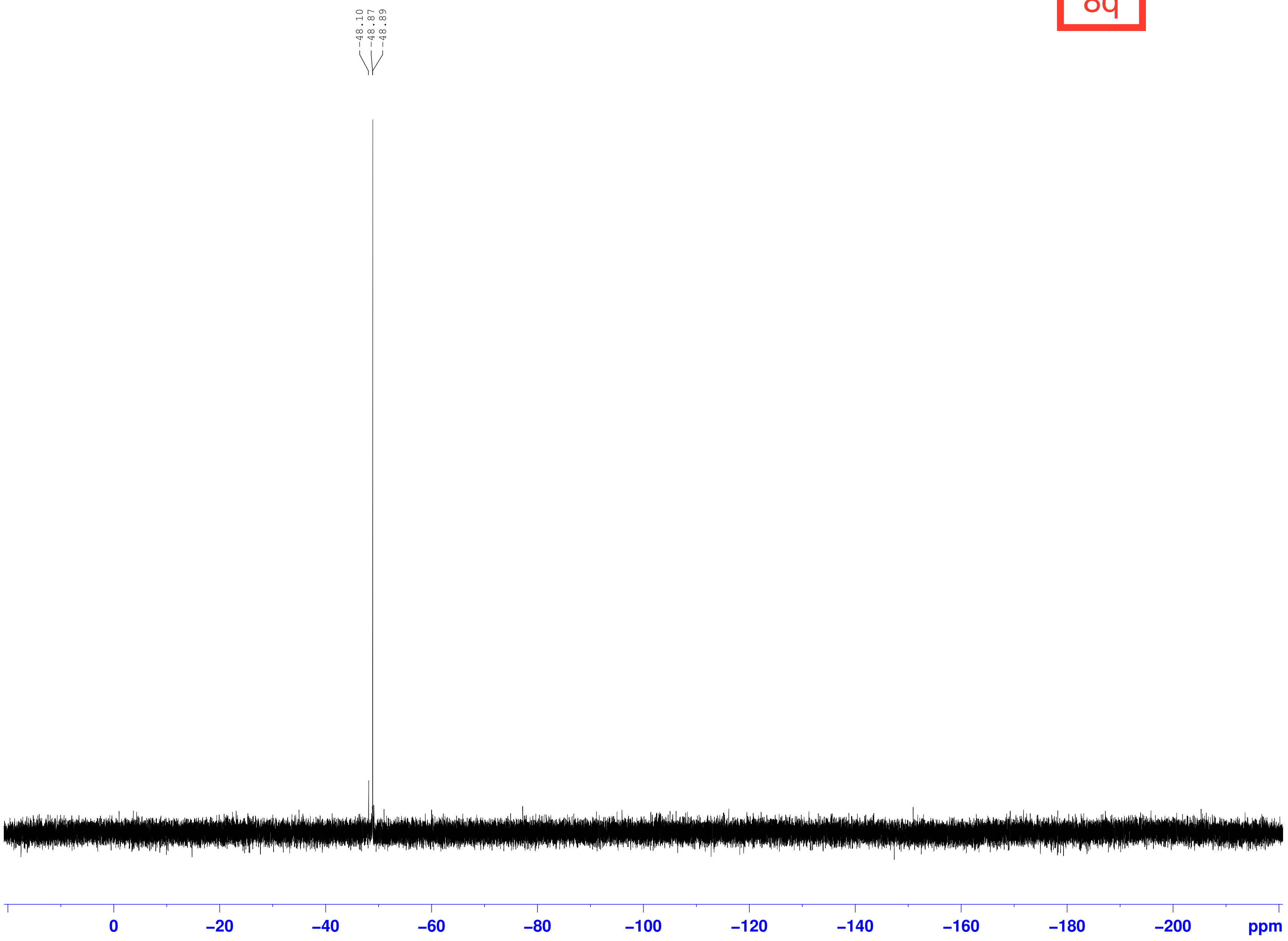

CARDIFF UNIVERSITY
PRIFYSGOL CAERDYDD

NAME	CM-SK97p	
EXPNO	3	
PROCNO	1	
Date_	20140922	
Time	10.26	
INSTRUM	Avance500	
PROBHD	5 mm QNP 1H/13	
PULPROG	zgfhigqn	
TD	131072	
SOLVENT	DMSO	
NS	16	
DS	4	
SWH	113636.367	Hz
FIDRES	0.866977	Hz
AQ	0.5767668	sec
RG	4100	
DW	4.400	use
DE	6.00	use
TE	288.8	K
D1	1.00000000	sec
D11	0.03000000	sec
D12	0.00002000	sec
TD0	1	

======== CHANNEL f1 =======

NUC1	19F	
P1	18.60	use
PL1	-1.50	dB
PL1W	11.14113998	W
SFO1	470.5453180	MHz

======== CHANNEL f2 =======

CPDPRG2	waltz16	
NUC2	1H	
PCPD2	80.00	use
PL2	-2.00	dB
PL12	14.85	dB
PL2W	14.33185768	W
PL12W	0.29600734	W
SFO2	500.1320005	MHz
SI	65536	
SF	470.5923770	MHz
WDW	EM	
SSB	0	
LB	0.30	Hz
GB	0	
PC	1.40	

8q

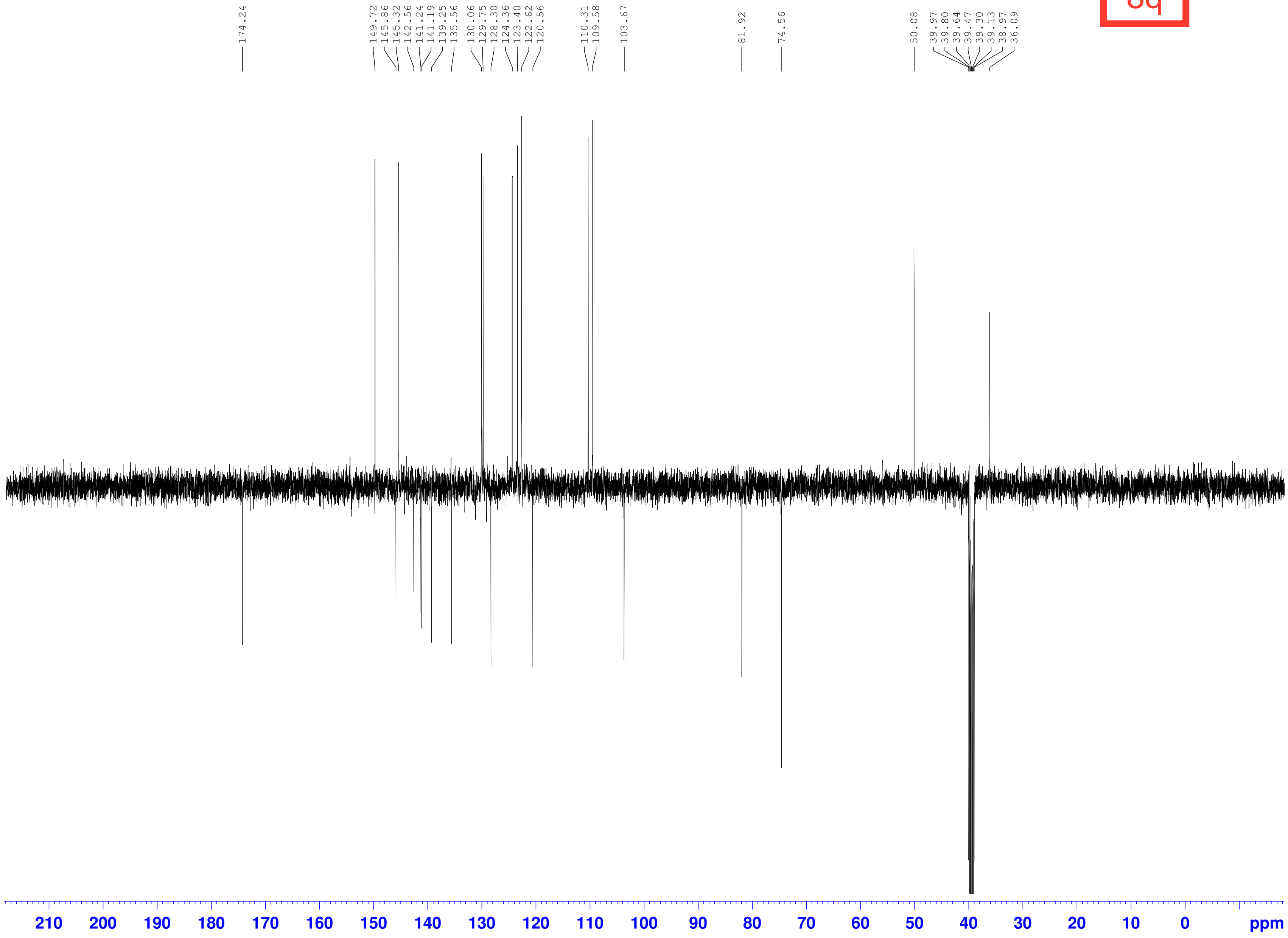

CARDIFF UNIVERSITY PRIFYSGOL CAERDYDD

```
NAME              CM-SK97p
EXPNO                   13
PROCNO                   1
Date_             20141112
Time                 19.11
INSTRUM         Avance500
PROBHD    5 mm QNP 1H/13
PULPROG            pendant
TD                   65536
SOLVENT               DMSO
NS                    3072
DS                       4
SWH          29761.904 Hz
FIDRES        0.454131 Hz
AQ           1.1010548 sec
RG                    3250
DW              16.800 usec
DE               12.00 usec
TE               298.1 K
CNST2      145.0000000
D1          2.00000000 sec
D4          0.00172414 sec
D12         0.00002000 sec
D15         0.00431034 sec
D20         0.00345000 sec
TD0                     24

======== CHANNEL f1 ========
NUC1                   13C
P1                7.20 usec
P2               14.40 usec
PL1              -2.00 dB
PL1W      101.27846527 W
SFO1       125.7703643 MHz

======== CHANNEL f2 ========
CPDPRG2            waltz16
NUC2                    1H
P3               11.50 usec
P4               23.00 usec
PCPD2            80.00 usec
PL2              -2.00 dB
PL12             14.85 dB
PL2W       14.33185768 W
PL12W       0.29600734 W
SFO2       500.1320005 MHz
SI                   32768
SF         125.7578519 MHz
WDW                     EM
SSB                      0
LB                1.00 Hz
GB                       0
PC                    1.40
```

SK97P MW=463?
(MeOH)/MeOH + NH4OAc
C24H15F2N3O5

EPSRC National Facility Swansea
LTQ Orbitrap XL

8q

Dr AD Westwell
05/08/2015 16:28:56

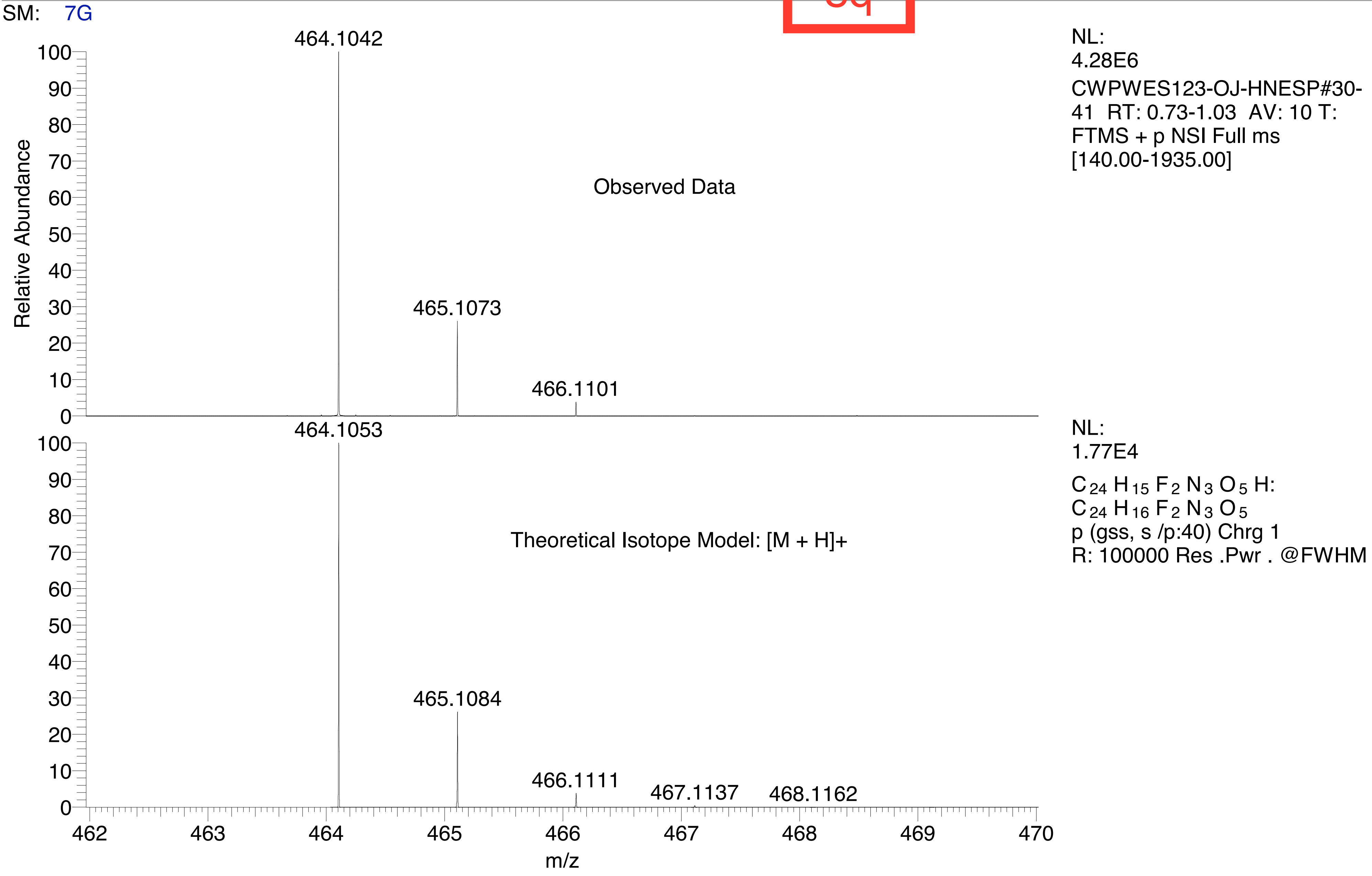

Supplement: Supplementary file 1 [file mmc1.pdf]
